# Gallbladder microbial species and host bile acids biosynthesis linked to cholesterol gallstone comparing to pigment individuals

**DOI:** 10.3389/fcimb.2024.1283737

**Published:** 2024-03-11

**Authors:** Xinpeng Zhang, Junqing Hu, Yi Li, Jichao Tang, Kaijin Yang, Ayan Zhong, Yanjun Liu, Tongtong Zhang

**Affiliations:** ^1^ General Surgery Day Ward, Department of General Surgery, The Third People’s Hospital of Chengdu, Affiliated Hospital of Southwest Jiaotong University & The Second Affiliated Hospital of Chengdu, Chongqing Medical University, Chengdu, China; ^2^ Center of Gastrointestinal and Minimally Invasive Surgery, Department of General Surgery, The Third People’s Hospital of Chengdu, Affiliated Hospital of Southwest Jiaotong University & The Second Affiliated Hospital of Chengdu, Chongqing Medical University, Chengdu, China; ^3^ The Center of Obesity and Metabolic Diseases, Department of General Surgery, The Third People’s Hospital of Chengdu, Affiliated Hospital of Southwest Jiaotong University & The Second Affiliated Hospital of Chengdu, Chongqing Medical University, Chengdu, China; ^4^ Medical Research Center, The Third People’s Hospital of Chengdu, Affiliated Hospital of Southwest Jiaotong University & The Second Affiliated Hospital of Chengdu, Chongqing Medical University, Chengdu, China

**Keywords:** cholesterol gallstone, gallbladder microbiota, 2bRAD-M, bile acids metabolism, LC-MS metabolomics, multiomics

## Abstract

Gallstones are crystalline deposits in the gallbladder that are traditionally classified as cholesterol, pigment, or mixed stones based on their composition. Microbiota and host metabolism variances among the different types of gallstones remain largely unclear. Here, the bile and gallstone microbial species spectra of 29 subjects with gallstone disease (GSD, 24 cholesterol and 5 pigment) were revealed by type IIB restriction site-associated DNA microbiome sequencing (2bRAD-M). Among them (21 subjects: 18 cholesterol and 3 pigment), plasma samples were subjected to liquid chromatography–mass spectrometry (LC-MS) untargeted metabolomics. The microbiome yielded 896 species comprising 882 bacteria, 13 fungi, and 1 archaeon. Microbial profiling revealed significant enrichment of *Cutibacterium acnes* and *Microbacterium* sp005774735 in gallstone and *Agrobacterium pusense* and *Enterovirga* sp013044135 in the bile of cholesterol GSD subjects. The metabolome revealed 2296 metabolites, in which malvidin 3-(6’’-malonylglucoside), 2-Methylpropyl glucosinolate, and ergothioneine were markedly enriched in cholesterol GSD subjects. Metabolite set enrichment analysis (MSEA) demonstrated enriched bile acids biosynthesis in individuals with cholesterol GSD. Overall, the multi-omics analysis revealed that microbiota and host metabolism interaction perturbations differ depending on the disease type. Perturbed gallstone type-related microbiota may contribute to unbalanced bile acids metabolism in the gallbladder and host, representing a potential early diagnostic marker and therapeutic target for GSD.

## Introduction

Cholelithiasis (also referred to as gallstone disease, GSD) is common worldwide. Individuals with GSD have an increased risk for gallbladder and liver disease and an increased level of inflammation (Ryu et al., 2016; Arrese et al., 2018; Rodríguez-Antonio et al., 2020). GSD can profoundly impact one’s life and work performance due to the pain symptom. However, the underlying mechanisms contributing to gallstones influenced by the gallbladder microbiome remain unclear. Approximately two-thirds of gallstones are the cholesterol type, and the rest are mainly pigment stones (Portincasa and Wang, 2012). Cholesterol stones are usually yellow-green in color and are predominantly composed of cholesterol while pigment stones are brown-dark in color and are made of pigment bilirubin (Kose et al., 2018). Microbiota and host metabolism variances among the different types of gallstones remain largely unclear. Thus, understanding such variances may provide microbial and physiological insights into the formation of different types of gallstones.

The human microbiome, also known as the second genome, can influence metabolic homeostasis through microbiota–host interactions in physiological and pathological conditions (Fan and Pedersen, 2021). Normally, the dynamically stable microbiota plays a vital role in sustaining the balance of host inflammation, thus positively regulating phycological functions including immune responses (Belkaid and Hand, 2014). However, the microbiota inhabiting the gallbladder of humans is substantially under-studied. Our prior studies of the bacterial and fungal fractions of the gallbladder microbiome in GSD revealed substantial trans-kingdom interactions between various microbial taxa at the genus level (Hu et al., 2023). Importantly, high taxa resolution will reveal finer details and the distribution pattern of opportunistic pathogens in the gallbladder microbiota. However, obtaining accurate, species-resolution, landscape-like taxonomic profiles from bile and gallstone samples is challenging due to their low-biomass. Given that, a cost-effective sequencing strategy—type IIB restriction site-associated DNA microbiome sequencing (2bRAD-M)—has been proposed for assessing such challenging samples (Wang et al., 2012; Sun et al., 2022). The ability to profile low-biomass microbiomes at the species level is pivotal to uncovering the crucial roles of the gallbladder microbiota underlying human health.

Furthermore, metabolomic profiles (metabolomics) provide a comprehensive readout of human (patho)physiology and advance our understanding of disease etiology (Ramautar et al., 2013; Gonzalez-Covarrubias et al., 2022). This promising approach is central to disease prevention and diagnosis since the human blood metabolome mirrors the overall physiological state (Johnson et al., 2019). However, the blood metabolomic profiles of individuals with cholesterol or pigment GSD remain unknown.

Herein, the present study for the first time further explores the profile of the gallbladder microbiota at the species level and its links with the plasma metabolites by integrating microbiome (2bRAD-M sequencing) and metabolome (untargeted LC-MS metabolomics), respectively. This wealth of multi-omics data provides an opportunity to investigate associations between the gallbladder microbiome, health, and the type of gallstone.

## Materials and methods

### Subjects and sample collection

We recruited 29 individuals with gallstone (gallbladder-stone) and chronic cholecystitis in the present study (**Table 1**; **Supplementary Table 1**). Their age ranged from 21 to 82 years, averaging 42.4 years. Most were female (22/29), and 24 of 29 had cholesterol stones (the remaining subjects had pigment-type stones).

**Table 1:** Demographic and clinical details of GSD subjects in current multiomics study.

Data	Factor	Value
Microbiome	Sample size	29
	Age (year)^a^	42.4 (21-82)
	Male/Female	7/22
	Cholesterol/Pigment	24/5
	Bile/Gallstone	29/27
Metabolome	Sample size	21
	Age (year)^a^	41.9 (23-82)
	Male/Female	5/16
	Cholesterol/Pigment	18/3

a mean (min-max).

**Figure 1:**
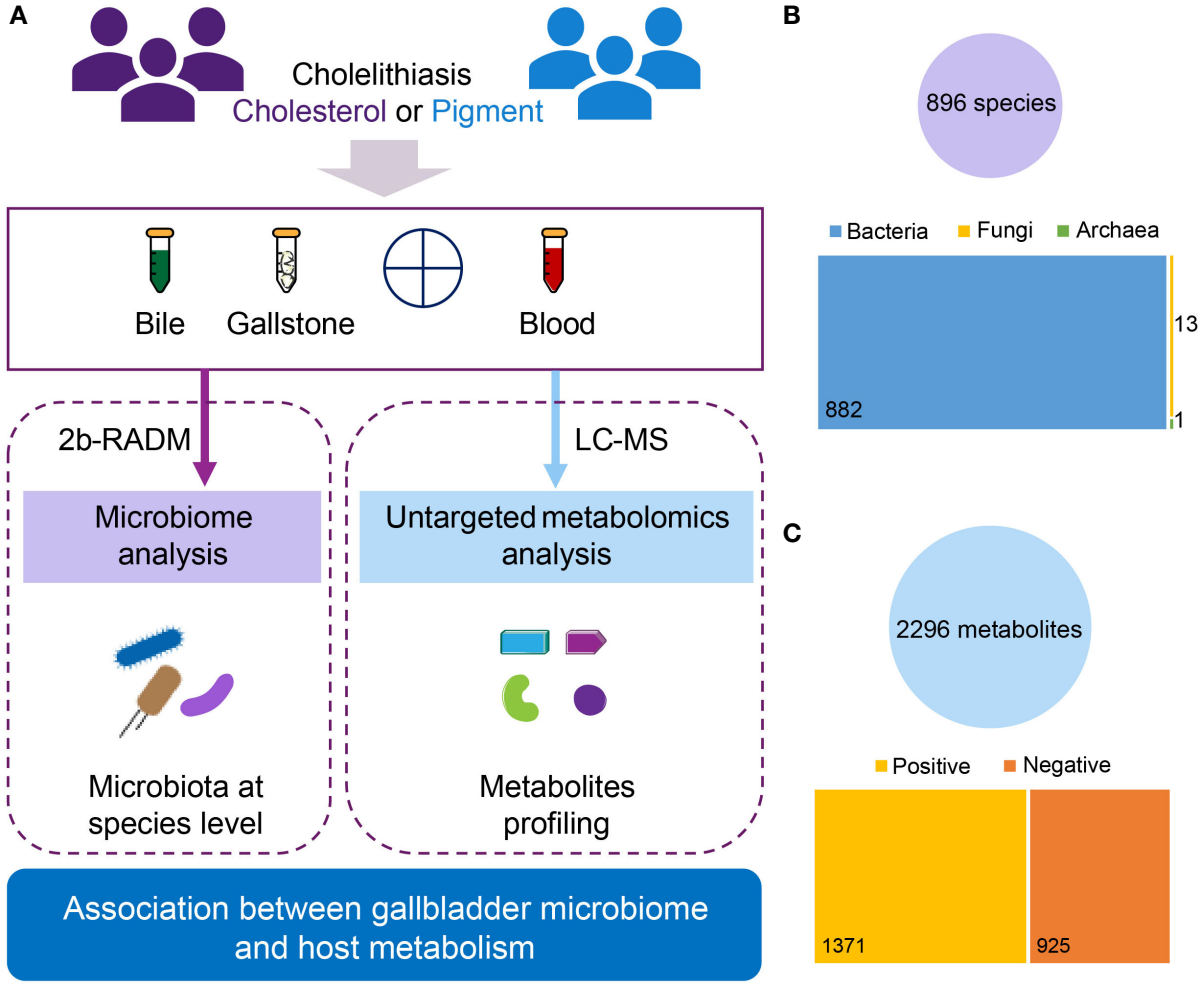
Overview of the current multiomics study. **(A)** Diagram of study design. A total of 29 subjects diagnosed with cholesterol (n = 24) or pigment (n = 5) GSD were assessed. For the gallbladder microbiota analysis, bile and gallstone samples were collected from all patients, yielding 56 samples (27 paired bile and gallstone samples, and 2 bile samples only) for 2bRAD-M. For the plasma metabolomic analysis, plasma samples from 21 (18 cholesterol and 3 pigment) of those 29 individuals were used for LC–MS. **(B)** Overview of microbial data characterized by 2bRAD-M. In total, 896 species (882 bacteria, 13 fungi, and 1 archaeon) were identified from 54 bile and gallstone samples (2 samples were removed during quality control). **(C)** Overview of metabolic data characterized by LC–MS. In total, 2296 metabolites (1371 positive and 925 negative compounds) were identified from 21 plasma samples.

The gallstone and bile samples were collected after gallbladder removal surgery (laparoscopic cholecystectomy) as previously described (Hu et al., 2023). In brief, bile samples (2–5 mL) were immediately collected from each patient after gallbladder removal. Stones (3–5 pellets) were washed in sterile stroke-physiological saline solution prior to collection from removed gallbladder. All samples were strictly sterilely collected and stored at −80°C until further use. In total, 56 samples (27 paired bile and gallstone samples and 2 individual bile samples) were acquired for the microbiota analysis. Blood was also collected to assess plasma metabolites. Twenty-one plasma samples were assessed using an untargeted metabolomics approach (**Table 1**; **Supplementary Table 1**; **Figure 1A**).

### Microbial DNA extraction, library preparation, and sequencing

The genomic DNA of bile (200 µL) and stone (approximately 200 mg) microbiota were extracted using a QIAamp DNA Mini Kit and QIAamp Fast DNA Stool Mini Kit (QIAGEN, Germany) respectively following the manufacturer’s instructions. DNA concentrations were detected using a NanoDrop 2000 (Thermo Fisher Scientific, Waltham, MA, USA). DNA samples were stored at −80°C until required for experiments. The 2bRAD libraries were prepared according to previous studies and purified with the QIAquick PCR purification kit (QIAGEN, Germany) (Lam et al., 2022; Sun et al., 2022). Microbial sequencing was conducted on an Illumina Novaseq PE150 platform by OE Biotech Co., Ltd. (Shanghai, China). Briefly, DNA (1 pg-200 ng) was digested with 4 U of the enzyme *BcgI* (NEB) for 3 h at 37°C. Subsequently, the adaptors were ligated to the DNA fragments performed by combining 5 µL of digested DNA with 10 µL of a ligation master mix containing 0.2 µM each of two adaptors and 800 U T4 DNA ligase (NEB) at 4°C for 12 h. Then, ligation products were amplified, and PCR products were subjected to 8% polyacrylamide gel. Bands of approximately 100 bp were excised from the polyacrylamide gel, and the DNA was diffused from the gel in nuclease-free water for 12 h at 4°C. Sample-specific barcodes were introduced by PCR with platform-specific barcode-bearing primers. Each 20 µL PCR contained 25 ng of gel-extracted PCR product, 0.2 µM of each primer, 0.3 mM dNTP, 1×Phusion HF buffer and 0.4 U Phusion high-fidelity DNA polymerase (NEB). All adaptor and primer sequences used in this study can be found in **Supplementary Table 2**.

### Microbial data processing, diversity analysis and taxonomic assignment

Raw reads (only R1 reads were used in the current study) were filtered to extract the digested fragments (“enzyme reads”) using the *BcgI* restriction enzyme recognition site. Clean reads were obtained from enzyme reads using two criteria. First, reads with >8% unknown bases (N) were filtered; second, reads with of low-quality bases exceeding 20% (Q-value < 30) were removed. Microbial diversity was analyzed using the Shannon (Shannon, 1997), Simpson (Simpson, 1949), and Chao1 (Chao, 1984) indices (alpha diversity) and Bray-Curtis distance (Sørensen et al., 1948) (beta diversity). The 2bRAD-M pipeline (https://github.com/shihuang047/2bRAD-M) was applied to perform the taxonomic profiling using a unique 2bRAD tag database (2b-Tag-DB) comprising taxa-specific *BcgI*-derived tags identified from 173,165 microbial genomes.

### Metabolomics profiling of plasma samples

A quality control (QC) sample was obtained by mixing and blending equal volumes (10 µL) of each plasma sample to estimate the mean profile representing all the analytes encountered during analysis. A Dionex U3000 UHPLC/QE plus quadrupole-Orbitrap mass spectrometer equipped with a heated electrospray ionization (ESI) source (Thermo Fisher Scientific, Waltham, MA, USA) was used for both ESI positive and ESI negative ion modes. An ACQUITY UPLC HSS T3 column (1.8 μm, 2.1 × 100 mm) was used. All the samples were stored at 4°C during the analysis. The binary gradient elution system consisted of (A) water (containing 0.1% formic acid, v/v) and (B) acetonitrile (containing 0.1% formic acid, v/v) and separation was achieved using the following gradient: 0.01 min, 5% B; 2 min, 5% B; 4 min, 30% B; 8 min, 50% B; 10 min, 80% B; 14 min, 100% B; 15 min, 100% B; 15.1 min, 5% and 18 min, 5% B. The flow rate was 0.35 mL/min and column temperature was 45°C. The injection volume was 2 µL. The mass resolution was set at 70,000 for the full MS scans and 17,500 for HCD MS/MS scans and collision energy was set at 10, 20 or 40 eV.

### LC–MS data processing

The raw LC–MS data were processed using Progenesis QI V2.3 (Nonlinear, Dynamics, Newcastle, UK) for baseline filtering, peak identification, integral, retention time correction, peak alignment, and normalization. Metabolite identification was conducted based on the precise mass-to-charge ratio (M/z), secondary fragments, and isotopic distribution using the Human Metabolome Database (HMDB), Lipidmaps (V2.3), Metlin, EMDB, PMDB, and self-built databases (Shanghai Lu-Ming Biotech Co., Ltd., Shanghai, China) for the qualitative analysis. The extracted data were then further processed by removing peaks with missing values (ion intensity = 0) in over 50% of the groups and replacing the zero value with half of the minimum value and screening according to the qualitative results of the metabolite. Metabolites with scores below 36 (out of 60) points were removed. Finally, a data matrix (2296 metabolites) was generated by combining the positive and negative ion data and analyzed by MetaboAnalyst 5.0 (https://www.metaboanalyst.ca/) (Pang et al., 2021).

### Statistical analysis

Wilcox test and Adonis (PERMANOVA) were used for the microbial alpha and beta diversity analyses, respectively. A linear discriminant analysis (LDA) effect size (LEfSe) was employed for the microbial biomarker analysis. The strategy “one-against-one” (stricter) was chosen for LEfSe. Metabolomics data were statistically analyzed using MetaboAnalyst (V5.0). Metabolites with a variable importance in projection (VIP) score ≥ 1.0 with a *P* value < 0.05 were considered significant. Classical univariate receiver operating characteristic (ROC) curve analysis was used to identify potential metabolite biomarkers and evaluate their performance. The significance cutoff was set at *P* < 0.05. Other statistical cutoffs are individually indicated in each figure where appropriate.

## Results

### Overview of gallbladder microbiota data and plasma metabolomics profiling

Overall, we identified 896 species by 2bRAD-M of both bile and gallstone samples, including 882 bacteria, 13 fungi and 1 archaeon (**Figure 1B**). Additionally, 2296 metabolites were characterized by LC-MS metabolomics. Among these metabolites, approximately 60% (1371/2296) were positive compounds, whereas 925 were negative (**Figure 1C**; **Supplementary Figure 1A**). Lipids and lipid-like molecules, Organic acids and derivatives, and Organoheterocyclic compounds were the top 3 superclasses of detected metabolites (**Supplementary Figure 1B**). Fatty acyls, carboxylic acids and derivatives, organooxygen compounds, steroids and steroid derivatives, and prenol lipids were the top 5 metabolic classes (**Supplementary Figure 1C**). Amino acids, peptides, and analogues, fatty acids and conjugates, and fatty acid esters were the main subclasses (**Supplementary Figure 1D**).

### Gallstone type-specific changes to gallbladder microbiota are dependent of sample type

**Figure 2:**
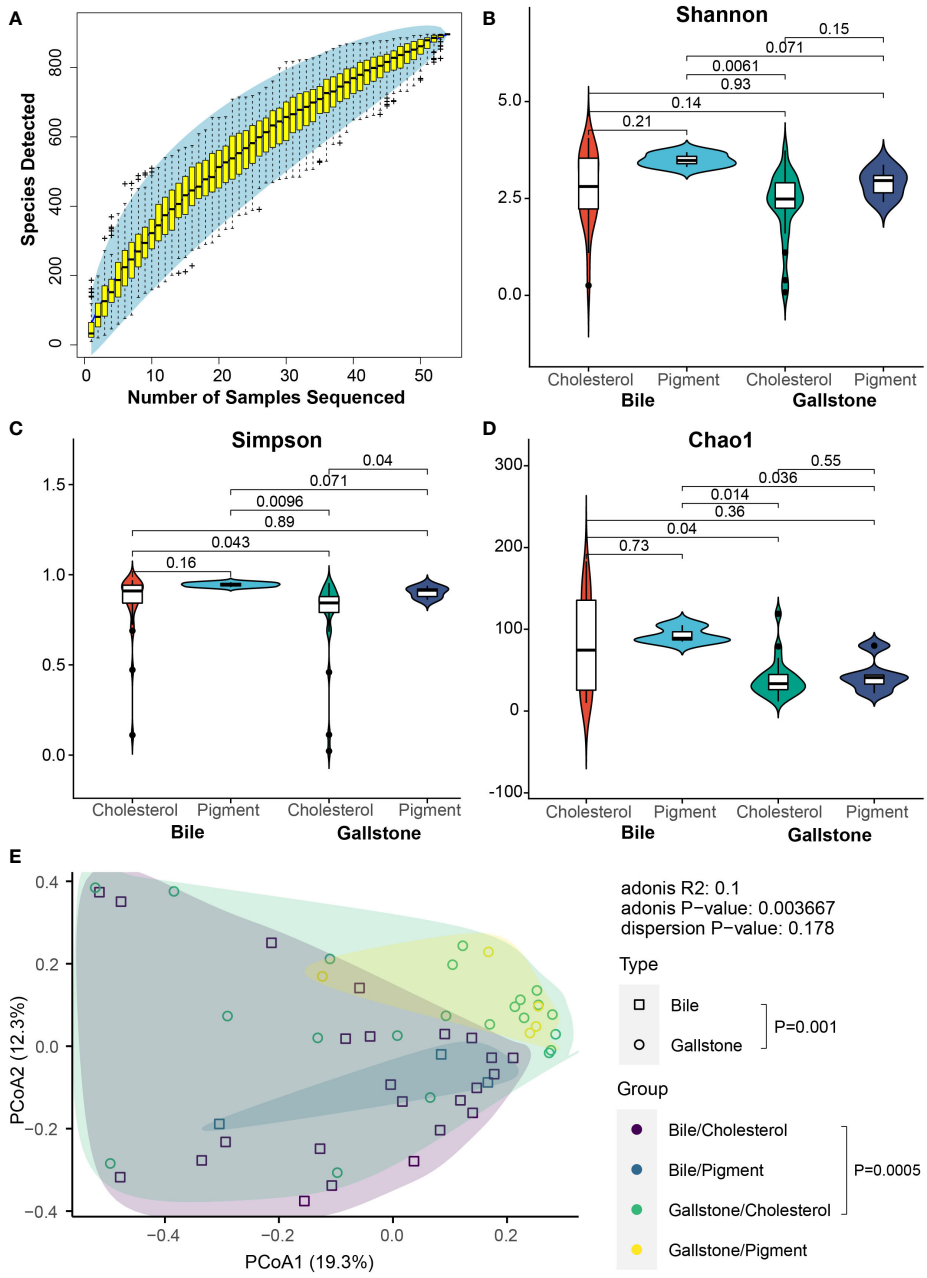
Alteration of microbial diversity between cholesterol and pigment GSD patients depends on sample type. **(A)** Coverage of members in the gallbladder microbiota. Rarefaction curve of detected microbial species in the gallbladder microbiota reaches saturation as the sample number increases. **(B–D)** Gallbladder microbial alpha diversity among cholesterol/bile (n = 24), cholesterol/gallstone (n = 22), pigment/bile (n = 3) and pigment/gallstone (n = 5) groups by Shannon **(B)**, Simpson **(C)**, and Chao1 **(D)** indices. The statistical analysis was performed using the Wilcoxon test. **(E)** Principal coordinate analysis (PCoA) of the Bray–Curtis distance among cholesterol/bile (n = 24), cholesterol/gallstone (n = 22), pigment/bile (n = 3) and pigment/gallstone (n = 5) groups. The statistical analysis was performed using a pairwise PERMANOVA (Adonis) test.

We first examined microbial diversity to explore the microbial differences in the gallbladder of subjects with cholesterol and pigment-type GSD. The rarefaction curve of detected microbial species in the gallbladder microbiota reached saturation as the sample number increased, indicating that the gallbladder microbiota in our population captured most gallbladder microbial members (**Figure 2A**). Generally, the microbial alpha diversity in bile samples was higher than that in the gallstone samples (**Figures 2B–D**). Although the Shannon (**Figure 2B**, *P* = 0.21 in bile, *P* = 0.15 in gallstone) and Chao1 (**Figure 2D**, *P* = 0.73 in bile, *P* = 0.55 in gallstone) indices showed no significant change between cholesterol and pigment GSD subjects regardless of the sample type, a marked difference was present in the gallstone versus bile sample based on the Simpson index (**Figure 2C**, *P* = 0.16 in bile, *P* = 0.04 in gallstone). Moreover, the microbial diversity in bile from pigment GSD individuals was significantly higher than that in gallstones from cholesterol GSD subjects (**Figures 2B–D**). The microbial beta diversity based on a principal coordinates analysis (PCoA) of the Bray-Curtis distance showed distinct clustering of bile and gallstone samples from all subjects (**Figure 2E**, *P* = 0.001), and cholesterol GSD subjects (**Figure 2E**, *P* = 0.0005).

**Figure 3:**
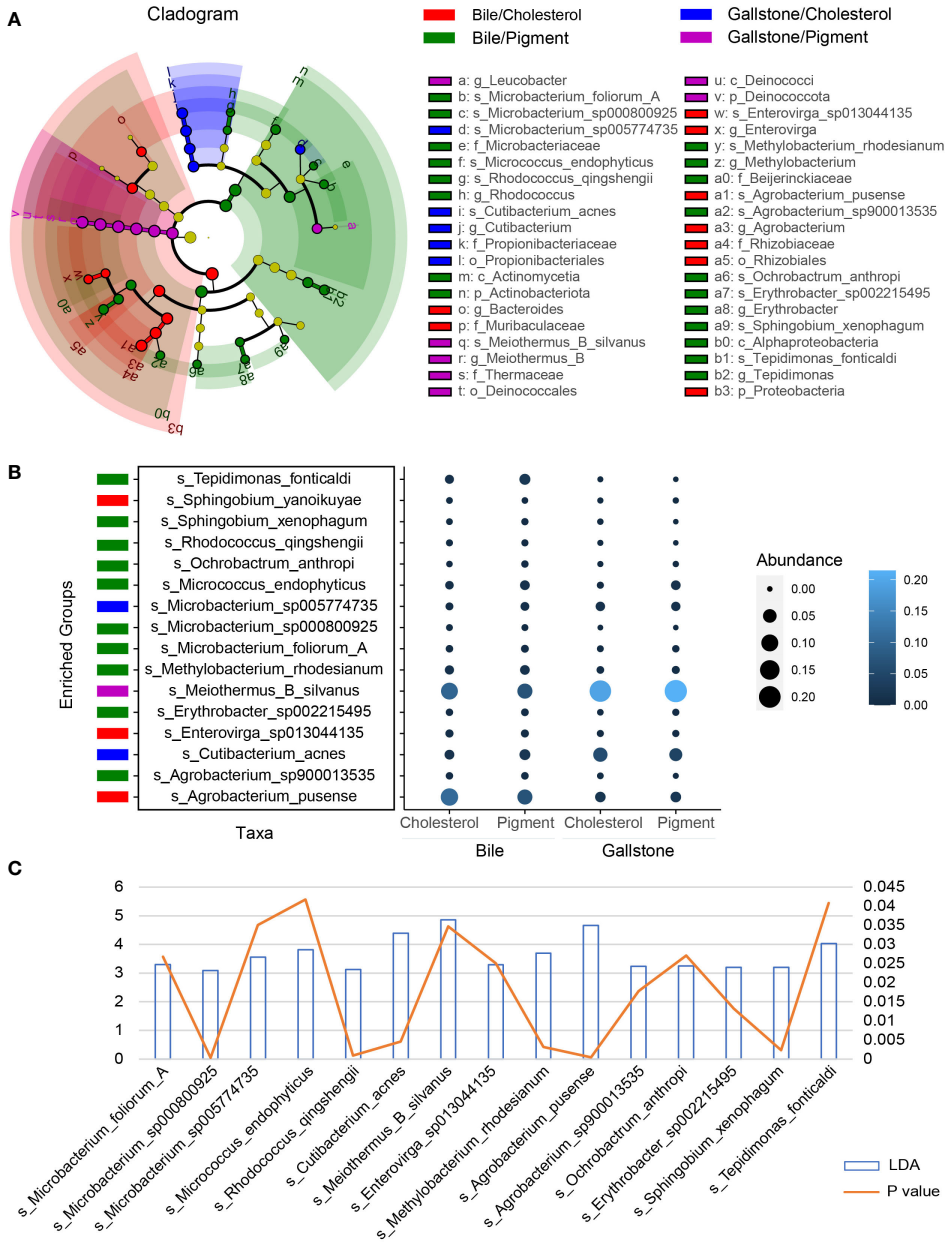
Gallstone type-related changes in gallbladder microbial species depends on sample type. **(A)** LEfSe cladogram showing the differing abundance of microbiota among cholesterol/bile (n = 24), cholesterol/gallstone (n = 22), pigment/bile (n = 3) and pigment/gallstone (n = 5) groups. The diameter of each circle is proportional to the abundance (LDA score (log 10) > 3.0). **(B)** Bubble plot showing the representative microbial species markers among the four groups. **(C)** Bar and line graphs showing the LDA scores (log 10) and *P* values of these representative species.

2bRAD-M revealed a large group of microbial species across 20 phyla in the gallbladder, including bacteria, fungi, and archaea (**Supplementary Figure 2A**). A total of 896 species were identified (**Figure 1B**). *Meiothermus silvanus*, *Acinetobacter johnsonii*, *Agrobacterium pusense*, *Vulcaniibacterium thermophilum*, *Escherichia flexneri*, *Streptococcus agalactiae*, *Cutibacterium acnes*, *Streptococcus mutans*, *Klebsiella pneumoniae*, and *Dietzia maris* were the dominant microbial species (**Supplementary Figure 2B**). We further accessed the microbial signatures associated with type of gallstone by LEfSe (**Figure 3**; **Supplementary Figure 3**). The highly abundant species *Meiothermus silvanus* (genus *Meiothermus*) was substantially enriched in the gallstone of pigment GSD subjects (LDA (log 10) = 4.85, *P* = 0.0347). *Cutibacterium acnes* (genus *Cutibacterium*, LDA (log 10) = 4.39, *P* = 0.00458) and *Microbacterium sp005774735* (LDA (log 10) = 3.56, *P* = 0.0351) were biomarkers of gallstone in cholesterol GSD subjects. Ten species, including *Tepidimonas fonticaldi* (LDA (log 10) = 4.03, *P* = 0.0408), *Micrococcus endophyticus* (LDA (log 10) = 3.82, *P* = 0.0418), and *Methylobacterium rhodesianum* (LDA (log 10) = 3.69, *P* = 0.00319), were enriched in the bile samples from pigment GSD subjects, while *Agrobacterium pusense* (LDA (log 10) = 4.66, *P* = 0.000453), and *Enterovirga sp013044135* (LDA (log 10) = 3.29, *P* = 0.0251) were enriched in the bile samples from cholesterol GSD subjects. These results demonstrate that alterations in the gallbladder microbiota differ in bile and gallstone samples from subjects with different gallstone types.

### Significant alterations in plasma metabolites between cholesterol and pigment GSD individuals

**Figure 4:**
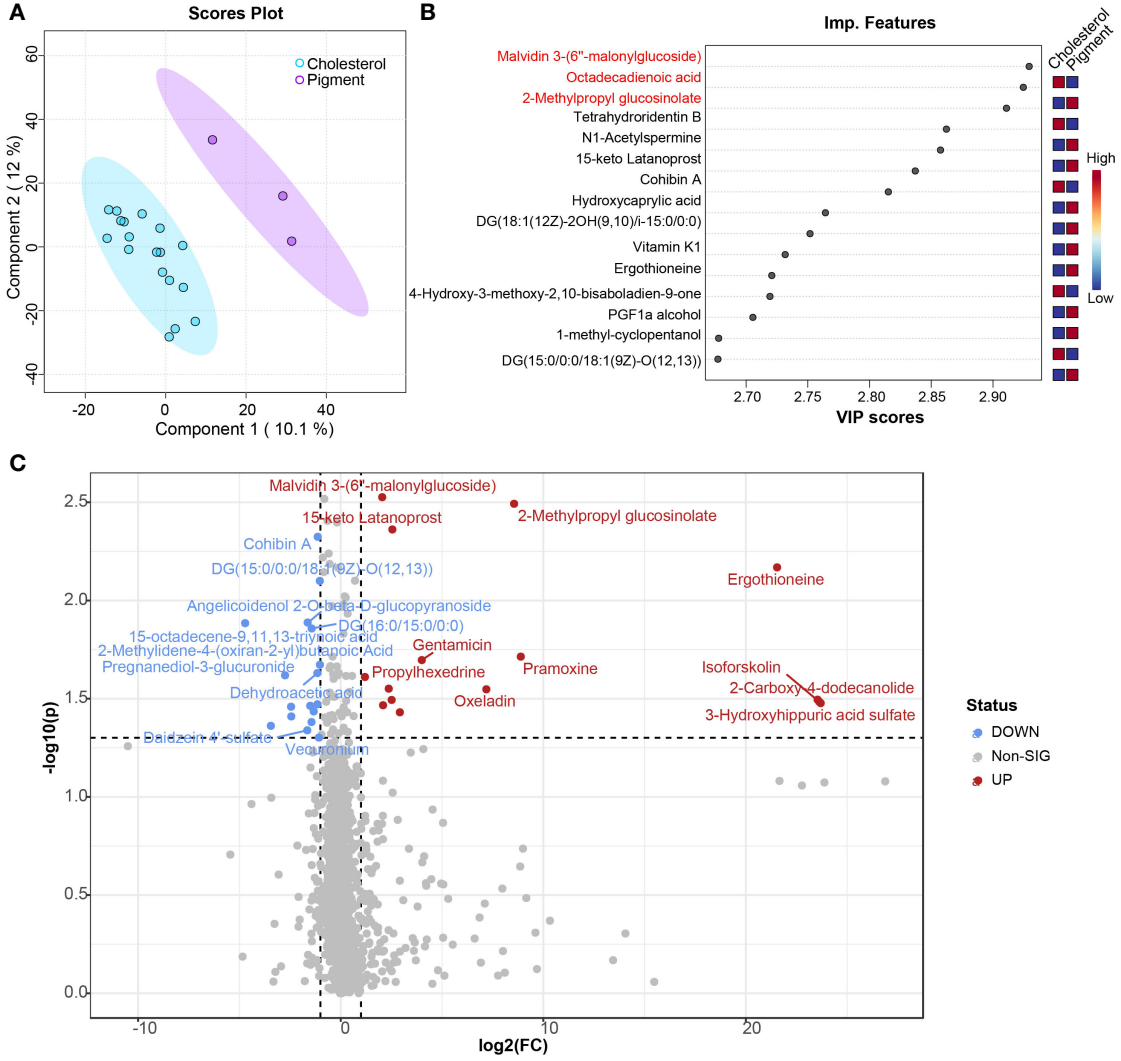
Comparison of plasma metabolome between cholesterol and pigment GSD subjects. **(A)** Scores plot of results from partial least squares–discriminant analysis (PLS-DA). **(B)** Top 15 important metabolic features between cholesterol (n = 18) and pigment (n = 3) GSD patients detected by PLS-DA ranked by variable importance projection (VIP) scores. **(C)** Volcano plot analysis of different metabolites between cholesterol (n = 18) and pigment (n = 3) GSD patients. Fold change (FC) > 2, *P*-value threshold < 0.05. Red points represent metabolites with increased abundance in cholesterol GSD patients; blue points represent metabolites with increased abundance in pigment GSD patients.

Untargeted LC-MS metabolomics was performed to assess the metabolic profiling differences between cholesterol and pigment GSD subjects. In total, 2296 metabolites were detected in the plasma of cholesterol and pigment GSD subjects. Partial least squares discriminant analysis (PLS-DA) confirmed the large difference observed in the plasma metabolome of cholesterol or pigment GSD subjects (**Figures 4A**). The top 15 metabolites detected by PLS-DA between cholesterol and pigment GSD subjects are shown in **Figure 4B**. Metabolites malvidin 3-(6’’-malonylglucoside) (0.64_1157.2640m/z, **Figure 4B**; **Supplementary Figure 4A**), 2-Methylpropyl glucosinolate (4.76_356.0497m/z, **Figure 4B**; **Supplementary Figure 4B**), and ergothioneine (7.16_269.0596m/z, **Figure 4B**; **Supplementary Figure 4C**) were enriched in plasma from cholesterol GSD subjects, whereas 2-hydroxy-9Z,12Z-Octadecadienoic acid (15.23_279.2315m/z, **Figure 4B**) was enriched in pigment GSD subjects (VIP score > 2). Moreover, the differential metabolites volcano plot showed that 17 metabolites, including 15.23_279.2315m/z and 15-octadecene-9,11,13-triynoic acid (6.02_248.1643m/z, **Figure 4C**, **Supplementary Figure 4D**) were enriched in pigment GSD subjects, while 15 metabolites, including malvidin 3-(6’’-malonylglucoside), 2-Methylpropyl glucosinolate, and ergothioneine, were enriched in cholesterol GSD subjects (**Figure 4C**; **Supplementary Figure 4**; **Supplementary Table 3**, |Log2(FC)| > 1, *P* < 0.05).

**Figure 5:**
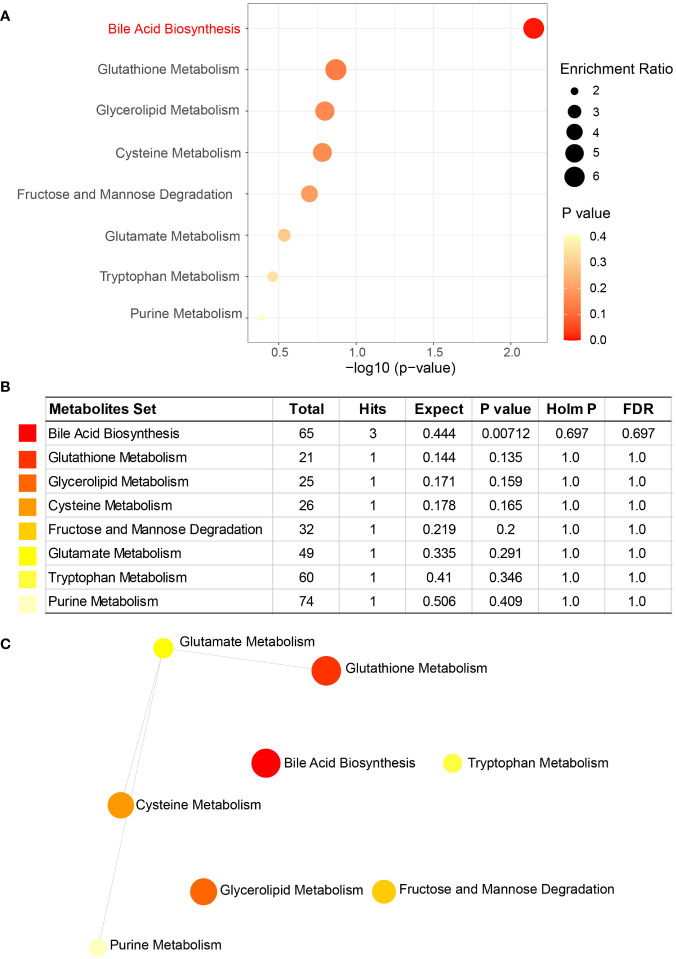
Metabolite set enrichment analysis (MSEA) of enriched metabolites in cholesterol GSD subjects. **(A)** Key pathways related to cholesterol gallstones. **(B)** Details of key pathways associated with cholesterol gallstones. **(C)** Network of key pathways enriched in cholesterol gallstones. Enrichment Ratio computed by Hits/Expected, where hits = observed hits; expected = expected hits.

Metabolite set enrichment analysis (MSEA) was performed using MetaboAnalyst to assess the functions of altered metabolites between cholesterol and pigment GSD subjects. Bile acid biosynthesis (*P* = 0.00712) is significantly enriched in cholesterol GSD individuals while caffeine metabolism (*P* = 0.00307) is markedly enriched in pigment GSD individuals (**Figures 5A, B**; **Supplementary Figures 5A, B**). In addition, dysfunction of some amino acids (cysteine, glutamate, and tryptophan) metabolism was found in cholesterol GSD subjects although the difference was not significant (**Figures 5A, B**). The three amino acids and purine (metabolism) are connected to a network in cholesterol GSD subjects, as shown in **Figure 5C**. Altogether, these results imply that cholesterol GSD is linked to dysfunctional bile acid metabolism.

### Potential plasma metabolites biomarkers for distinguishing cholesterol gallstone from pigment

**Figure 6:**
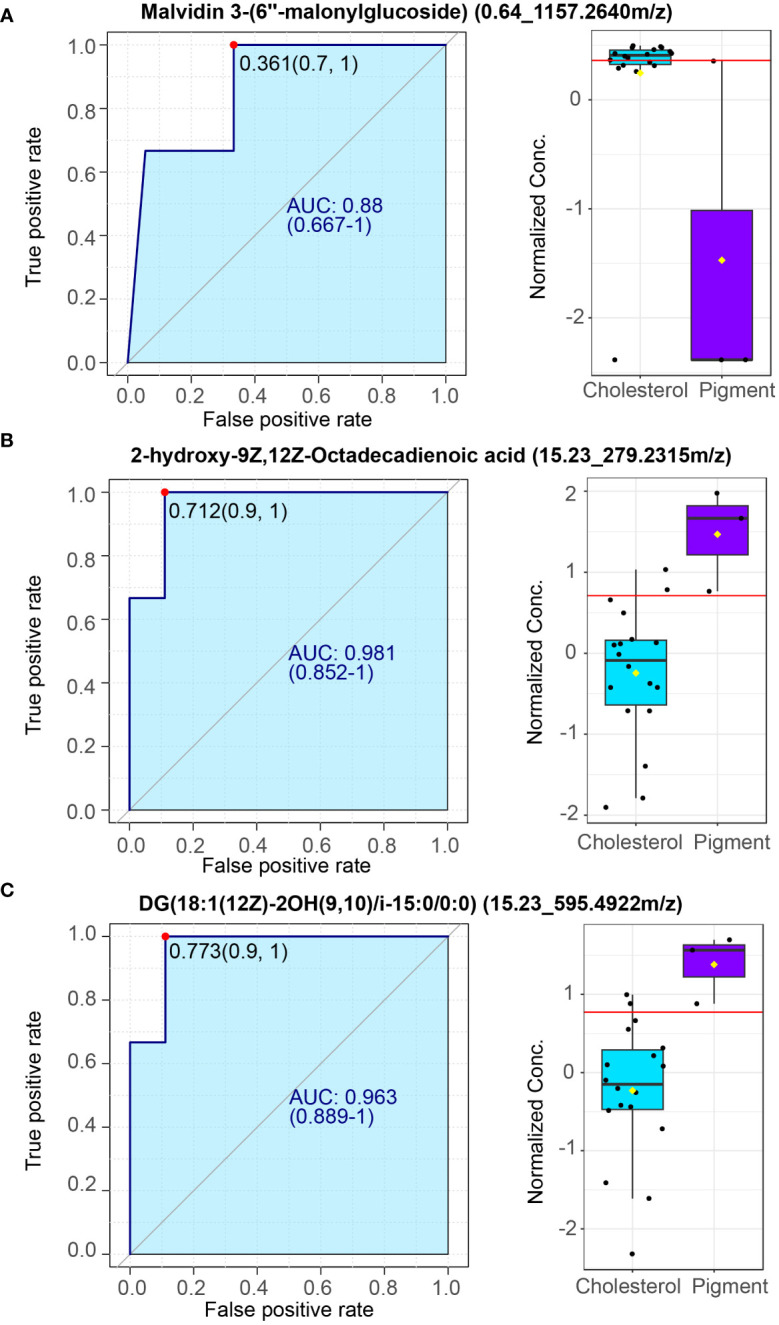
Potential plasma markers of cholesterol and pigment gallstones. **(A)** Plasma marker malvidin 3-(6’’-malonylglucoside) for cholesterol gallstones. **(B, C)**. Plasma markers 2-hydroxy-9Z,12Z-Octadecadienoic acid **(B)** and DG (18:1(12Z)-2OH(9,10)/i-15:0/0:0) **(C)** for pigment gallstones. Left: Receiver operating characteristic (ROC) curve. Right: normalized concentrate of plasma markers between cholesterol (n =18) and pigment (n = 3) GSD patients.

To further extract whether the metabolites can serve as biomarkers distinguish cholesterol or pigment gallstone from gallstone subjects, ROC curve analysis was performed using plasma metabolite sets in current study. Metabolite malvidin 3-(6’’-malonylglucoside) which is enriched in cholesterol individuals can be a potential biomarker for cholesterol gallstone (**Figure 6A**, AUC = 0.88). Two plasma metabolites 2-hydroxy-9Z,12Z-Octadecadienoic acid (**Figure 6B**, AUC = 0.981) and DG (18:1(12Z)-2OH(9,10)/i-15:0/0:0), **Figure 6C**, AUC = 0.963) are identified as potential biomarkers of pigment gallstone. These results indicate that the plasma metabolites could be biomarkers for distinguishing different types of gallstones.

### Perturbed gallbladder microbiota–host metabolism interactions are dependent on sample type

**Figure 7:**
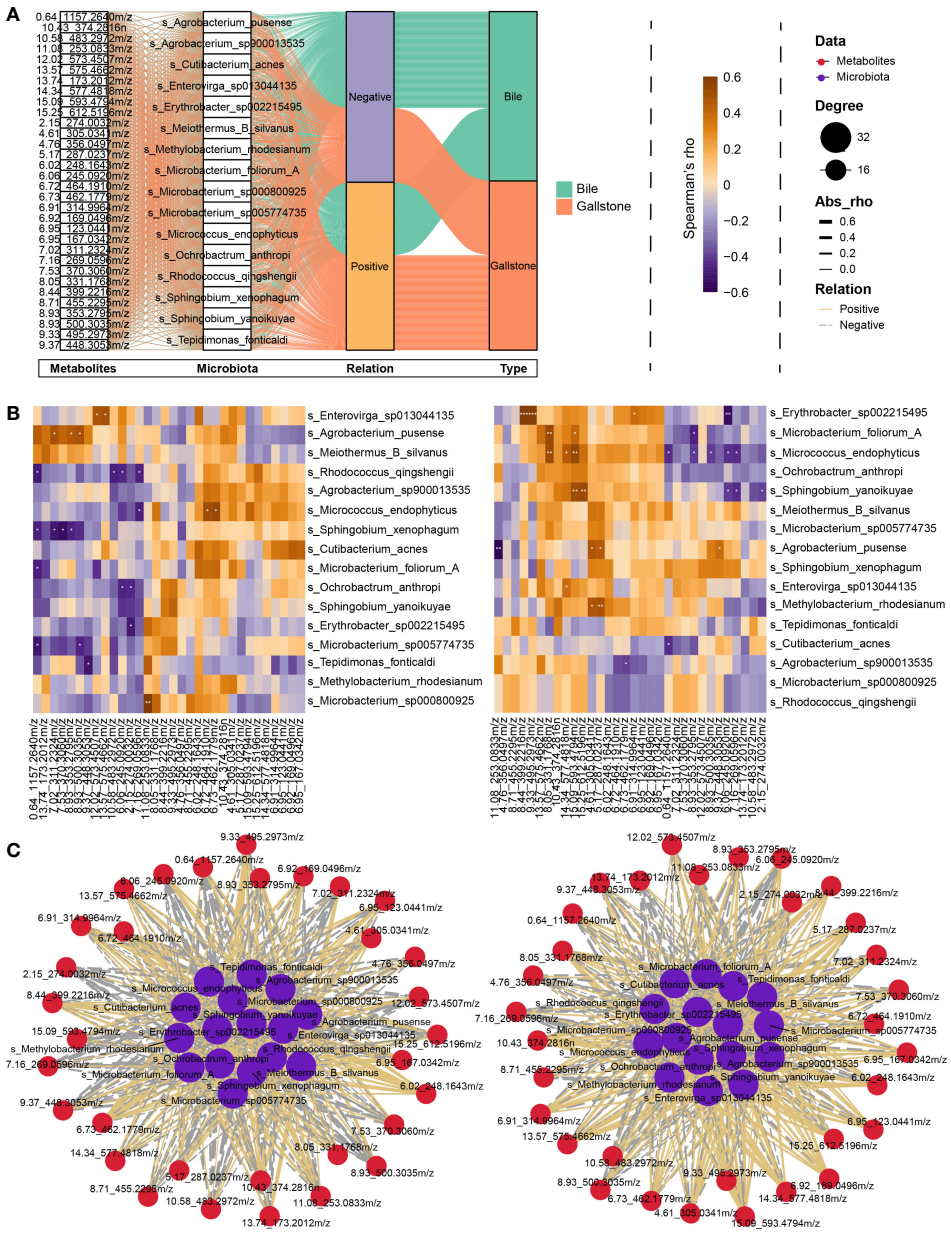
Interplay between gallbladder microbial species and plasma metabolites. **(A)** Sankey diagram showing an overview of the relationship between representative microbiota and metabolites. Significantly altered metabolites, microbial species, relationship, and sample type are shown from left to right. **(B)** Heatmap of the Spearman correlation between representative microbiota and metabolites in bile (Left) and gallstone (Right) related to panel **(A)**. The color indicates the Spearman’s *rho* value. *P < 0.05, **P < 0.01, ***P < 0.001. **(C)** Network of correlation between representative microbiota and metabolites in bile (Left) and gallstone (Right) samples related to panel **(B)**. Node sizes are presented as degrees. The thickness of the edge represents the absolute Spearman’s *rho* value. Solid edges represent positive correlation, and dotted lines represent negative correlation.

A Spearman correlation analysis of representative metabolites (metabolites that altered significantly, related to **Supplementary Table 3**) and microbial species was performed to further probe the relationships between the gallbladder microbiota and plasma metabolites. A total of 1024 connects comprising 508 positive and 516 negative correlations were obtained from the bile and gallstone samples (**Figure 7A**). In bile (**Figures 7B, C** left), *Enterovirga sp013044135* was significantly positively correlated with DG (14:1(9Z)/17:1(9Z)/0:0) [iso2] (12.02_573.4507m/z, *rho* = 0.478, *P* = 0.0328, **Supplementary Table 4**) and vecuronium (13.57_575.4662m/z, *rho* = 0.497, *P* = 0.0257, **Supplementary Table 4**). *Agrobacterium pusense* was significantly positively correlated with pramoxine (7.02_311.2324m/z, *rho* = 0.481, *P* = 0.0333, **Supplementary Table 4**), oxeladin (8.93_353.2795m/z, *rho* = 0.466, *P* = 0.0381, **Supplementary Table 4**), and gentamicin (8.93_500.3035m/z, *rho* = 0.488, *P* = 0.0291, **Supplementary Table 4**). *Micrococcus endophyticus* was significantly positively correlated with dihydroisomorphine-6-glucuronide (6.72_464.1910m/z, *rho* = 0.504, *P* = 0.0236, **Supplementary Table 4**) and 6.73_462.1779m/z (*rho* = 0.532, *P* = 0.0158, **Supplementary Table 4**). *Microbacterium sp000800925* was significantly positively correlated with formyl-5-hydroxykynurenamine (11.08_253.0833m/z, *rho* = 0.608, *P* = 0.0044, **Supplementary Table 4**). *Sphingobium xenophagum* was markedly negatively correlated with N-goshuyoyl lysine (7.53_370.3060m/z, *rho* = -0.550, *P* = 0.0119, **Supplementary Table 4**), pramoxine (7.02_311.2324m/z, *rho* = -0.510, *P* = 0.0216, **Supplementary Table 4**). In gallstone (**Figures 7B, C** right), *Erythrobacter sp002215495* was significantly positively correlated with 5 alpha-Pregnan-3 beta, 20 beta-diol 20-sulfate (8.44_399.2216m/z, *rho* = 0.700, *P* = 0.000596, **Supplementary Table 5**), pregnanediol-3-glucuronide (9.33_495.2973m/z, *rho* = 0.684, *P* = 0.000886, **Supplementary Table 5**), and daidzein 4’-sulfate (6.91_314.9964m/z, *rho* = 0.457, *P* = 0.0430, **Supplementary Table 5**), but markedly negatively correlated with L-cis-Cyclo(aspartylphenylalanyl) (6.06_245.0920m/z, *rho* = -0.632, *P* = 0.00277, **Supplementary Table 5**). The results indicate that the gallbladder microbiota is closely correlated with plasma metabolites.

## Discussion

Mounting evidence exists for the importance of the microbiota in the development of many diseases. However, microbiota alterations in the gallbladder of cholesterol and pigment gallstone subjects remain unexplored. Thus, studies characterizing the gallbladder microbiota in GSD and identifying microbial therapeutic targets at the species level are highly warranted. Accordingly, we use 2bRAD-M sequencing to show for the first time that the gallbladder species-level microbiota is distinct between cholesterol and pigment GSD subjects. These data expand our current insight into the potential role of gallbladder microbiome profiling in GSD.

Little is known about the gallbladder microbiome and its role in cholesterol or pigment GSD. Previously, no studies have described species-level changes in the diversity and structure of the human gallbladder microbiome in cholesterol versus pigment GSD. As we previously reported, the microbiota in bile samples is larger than that in gallstone samples (Hu et al., 2023). The microbiota in cholesterol gallstone exhibited the lowest diversity in current study, consistent with previous findings that cholesterol stones rarely exhibit bacterial signatures (Stewart et al., 1987). The biofilm forming bacterial species *Meiothermus silvanus* that requires sulfur compounds to grow is abundant in the gallbladder, especially in pigment gallstones (Chung et al., 1997). Evidently, Kose et al. found that bacterial carbohydrate and sulfur metabolism were more abundant in pigment gallstones using a metagenomics approach (Kose et al., 2018). Many pathogenic species including *Acinetobacter johnsonii*, *Streptococcus agalactiae*, *Streptococcus mutans*, and *Dietzia maris* are greatly abundant in the gallbladder of GSD subjects (**Supplementary Figure 2**). In addition, *Agrobacterium pusense* (*Rhizobium pusense*), the chief human pathogen within the *Agrobacterium* spp., is also a predominant species in the gallbladder of GSD subjects (Aujoulat et al., 2015). *Vulcaniibacterium thermophilum* can degrade chlorinated phenols and other aromatic compounds and is also one of the most abundant species in the gallbladder (Wu et al., 2021). *Escherichia flexneri* is a waterborne and foodborne bacterial pathogen. Bile salts have been reported to promote strengthened *E. flexneri* biofilm formation and stimulate virulence factor expression (Nickerson et al., 2017). *Cutibacterium acnes* commonly colonizes human skin and, can also colonize medical devices due to its biofilm-forming ability (Ahle et al., 2023). *Klebsiella pneumoniae* typically colonizes human mucosal surfaces of the oropharynx and gastrointestinal tract and can display high degrees of virulence and antibiotic resistance when entering the body (Ashurst and Dawson, 2023). Overall, disordered gallbladder microbial ecology might contribute to the pathogenesis of gallstone and could be a target for controlling pathogenic microbiota and preventing gallstone formation.

Metabolomics is a powerful way to assess the potential role of metabolites on disease. However, the metabolic disorders of people with cholesterol and pigment gallstones remain unclear. Here, we performed untargeted metabolomics of plasma samples to delineate the metabolic variance in plasma samples between cholesterol and pigment GSD individuals. Distinct metabolic profiling was observed between the two gallstone types, indicating that different metabolic dysfunctions might contribute to the formation of different types of gallstones (**Figure 4**). Ergothioneine, synthesized only by some microorganisms, is present at a high level in the plasma of subjects with cholesterol gallstones. However, it is suggested that a deficiency in blood/plasma could be associated with the disease onset or progression (Halliwell et al., 2018). Thus, this finding could potentially be explained by plasma level differences, which were not assessed here and require further study. However, ergothioneine levels are lower in pigment GSD subjects than in cholesterol GSD subjects (**Figure 4**). Plasma metabolites can potentially be assessed to quickly diagnose many diseases, such as Parkinson’s disease and nonalcoholic fatty liver disease (Masoodi et al., 2021; Pathan et al., 2021). Flavonoids, such as malvidin 3-(6’’-malonylglucoside), are closely related to cholesterol gallstones, while 2-hydroxy-9Z,12Z-Octadecadienoic acid is a potential diagnostic marker for pigment gallstones (**Figure 6**). Although we have identified potential plasma metabolite biomarkers, rigorous validation in large cohort is warranted.

The altered metabolite dataset enrichment analysis revealed that bile acid biosynthesis is closely related to cholesterol gallstones (**Figure 5**). Thirty years ago, Berr and coworkers reported that cholesterol GSD is characterized by disordered bile acid metabolism (Berr et al., 1992). A recent study has linked the gut microbiota, bile acids, and biliary cholesterol with the formation of cholesterol gallstones (Hu et al., 2022). Altogether, these evidences suggest that bile acids play a vital role in cholesterol GSD. The detailed changes in the bile acids profiling of cholesterol GSD patients require further investigation using a targeted metabolomics approach.

Accumulating metabolomic evidence suggests that microbiota affect host metabolism. Therefore, we further explored the correlations between gallbladder microbial species and plasma metabolites. We found a strong association between gallbladder microbiota and plasma metabolites. Importantly, the interactions between the microbiota and plasma metabolites differed substantially between bile and gallstone samples (**Figure 7**). Notably, as we reported previously, the gallbladder microbiota of GSD patients differed substantially between bile and gallstone samples (Hu et al., 2023). The current study provides further evidence that the bile and gallstone microbiota pronouncedly differ, and bacteria are the most abundant microbial type in the gallbladder of GSD patients (**Figure 3**; **Supplementary Figure 2**). Thus, even the same species has a different correlation with plasma metabolites between bile and gallstones, such as *Erythrobacter sp002215495* and *Agrobacterium pusense*. This finding indicates that microbiota may differ in different environments and, thus, influence the host metabolism. However, further research is required to elucidate the potential interactions found in the current study.

Moreover, we explore the power for using plasma metabolites to discriminate the two gallstone types. Our results show that some metabolites including malvidin 3-(6’’-malonylglucoside) (AUC = 0.88) are very powerful biomarkers for distinguishing the types of gallstones (**Figure 6**). This, in turn, opens new avenues for metabolite-based diagnostics and even therapeutics that benefit from the easy accessibility of the blood for metabolites monitoring and manipulation. As mentioned above, microbiota can affect host metabolism in both health and disease status by derived microbial compounds (Fan and Pedersen, 2021). Our integrated microbiome and metabolome census analyses also suggest that gallbladder microbiota may affect the plasma metabolites although cause-and-effect study is further needed. Together, our finding implies that plasma metabolite as well as gallbladder microbiota could jointly serve as diagnostic markers for different types of gallstones.

Our study has some limitations. First, this was a cross-sectional study lacking dynamic patient follow-up. Therefore, a longitudinal study on the dynamic evolution of metagenomic and metabolomic profiles in GSD and basic research studies investigating the potential underlying mechanisms are needed. Second, the current study does not involve a control group of healthy subjects. Indeed, as we discussed previously, control bile microbiota could be obtained from liver transplant donors when applicable (Molinero et al., 2019; Hu et al., 2023). Healthy plasma metabolic profiling is also absent. Since this is a cross-sectional study with a wide dispersion of ages, it is difficult to find adequately matched controls. Accordingly, the levels of the thousands of plasma metabolites are strongly influenced by an individual’s genetics, diet, and gut microbial composition (Chen et al., 2022). Moreover, a reference map of potential determinants for the human serum metabolome has also suggested the importance of host genetics, gut microbiome, clinical parameters, diet, and lifestyle (Bar et al., 2020). However, we could not match the details of interested metabolites found in our study from these studies. This phenomenon confirms that the metabolic profile of the current study is a unique, person-specific signature. Thus, the aim of this study was to excavate the alterations and potential applications of gallbladder microbiota and plasma metabolomics between cholesterol and pigment GSD patients, especially focusing on cholesterol GSD. Last, the limited sample size especially in pigment GSD group might limit the significance of the MSEA results. Thus, large cohort concerning this topic is warranted in the future. Regardless, our results shed light on the importance of bile acids metabolism in cholesterol GSD.

## Conclusions

We identified distinct changes in the gallbladder microbiota and functional alterations in bile acid biosynthesis pathways during cholesterol gallstone disease progression. In addition, the intricate interactions among several GSD-related species and the altered plasma metabolites are closely associated with GSD progression. Our multiomics analyses identified potential mechanisms underlying the microbiota–gallbladder–bile acids axis in GSD progression and uncovered promising evidence for novel targets for GSD diagnosis and intervention. Moreover, the sample type (such as bile)-related changes in gallbladder microbiota combined with plasma metabolite differences provide a potential avenue for noninvasively diagnosing gallstone types; however, the findings require validation in a large population.

## Data availability statement

Sequencing data for all samples have been deposited in the Genome Sequence Archive GSA, http://bigd.big.ac.cn/gsa database in the BIG Data Center, Chinese Academy of Sciences, under accession code CRA010801. LCMS metabolomics datasets raw and processed files are available on the EMBL-EBI MetaboLights repository under the MTBLS7736 identifier https://www.ebi.ac.uk/metabolights/MTBLS7736. Other data supporting this study’s findings are available from the corresponding author upon reasonable request. The analysis methods and software used in this article are all open source, and no new methods or algorithms were generated.

## Ethics statement

The studies involving humans were approved by The Institutional Ethics Review Board of the Third People’s Hospital of Chengdu. The studies were conducted in accordance with the local legislation and institutional requirements. Written informed consent for participation in this study was provided by the participants’ legal guardians/next of kin.

## Author contributions

XZ: Formal analysis, Investigation, Writing – original draft, Writing – review & editing. JH: Data curation, Formal analysis, Methodology, Visualization, Writing – original draft, Writing – review & editing. YL: Data curation, Investigation, Resources, Writing – review & editing. JT: Data curation, Investigation, Resources, Writing – review & editing. KY: Data curation, Investigation, Resources, Writing – review & editing. AZ: Investigation, Resources, Writing – review & editing. YJL: Funding acquisition, Investigation, Resources, Supervision, Writing – review & editing. TZ: Conceptualization, Funding acquisition, Project administration, Validation, Writing – review & editing.
