# Supplementary material for: Gallbladder microbial species and host bile acids biosynthesis linked to cholesterol gallstone comparing to pigment individuals
Source: Front Cell Infect Microbiol. 2024 Mar 11;14:1283737. doi: 10.3389/fcimb.2024.1283737 (PMC10962445; doi:10.3389/fcimb.2024.1283737)
Supplement: Supplementary file 1 [file DataSheet_1.docx]

Supplementary Materials

# Supplementary Figures


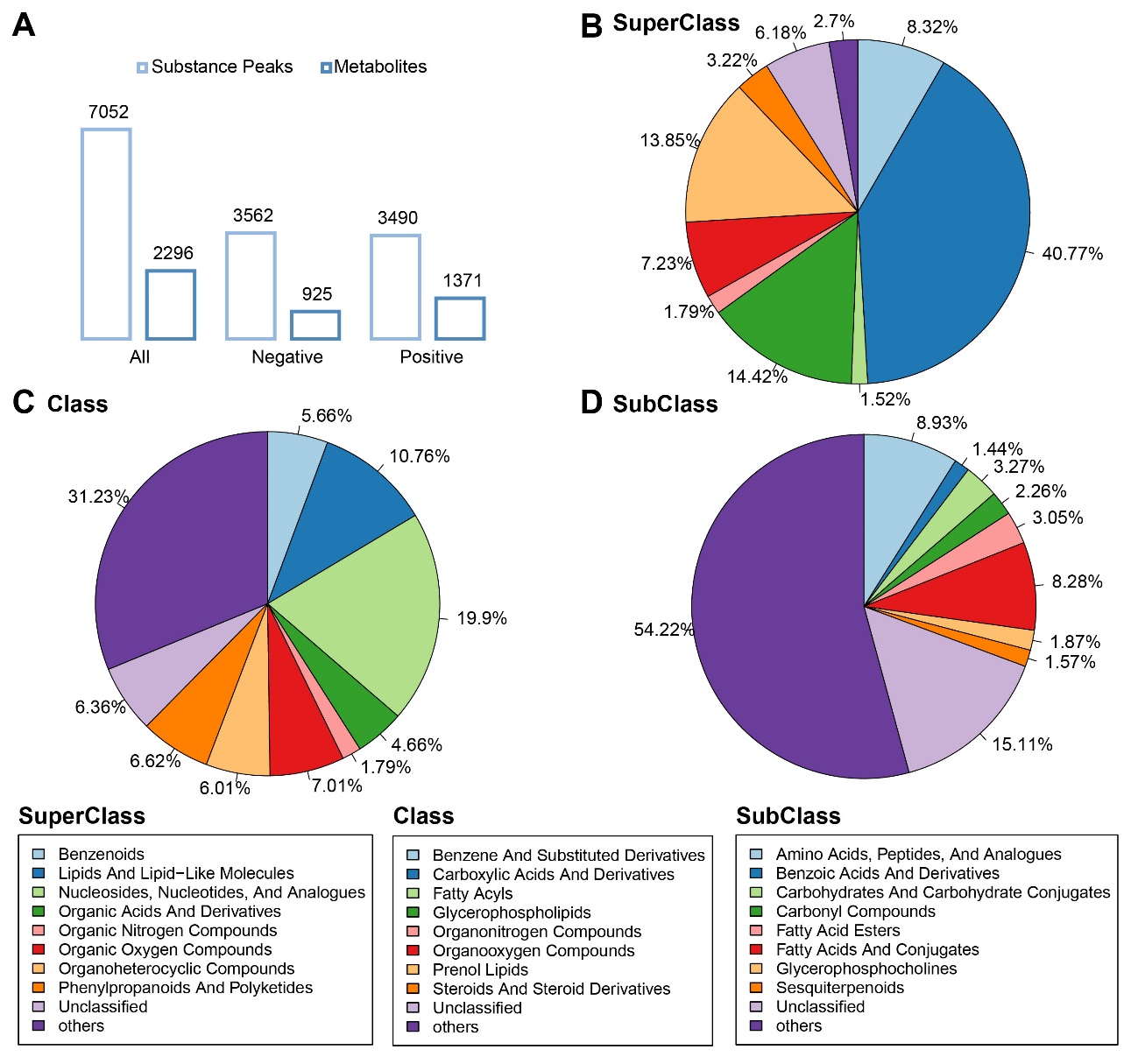


**Figure S1 Overview of detected plasma metabolites using LC-MS untargeted metabolomics.** (**A**) Barplot of detected metabolites comprising both positive and negative compounds. (**B**) Top metabolites at the superclass level. (**C**) Top metabolites at the class level. (**D**) Top metabolites at the subclass level.


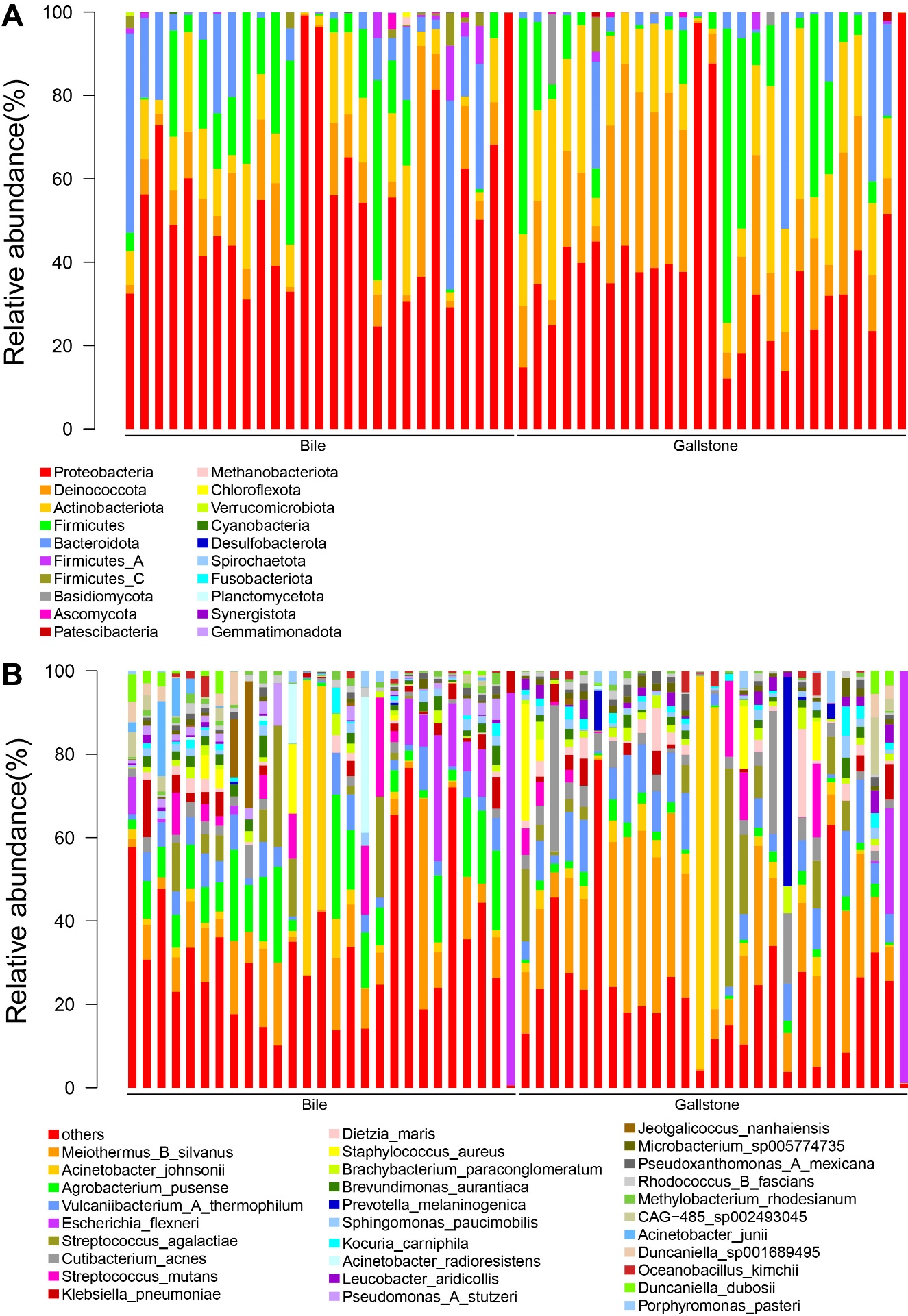


**Figure S2 Gallbladder microbial species composition.** (**A**) Microbial species at the phylum level. (**B**) Microbial species at the species level.


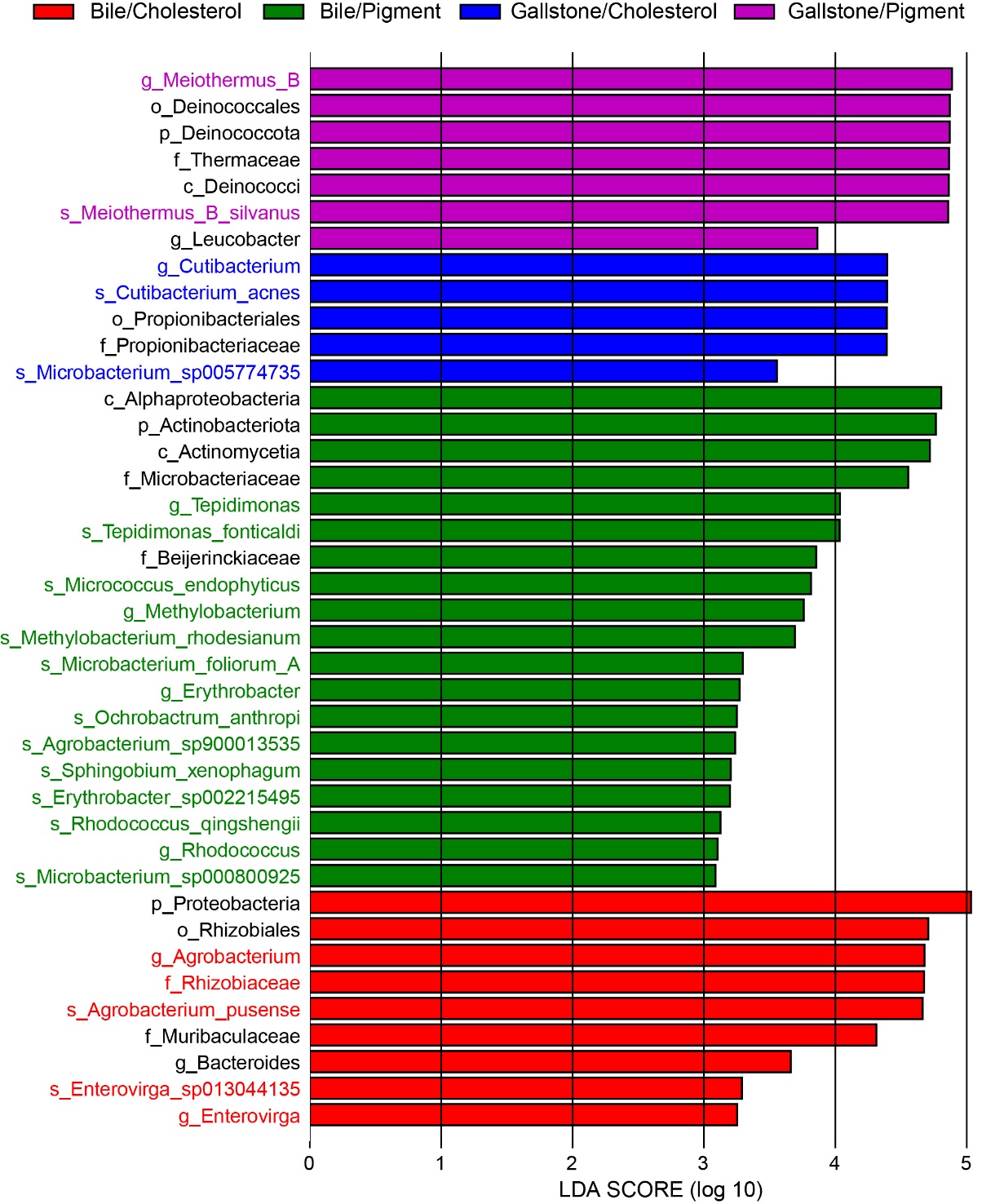


**Figure S3 LEfSe barplot showed the different abundance of gallbladder microbial species (LDA score (log 10) > 3.0)**.


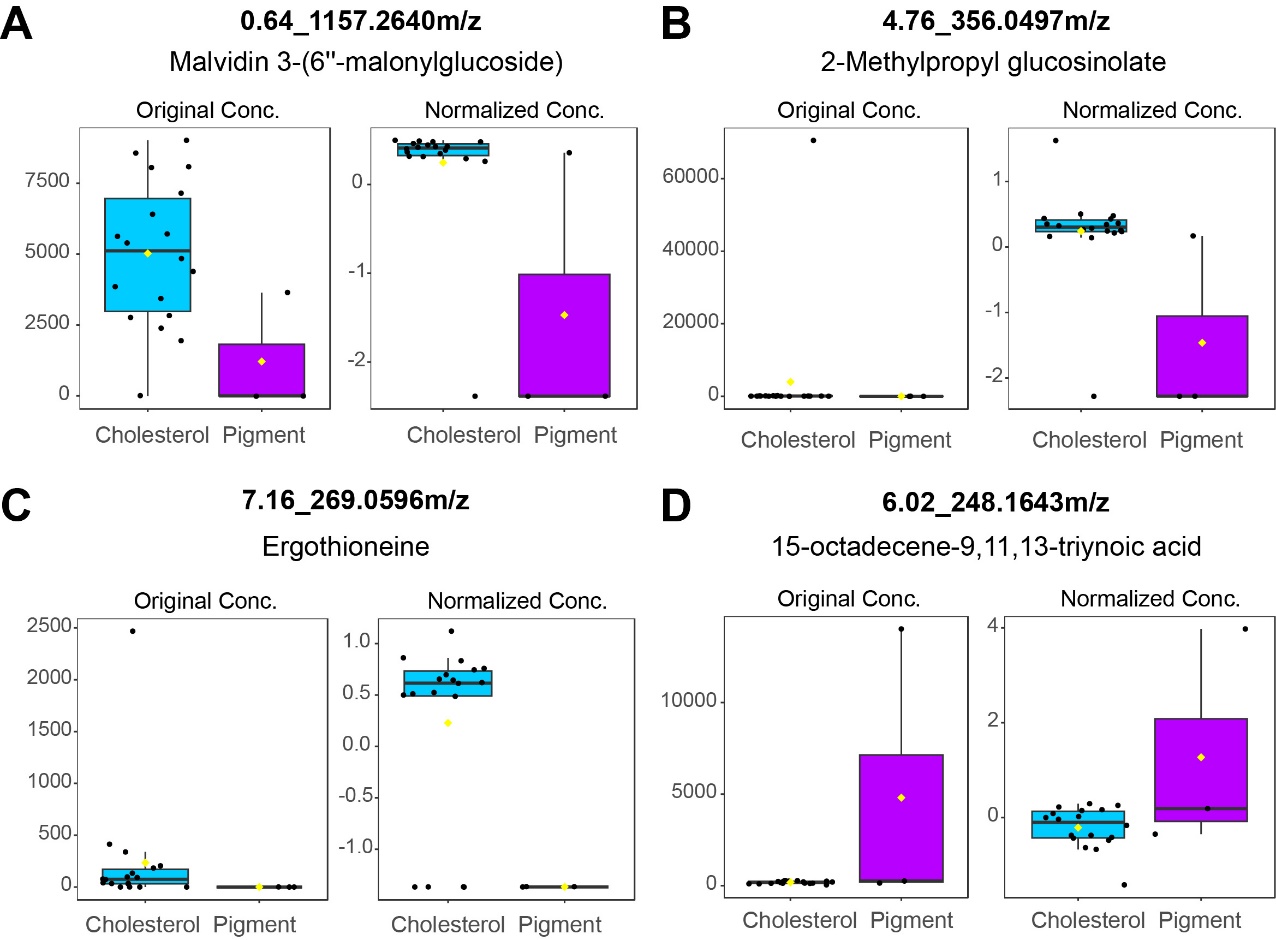


**Figure S4 Boxplot of different metabolites between cholesterol and pigment GSD subjects.** (**A**) Malvidin 3-(6''-malonylglucoside). (**B**) 2-Methylpropyl glucosinolate. (**C**) Ergothioneine. (**D**) 15-octadecene-9,11,13-triynoic acid. Conc.: concentrate.


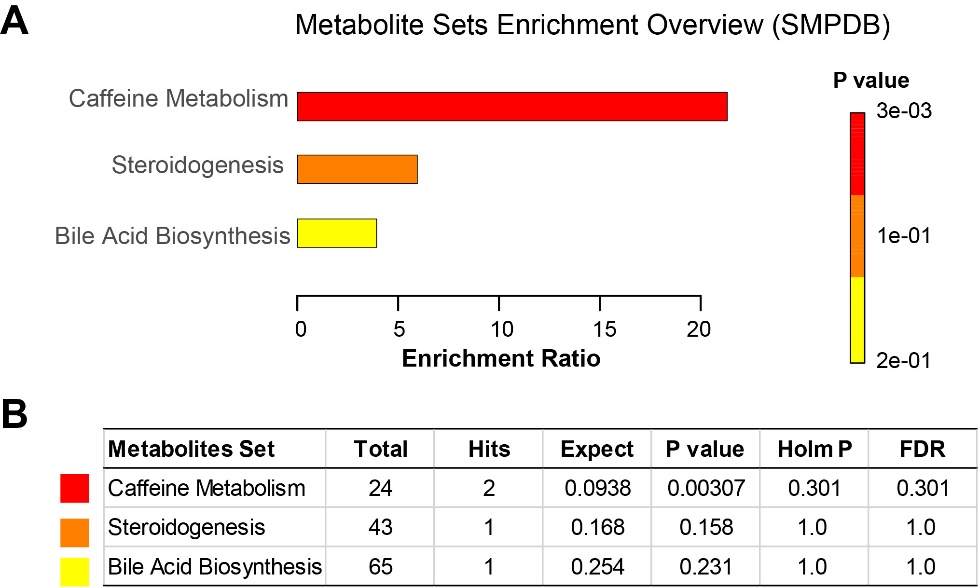


**Figure S5 Metabolite set enrichment analysis (MSEA) of enriched metabolites in pigment GSD subjects**. (**A**) Key pathways related to pigment gallstones. (**B**) Details of key pathways associated with pigment gallstones.

# Supplementary Tables

**Table S1 General information of GSD subjects involved in this study**

| **Subjects** | **Age** | **Sex** | **Type of gallstone** | **Microbial analysis** | **Metabolic analysis** |
| --- | --- | --- | --- | --- | --- |
| Subject 1 | 82 | Female | Cholesterol | Yes | Yes |
| Subject 2 | 25 | Female | Cholesterol | Yes | Yes |
| Subject 3 | 43 | Male | Cholesterol | Yes | Yes |
| Subject 4 | 40 | Male | Pigment | Yes | Yes |
| Subject 5 | 35 | Female | Pigment | Yes | Yes |
| Subject 6 | 35 | Female | Cholesterol | Yes | Yes |
| Subject 7 | 33 | Male | Cholesterol | Yes | Yes |
| Subject 8 | 52 | Female | Cholesterol | Yes | Yes |
| Subject 9 | 48 | Male | Cholesterol | Yes | Yes |
| Subject 10 | 58 | Female | Cholesterol | Yes | Yes |
| Subject 11 | 37 | Female | Cholesterol | Yes | Yes |
| Subject 12 | 23 | Female | Cholesterol | Yes | Yes |
| Subject 13 | 25 | Female | Cholesterol | Yes | No |
| Subject 14 | 46 | Male | Cholesterol | Yes | No |
| Subject 15 | 30 | Female | Cholesterol | Yes | Yes |
| Subject 16 | 50 | Female | Cholesterol | Yes | Yes |
| Subject 17 | 33 | Female | Pigment | Yes | Yes |
| Subject 18 | 50 | Female | Cholesterol | Yes | Yes |
| Subject 19 | 57 | Female | Cholesterol | Yes | Yes |
| Subject 20 | 49 | Female | Cholesterol | Yes | Yes |
| Subject 21 | 53 | Female | Pigment | Yes | No |
| Subject 22 | 65 | Female | Pigment | Yes | No |
| Subject 23 | 29 | Female | Cholesterol | Yes | Yes |
| Subject 24 | 45 | Female | Cholesterol | Yes | Yes |
| Subject 25 | 26 | Male | Cholesterol | Yes | Yes |
| Subject 26 | 70 | Female | Cholesterol | Yes | No |
| Subject 27 | 36 | Male | Cholesterol | Yes | No |
| Subject 28 | 34 | Female | Cholesterol | Yes | No |
| Subject 29 | 21 | Female | Cholesterol | Yes | No |

**Table S2 Adaptors and primers used for 2bRAD-M library preparation**

| **Adaptors** | **Sequence (5’ to 3’)** |
| --- | --- |
| Adap-1 sense | ACACTCTTTCCCTACACGACGCTCTTCCGATCTNNN |
| Adap-1 antisens | AGATCGGAAGAGC (AminoC6) |
| Adap-2 sense | GTGACTGGAGTTCAGACGTGTGCTCTTCCGATCTNNN |
| Adap-2 antisense | AGATCGGAAGAGC (AminoC6) |
| **Primers** |  |
| Primer1 | ACACTCTTTCCCTACACGACGCT |
| Primer2 | GTGACTGGAGTTCAGACGTGTGCT |
| Primer3 | AATGATACGGCGACCACCGAGATCTACACTCTTTCCCTACACGACGCT |
| Index primer | CAAGCAGAAGACGGCATACGAGATXXXXXXGTGACTGGAGTTCAGACGTGT |

**Table S3 Differential metabolites related to volcano plot (Figure 4C)**

| **ID** | **FC** | **log2(FC)** | ***P* val.** | **-LOG10(*P*)** | **Metabolites** |
| --- | --- | --- | --- | --- | --- |
| 6.02_248.1643m/z | 0.038003 | -4.7177 | 0.013046 | 1.8845 | 15-octadecene-9,11,13-triynoic acid |
| 5.17_287.0237m/z | 0.091616 | -3.4482 | 0.043453 | 1.362 | 4-hydroxy-5-[4-hydroxy-3-(sulfooxy)phenyl] pentanoic acid |
| 9.33_495.2973m/z | 0.149 | -2.7466 | 0.024039 | 1.6191 | Pregnanediol-3-glucuronide |
| 4.61_305.0341m/z | 0.18303 | -2.4498 | 0.034738 | 1.4592 | 4-Hydroxy-5-(3',5'-dihydroxyphenyl)-valeric acid sulfate |
| 8.44_399.2216m/z | 0.18435 | -2.4395 | 0.038931 | 1.4097 | 5alpha-Pregnan-3beta,20beta-diol 20-sulfate |
| 6.91_314.9964m/z | 0.31886 | -1.649 | 0.0458 | 1.3391 | Daidzein 4'-sulfate |
| 8.05_331.1768m/z | 0.32276 | -1.6315 | 0.012944 | 1.8879 | Angelicoidenol 2-O-beta-D-glucopyranoside |
| 6.72_464.1910m/z | 0.35228 | -1.5052 | 0.034357 | 1.464 | Dihydroisomorphine-6-glucuronide |
| 6.73_462.1779m/z | 0.36836 | -1.4408 | 0.041574 | 1.3812 | icas#7 |
| 14.34_577.4818m/z | 0.37018 | -1.4337 | 0.013879 | 1.8576 | DG (16:0/15:0/0:0) |
| 6.92_169.0496m/z | 0.39721 | -1.332 | 0.036699 | 1.4353 | Quinolacetic acid |
| 6.95_167.0342m/z | 0.4498 | -1.1526 | 0.023427 | 1.6303 | Dehydroacetic acid |
| 10.43_374.2816n | 0.45072 | -1.1497 | 0.033762 | 1.4716 | 15S-HTPE |
| 15.09_593.4794m/z | 0.45557 | -1.1343 | 0.004739 | 2.3244 | Cohibin A |
| 13.57_575.4662m/z | 0.47424 | -1.0763 | 0.049876 | 1.3021 | Vecuronium |
| 15.25_612.5196m/z | 0.48942 | -1.0308 | 0.007945 | 2.0999 | DG (15:0/0:0/18:1(9Z)-O(12,13)) |
| 6.95_123.0441m/z | 0.49131 | -1.0253 | 0.021238 | 1.6729 | 2-Methylidene-4-(oxiran-2-yl) butanoic Acid |
| 13.74_173.2012m/z | 2.2906 | 1.1958 | 0.024513 | 1.6106 | Propylhexedrine |
| 0.64_1157.2640m/z | 4.1377 | 2.0488 | 0.002979 | 2.526 | Malvidin 3-(6''-malonylglucoside) |
| 6.06_245.0920m/z | 4.2581 | 2.0902 | 0.034141 | 1.4667 | L-cis-Cyclo(aspartylphenylalanyl) |
| 12.02_573.4507m/z | 5.1729 | 2.371 | 0.028107 | 1.5512 | DG (14:1(9Z)/17:1(9Z)/0:0) [iso2] |
| 11.08_253.0833m/z | 5.7152 | 2.5148 | 0.032084 | 1.4937 | Formyl-5-hydroxykynurenamine |
| 9.37_448.3053m/z | 5.8564 | 2.55 | 0.004348 | 2.3617 | 15-keto Latanoprost |
| 7.53_370.3060m/z | 7.5717 | 2.9206 | 0.037085 | 1.4308 | N-goshuyoyl lysine |
| 8.93_500.3035m/z | 16.045 | 4.004 | 0.020109 | 1.6966 | Gentamicin |
| 8.93_353.2795m/z | 146.01 | 7.1899 | 0.028323 | 1.5479 | Oxeladin |
| 4.76_356.0497m/z | 377.22 | 8.5593 | 0.003218 | 2.4924 | 2-Methylpropyl glucosinolate |
| 7.02_311.2324m/z | 474.33 | 8.8897 | 0.019315 | 1.7141 | Pramoxine |
| 7.16_269.0596m/z | 3056200 | 21.543 | 0.006782 | 2.1687 | Ergothioneine |
| 10.58_483.2972m/z | 12206000 | 23.541 | 0.03198 | 1.4951 | 2-Carboxy-4-dodecanolide |
| 8.71_455.2295m/z | 12925000 | 23.624 | 0.032781 | 1.4844 | Isoforskolin |
| 2.15_274.0032m/z | 13575000 | 23.694 | 0.033308 | 1.4774 | 3-Hydroxyhippuric acid sulfate |

**Table S4 Spearman’s correlation between bile microbiota and metabolites**

| **Data1** | **Data2** | ***rho*** | ***P* value** | **Relation** |
| --- | --- | --- | --- | --- |
| 11.08_253.0833m/z | s_Microbacterium_sp000800925 | 0.60830742 | 0.0044294 | positive |
| 7.53_370.3060m/z | s_Sphingobium_xenophagum | -0.55030288 | 0.01193324 | negative |
| 6.73_462.1779m/z | s_Micrococcus_endophyticus | 0.53167374 | 0.0158363 | positive |
| 7.02_311.2324m/z | s_Sphingobium_xenophagum | -0.51013479 | 0.02156085 | negative |
| 9.37_448.3053m/z | s_Tepidimonas_fonticaldi | -0.50593972 | 0.02284634 | negative |
| 6.72_464.1910m/z | s_Micrococcus_endophyticus | 0.50369091 | 0.02356003 | positive |
| 0.64_1157.2640m/z | s_Microbacterium_sp005774735 | -0.50317757 | 0.0237254 | negative |
| 2.15_274.0032m/z | s_Erythrobacter_sp002215495 | -0.4992393 | 0.02502484 | negative |
| 13.57_575.4662m/z | s_Enterovirga_sp013044135 | 0.49736203 | 0.02566372 | positive |
| 0.64_1157.2640m/z | s_Rhodococcus_qingshengii | -0.49291034 | 0.02723025 | negative |
| 8.93_500.3035m/z | s_Agrobacterium_pusense | 0.48795236 | 0.02906257 | positive |
| 7.02_311.2324m/z | s_Agrobacterium_pusense | 0.48120301 | 0.03326706 | positive |
| 12.02_573.4507m/z | s_Enterovirga_sp013044135 | 0.47845928 | 0.03284019 | positive |
| 10.58_483.2972m/z | s_Rhodococcus_qingshengii | -0.47617602 | 0.03380358 | negative |
| 0.64_1157.2640m/z | s_Sphingobium_xenophagum | -0.4738933 | 0.03478866 | negative |
| 0.64_1157.2640m/z | s_Microbacterium_foliorum_A | -0.47052404 | 0.03628334 | negative |
| 7.16_269.0596m/z | s_Micrococcus_endophyticus | -0.46708041 | 0.03786216 | negative |
| 8.93_353.2795m/z | s_Agrobacterium_pusense | 0.46641842 | 0.03817169 | positive |
| 7.16_269.0596m/z | s_Rhodococcus_qingshengii | -0.46124191 | 0.0406603 | negative |
| 8.93_500.3035m/z | s_Microbacterium_sp005774735 | -0.45855326 | 0.04200145 | negative |
| 8.93_353.2795m/z | s_Sphingobium_xenophagum | -0.45561553 | 0.04350564 | negative |
| 6.06_245.0920m/z | s_Ochrobactrum_anthropi | -0.45487149 | 0.04389312 | negative |
| 6.06_245.0920m/z | s_Rhodococcus_qingshengii | -0.45430945 | 0.04418758 | negative |
| 2.15_274.0032m/z | s_Ochrobactrum_anthropi | -0.44847307 | 0.04733606 | negative |
| 11.08_253.0833m/z | s_Tepidimonas_fonticaldi | 0.43742656 | 0.05376358 | positive |
| 8.93_500.3035m/z | s_Sphingobium_xenophagum | -0.43527381 | 0.05509006 | negative |
| 6.92_169.0496m/z | s_Cutibacterium_acnes | 0.43481638 | 0.0553751 | positive |
| 0.64_1157.2640m/z | s_Agrobacterium_pusense | 0.43072338 | 0.05797579 | positive |
| 12.02_573.4507m/z | s_Microbacterium_foliorum_A | -0.42473478 | 0.0619466 | negative |
| 6.06_245.0920m/z | s_Sphingobium_yanoikuyae | -0.4217757 | 0.06398291 | negative |
| 6.02_248.1643m/z | s_Cutibacterium_acnes | 0.41945184 | 0.06561714 | positive |
| 6.06_245.0920m/z | s_Micrococcus_endophyticus | -0.41809638 | 0.06658476 | negative |
| 7.53_370.3060m/z | s_Agrobacterium_pusense | 0.41654135 | 0.06896738 | positive |
| 9.37_448.3053m/z | s_Microbacterium_sp005774735 | -0.41176958 | 0.07124373 | negative |
| 15.09_593.4794m/z | s_Micrococcus_endophyticus | 0.41151218 | 0.07143831 | positive |
| 6.95_167.0342m/z | s_Cutibacterium_acnes | 0.40716022 | 0.07478861 | positive |
| 9.33_495.2973m/z | s_Sphingobium_yanoikuyae | 0.40694402 | 0.07495805 | positive |
| 2.15_274.0032m/z | s_Sphingobium_yanoikuyae | -0.40607178 | 0.07564455 | negative |
| 13.74_173.2012m/z | s_Microbacterium_foliorum_A | -0.40591744 | 0.07576651 | negative |
| 7.53_370.3060m/z | s_Microbacterium_sp005774735 | -0.40408731 | 0.07722388 | negative |
| 6.02_248.1643m/z | s_Microbacterium_foliorum_A | 0.40202371 | 0.07889207 | positive |
| 6.73_462.1779m/z | s_Microbacterium_foliorum_A | 0.40202371 | 0.07889207 | positive |
| 8.05_331.1768m/z | s_Microbacterium_sp000800925 | 0.39834379 | 0.0819331 | positive |
| 15.25_612.5196m/z | s_Rhodococcus_qingshengii | 0.39834379 | 0.0819331 | positive |
| 13.74_173.2012m/z | s_Meiothermus_B_silvanus | 0.39622924 | 0.08371934 | positive |
| 11.08_253.0833m/z | s_Erythrobacter_sp002215495 | 0.39473493 | 0.08499888 | positive |
| 10.58_483.2972m/z | s_Microbacterium_sp005774735 | -0.3944688 | 0.08522828 | negative |
| 0.64_1157.2640m/z | s_Ochrobactrum_anthropi | -0.39181307 | 0.08754246 | negative |
| 6.02_248.1643m/z | s_Enterovirga_sp013044135 | 0.38717257 | 0.09169678 | positive |
| 6.72_464.1910m/z | s_Microbacterium_foliorum_A | 0.38468151 | 0.09398563 | positive |
| 10.58_483.2972m/z | s_Tepidimonas_fonticaldi | -0.37876473 | 0.09958942 | negative |
| 6.91_314.9964m/z | s_Tepidimonas_fonticaldi | -0.37747847 | 0.10083917 | negative |
| 7.16_269.0596m/z | s_Sphingobium_yanoikuyae | -0.37435998 | 0.10391644 | negative |
| 6.92_169.0496m/z | s_Tepidimonas_fonticaldi | -0.37352581 | 0.10475101 | negative |
| 7.02_311.2324m/z | s_Microbacterium_sp005774735 | -0.37335824 | 0.10491925 | negative |
| 6.72_464.1910m/z | s_Agrobacterium_sp900013535 | 0.37275991 | 0.10552155 | positive |
| 13.74_173.2012m/z | s_Sphingobium_yanoikuyae | -0.36940223 | 0.10894807 | negative |
| 10.58_483.2972m/z | s_Agrobacterium_sp900013535 | -0.36519994 | 0.11334886 | negative |
| 13.74_173.2012m/z | s_Agrobacterium_pusense | 0.36377618 | 0.11486848 | positive |
| 13.74_173.2012m/z | s_Sphingobium_xenophagum | -0.36247643 | 0.11626846 | negative |
| 0.64_1157.2640m/z | s_Tepidimonas_fonticaldi | -0.36221294 | 0.11655375 | negative |
| 9.33_495.2973m/z | s_Ochrobactrum_anthropi | 0.361521 | 0.11730535 | positive |
| 6.95_123.0441m/z | s_Cutibacterium_acnes | 0.36106661 | 0.1178008 | positive |
| 4.76_356.0497m/z | s_Sphingobium_xenophagum | -0.36084516 | 0.1180428 | negative |
| 0.64_1157.2640m/z | s_Sphingobium_yanoikuyae | -0.36021014 | 0.11873873 | negative |
| 6.73_462.1779m/z | s_Agrobacterium_sp900013535 | 0.35588931 | 0.1235522 | positive |
| 8.71_455.2295m/z | s_Microbacterium_sp000800925 | 0.35373075 | 0.12600827 | positive |
| 6.73_462.1779m/z | s_Cutibacterium_acnes | 0.35338434 | 0.12640564 | positive |
| 8.93_500.3035m/z | s_Tepidimonas_fonticaldi | -0.35231641 | 0.12763626 | negative |
| 8.05_331.1768m/z | s_Micrococcus_endophyticus | 0.35225443 | 0.12770795 | positive |
| 8.44_399.2216m/z | s_Sphingobium_yanoikuyae | 0.35039827 | 0.12986796 | positive |
| 2.15_274.0032m/z | s_Rhodococcus_qingshengii | -0.3503295 | 0.12994849 | negative |
| 7.16_269.0596m/z | s_Ochrobactrum_anthropi | -0.34702312 | 0.13386176 | negative |
| 6.91_314.9964m/z | s_Cutibacterium_acnes | 0.34570207 | 0.13544833 | positive |
| 7.16_269.0596m/z | s_Methylobacterium_rhodesianum | -0.34441157 | 0.13701097 | negative |
| 2.15_274.0032m/z | s_Micrococcus_endophyticus | -0.34352698 | 0.13808941 | negative |
| 8.93_500.3035m/z | s_Rhodococcus_qingshengii | -0.34124562 | 0.14089827 | negative |
| 9.37_448.3053m/z | s_Microbacterium_sp000800925 | -0.34073209 | 0.14153604 | negative |
| 4.61_305.0341m/z | s_Microbacterium_foliorum_A | 0.33987595 | 0.14260379 | positive |
| 8.93_353.2795m/z | s_Microbacterium_sp005774735 | -0.3377555 | 0.14527263 | negative |
| 4.61_305.0341m/z | s_Methylobacterium_rhodesianum | 0.33436449 | 0.14961291 | positive |
| 10.43_374.2816n | s_Agrobacterium_pusense | 0.33383459 | 0.15038256 | positive |
| 7.16_269.0596m/z | s_Erythrobacter_sp002215495 | -0.33152824 | 0.15331189 | negative |
| 8.44_399.2216m/z | s_Microbacterium_sp005774735 | 0.33033753 | 0.15488355 | positive |
| 9.33_495.2973m/z | s_Microbacterium_sp005774735 | 0.32880108 | 0.15692805 | positive |
| 8.44_399.2216m/z | s_Ochrobactrum_anthropi | 0.32757537 | 0.1585724 | positive |
| 10.58_483.2972m/z | s_Ochrobactrum_anthropi | -0.32616223 | 0.16048292 | negative |
| 9.37_448.3053m/z | s_Agrobacterium_pusense | 0.32481203 | 0.16224603 | positive |
| 5.17_287.0237m/z | s_Microbacterium_sp000800925 | -0.32361107 | 0.16397216 | negative |
| 4.76_356.0497m/z | s_Agrobacterium_pusense | -0.32267772 | 0.16526165 | negative |
| 10.58_483.2972m/z | s_Erythrobacter_sp002215495 | -0.32006696 | 0.16890558 | negative |
| 6.91_314.9964m/z | s_Micrococcus_endophyticus | 0.31933345 | 0.16993919 | positive |
| 10.43_374.2816n | s_Methylobacterium_rhodesianum | 0.31729323 | 0.1726249 | positive |
| 6.72_464.1910m/z | s_Cutibacterium_acnes | 0.31650945 | 0.173959 | positive |
| 4.61_305.0341m/z | s_Enterovirga_sp013044135 | 0.31390648 | 0.17772121 | positive |
| 2.15_274.0032m/z | s_Microbacterium_sp005774735 | -0.31113032 | 0.18179431 | negative |
| 6.95_167.0342m/z | s_Agrobacterium_pusense | -0.30827068 | 0.18568176 | negative |
| 4.61_305.0341m/z | s_Agrobacterium_pusense | 0.30805663 | 0.18637721 | positive |
| 8.05_331.1768m/z | s_Erythrobacter_sp002215495 | 0.30427677 | 0.1921191 | positive |
| 7.02_311.2324m/z | s_Ochrobactrum_anthropi | -0.30296478 | 0.19413958 | negative |
| 2.15_274.0032m/z | s_Agrobacterium_sp900013535 | -0.30294995 | 0.19416251 | negative |
| 7.53_370.3060m/z | s_Erythrobacter_sp002215495 | -0.30270021 | 0.19454875 | negative |
| 4.61_305.0341m/z | s_Micrococcus_endophyticus | 0.30190592 | 0.1957806 | positive |
| 6.95_123.0441m/z | s_Micrococcus_endophyticus | 0.30122692 | 0.19683778 | positive |
| 5.17_287.0237m/z | s_Microbacterium_sp005774735 | -0.30042316 | 0.19809413 | negative |
| 13.74_173.2012m/z | s_Cutibacterium_acnes | -0.29997007 | 0.19880471 | negative |
| 10.43_374.2816n | s_Cutibacterium_acnes | 0.29807201 | 0.20179992 | positive |
| 4.61_305.0341m/z | s_Agrobacterium_sp900013535 | 0.29786684 | 0.20212547 | positive |
| 6.06_245.0920m/z | s_Enterovirga_sp013044135 | 0.29736061 | 0.20293024 | positive |
| 0.64_1157.2640m/z | s_Meiothermus_B_silvanus | 0.29668708 | 0.20400425 | positive |
| 4.76_356.0497m/z | s_Microbacterium_foliorum_A | -0.29492829 | 0.20682665 | negative |
| 10.58_483.2972m/z | s_Micrococcus_endophyticus | -0.29250812 | 0.21075251 | negative |
| 5.17_287.0237m/z | s_Erythrobacter_sp002215495 | -0.28973622 | 0.21530905 | negative |
| 15.25_612.5196m/z | s_Microbacterium_sp000800925 | -0.28970458 | 0.21536145 | negative |
| 10.58_483.2972m/z | s_Sphingobium_xenophagum | -0.28883995 | 0.21679614 | negative |
| 6.02_248.1643m/z | s_Microbacterium_sp000800925 | 0.28805853 | 0.21809817 | positive |
| 15.25_612.5196m/z | s_Agrobacterium_sp900013535 | 0.28599683 | 0.22155802 | positive |
| 9.37_448.3053m/z | s_Erythrobacter_sp002215495 | -0.28535801 | 0.2226373 | negative |
| 6.95_123.0441m/z | s_Agrobacterium_sp900013535 | 0.28278338 | 0.22702196 | positive |
| 7.16_269.0596m/z | s_Microbacterium_sp000800925 | -0.28024825 | 0.23139394 | negative |
| 13.74_173.2012m/z | s_Ochrobactrum_anthropi | -0.27940658 | 0.23285743 | negative |
| 6.06_245.0920m/z | s_Agrobacterium_sp900013535 | -0.27876657 | 0.23397429 | negative |
| 6.92_169.0496m/z | s_Agrobacterium_pusense | -0.27819549 | 0.23408177 | negative |
| 6.72_464.1910m/z | s_Rhodococcus_qingshengii | 0.27818223 | 0.23499702 | positive |
| 6.06_245.0920m/z | s_Microbacterium_sp000800925 | -0.27818223 | 0.23499702 | negative |
| 8.93_500.3035m/z | s_Microbacterium_sp000800925 | -0.27365591 | 0.24301705 | negative |
| 13.74_173.2012m/z | s_Rhodococcus_qingshengii | -0.27262488 | 0.24486817 | negative |
| 6.72_464.1910m/z | s_Microbacterium_sp000800925 | 0.27159804 | 0.24672072 | positive |
| 12.02_573.4507m/z | s_Rhodococcus_qingshengii | -0.27035885 | 0.24896829 | negative |
| 6.06_245.0920m/z | s_Erythrobacter_sp002215495 | -0.26959237 | 0.25036501 | negative |
| 2.15_274.0032m/z | s_Methylobacterium_rhodesianum | -0.26955329 | 0.25043637 | negative |
| 15.09_593.4794m/z | s_Agrobacterium_pusense | -0.26917293 | 0.25009701 | negative |
| 15.25_612.5196m/z | s_Micrococcus_endophyticus | 0.26830594 | 0.25272044 | positive |
| 6.02_248.1643m/z | s_Methylobacterium_rhodesianum | 0.26616541 | 0.25559087 | positive |
| 7.02_311.2324m/z | s_Meiothermus_B_silvanus | 0.26466165 | 0.25836703 | positive |
| 6.06_245.0920m/z | s_Agrobacterium_pusense | 0.26466165 | 0.25836703 | positive |
| 6.95_123.0441m/z | s_Agrobacterium_pusense | -0.26466165 | 0.25836703 | negative |
| 15.09_593.4794m/z | s_Rhodococcus_qingshengii | 0.2633678 | 0.26189266 | positive |
| 9.33_495.2973m/z | s_Agrobacterium_pusense | -0.2631579 | 0.26116269 | negative |
| 4.76_356.0497m/z | s_Agrobacterium_sp900013535 | -0.26279815 | 0.26296407 | negative |
| 4.76_356.0497m/z | s_Methylobacterium_rhodesianum | -0.26250472 | 0.26351704 | negative |
| 4.76_356.0497m/z | s_Enterovirga_sp013044135 | -0.26198717 | 0.26449416 | negative |
| 4.76_356.0497m/z | s_Rhodococcus_qingshengii | -0.26182019 | 0.26480988 | negative |
| 14.34_577.4818m/z | s_Microbacterium_sp000800925 | -0.26172175 | 0.26499614 | negative |
| 13.74_173.2012m/z | s_Microbacterium_sp005774735 | -0.26064237 | 0.26704372 | negative |
| 6.02_248.1643m/z | s_Micrococcus_endophyticus | 0.2600757 | 0.26812268 | positive |
| 13.57_575.4662m/z | s_Cutibacterium_acnes | 0.25966067 | 0.26891464 | positive |
| 11.08_253.0833m/z | s_Enterovirga_sp013044135 | -0.25925993 | 0.26968072 | negative |
| 12.02_573.4507m/z | s_Sphingobium_xenophagum | -0.2590724 | 0.27003967 | negative |
| 9.37_448.3053m/z | s_Micrococcus_endophyticus | -0.25842965 | 0.27127227 | negative |
| 8.44_399.2216m/z | s_Erythrobacter_sp002215495 | 0.25697986 | 0.27406542 | positive |
| 10.58_483.2972m/z | s_Microbacterium_foliorum_A | -0.2557278 | 0.27649203 | negative |
| 6.73_462.1779m/z | s_Rhodococcus_qingshengii | 0.25513755 | 0.27764061 | positive |
| 10.58_483.2972m/z | s_Sphingobium_yanoikuyae | -0.25379486 | 0.28026443 | negative |
| 2.15_274.0032m/z | s_Microbacterium_sp000800925 | -0.25254335 | 0.28272388 | negative |
| 0.64_1157.2640m/z | s_Agrobacterium_sp900013535 | -0.24941753 | 0.28892487 | negative |
| 15.09_593.4794m/z | s_Meiothermus_B_silvanus | -0.2481203 | 0.29019433 | negative |
| 9.33_495.2973m/z | s_Erythrobacter_sp002215495 | 0.24752048 | 0.29272871 | positive |
| 5.17_287.0237m/z | s_Agrobacterium_sp900013535 | 0.2472103 | 0.29335358 | positive |
| 14.34_577.4818m/z | s_Meiothermus_B_silvanus | -0.24661654 | 0.29320511 | negative |
| 8.93_353.2795m/z | s_Meiothermus_B_silvanus | 0.24603949 | 0.29571956 | positive |
| 8.05_331.1768m/z | s_Microbacterium_sp005774735 | 0.24583258 | 0.29613889 | positive |
| 6.95_167.0342m/z | s_Tepidimonas_fonticaldi | -0.24506455 | 0.29769859 | negative |
| 10.43_374.2816n | s_Agrobacterium_sp900013535 | 0.24422201 | 0.29941538 | positive |
| 15.09_593.4794m/z | s_Microbacterium_sp000800925 | -0.24196916 | 0.30403539 | negative |
| 15.09_593.4794m/z | s_Enterovirga_sp013044135 | -0.24151115 | 0.30497991 | negative |
| 7.02_311.2324m/z | s_Rhodococcus_qingshengii | -0.24032311 | 0.30743821 | negative |
| 6.02_248.1643m/z | s_Tepidimonas_fonticaldi | 0.23715925 | 0.31404315 | positive |
| 14.34_577.4818m/z | s_Agrobacterium_sp900013535 | 0.23538503 | 0.31778404 | positive |
| 7.16_269.0596m/z | s_Cutibacterium_acnes | -0.23356172 | 0.32165613 | negative |
| 7.16_269.0596m/z | s_Agrobacterium_sp900013535 | -0.23325275 | 0.32231503 | negative |
| 2.15_274.0032m/z | s_Tepidimonas_fonticaldi | -0.23277185 | 0.32334222 | negative |
| 6.91_314.9964m/z | s_Agrobacterium_sp900013535 | 0.23217158 | 0.32462711 | positive |
| 6.92_169.0496m/z | s_Micrococcus_endophyticus | 0.23209287 | 0.32479582 | positive |
| 6.73_462.1779m/z | s_Tepidimonas_fonticaldi | -0.23123026 | 0.32664812 | negative |
| 6.72_464.1910m/z | s_Agrobacterium_pusense | 0.23007519 | 0.32761354 | positive |
| 6.02_248.1643m/z | s_Agrobacterium_sp900013535 | 0.22895813 | 0.33155706 | positive |
| 13.74_173.2012m/z | s_Methylobacterium_rhodesianum | 0.22868087 | 0.33215905 | positive |
| 6.91_314.9964m/z | s_Agrobacterium_pusense | -0.22857143 | 0.33085852 | negative |
| 5.17_287.0237m/z | s_Enterovirga_sp013044135 | 0.22821097 | 0.33318079 | positive |
| 8.93_353.2795m/z | s_Microbacterium_sp000800925 | -0.22553513 | 0.33903422 | negative |
| 8.71_455.2295m/z | s_Cutibacterium_acnes | 0.22541074 | 0.33930778 | positive |
| 13.57_575.4662m/z | s_Ochrobactrum_anthropi | -0.22404118 | 0.34232829 | negative |
| 7.53_370.3060m/z | s_Ochrobactrum_anthropi | -0.22319254 | 0.3442078 | negative |
| 15.09_593.4794m/z | s_Microbacterium_foliorum_A | -0.22229547 | 0.3462011 | negative |
| 14.34_577.4818m/z | s_Cutibacterium_acnes | 0.22124932 | 0.34853409 | positive |
| 8.93_500.3035m/z | s_Erythrobacter_sp002215495 | -0.21947262 | 0.35251716 | negative |
| 12.02_573.4507m/z | s_Microbacterium_sp000800925 | -0.21925443 | 0.3530081 | negative |
| 8.71_455.2295m/z | s_Agrobacterium_sp900013535 | -0.21745996 | 0.35706083 | negative |
| 9.37_448.3053m/z | s_Sphingobium_xenophagum | -0.21690771 | 0.35831344 | negative |
| 15.25_612.5196m/z | s_Agrobacterium_pusense | -0.21654135 | 0.35751529 | negative |
| 6.06_245.0920m/z | s_Meiothermus_B_silvanus | 0.21654135 | 0.35751529 | positive |
| 12.02_573.4507m/z | s_Ochrobactrum_anthropi | -0.21587965 | 0.36065196 | negative |
| 12.02_573.4507m/z | s_Tepidimonas_fonticaldi | -0.21574432 | 0.36096045 | negative |
| 13.74_173.2012m/z | s_Agrobacterium_sp900013535 | -0.21571178 | 0.36103465 | negative |
| 6.73_462.1779m/z | s_Microbacterium_sp000800925 | 0.21563238 | 0.36121572 | positive |
| 6.95_123.0441m/z | s_Tepidimonas_fonticaldi | -0.21541965 | 0.36170116 | negative |
| 8.93_500.3035m/z | s_Meiothermus_B_silvanus | 0.21536169 | 0.36183347 | positive |
| 7.16_269.0596m/z | s_Microbacterium_sp005774735 | -0.21487678 | 0.3629416 | negative |
| 12.02_573.4507m/z | s_Micrococcus_endophyticus | -0.21430885 | 0.36424192 | negative |
| 5.17_287.0237m/z | s_Microbacterium_foliorum_A | 0.21309089 | 0.36703947 | positive |
| 10.58_483.2972m/z | s_Methylobacterium_rhodesianum | -0.21284611 | 0.36760316 | negative |
| 7.53_370.3060m/z | s_Rhodococcus_qingshengii | -0.21234029 | 0.36876961 | negative |
| 8.44_399.2216m/z | s_Agrobacterium_pusense | -0.21052632 | 0.37130519 | negative |
| 6.72_464.1910m/z | s_Methylobacterium_rhodesianum | 0.21052632 | 0.37130519 | positive |
| 4.61_305.0341m/z | s_Meiothermus_B_silvanus | 0.20961429 | 0.37509192 | positive |
| 6.73_462.1779m/z | s_Sphingobium_yanoikuyae | 0.2085704 | 0.37752908 | positive |
| 8.44_399.2216m/z | s_Cutibacterium_acnes | 0.20742124 | 0.38022229 | positive |
| 11.08_253.0833m/z | s_Agrobacterium_sp900013535 | -0.20677518 | 0.38174116 | negative |
| 10.43_374.2816n | s_Micrococcus_endophyticus | 0.20575609 | 0.38414388 | positive |
| 0.64_1157.2640m/z | s_Micrococcus_endophyticus | -0.20112061 | 0.39517922 | negative |
| 14.34_577.4818m/z | s_Micrococcus_endophyticus | 0.1991719 | 0.39987009 | positive |
| 8.93_500.3035m/z | s_Ochrobactrum_anthropi | -0.19803133 | 0.40262973 | negative |
| 6.73_462.1779m/z | s_Agrobacterium_pusense | 0.19699248 | 0.4034417 | positive |
| 6.73_462.1779m/z | s_Ochrobactrum_anthropi | 0.19688468 | 0.4054146 | positive |
| 9.37_448.3053m/z | s_Rhodococcus_qingshengii | -0.19423375 | 0.41189297 | negative |
| 8.93_353.2795m/z | s_Erythrobacter_sp002215495 | -0.19306795 | 0.41475963 | negative |
| 8.93_500.3035m/z | s_Micrococcus_endophyticus | -0.19287796 | 0.41522782 | negative |
| 7.53_370.3060m/z | s_Microbacterium_sp000800925 | -0.1925877 | 0.41594366 | negative |
| 6.72_464.1910m/z | s_Tepidimonas_fonticaldi | -0.1897274 | 0.4230333 | negative |
| 14.34_577.4818m/z | s_Agrobacterium_pusense | -0.18947368 | 0.42194872 | negative |
| 6.72_464.1910m/z | s_Ochrobactrum_anthropi | 0.18924691 | 0.42423055 | positive |
| 7.02_311.2324m/z | s_Erythrobacter_sp002215495 | -0.18918763 | 0.42437838 | negative |
| 15.25_612.5196m/z | s_Sphingobium_xenophagum | 0.18798668 | 0.42737932 | positive |
| 10.43_374.2816n | s_Meiothermus_B_silvanus | 0.18796993 | 0.42570529 | positive |
| 11.08_253.0833m/z | s_Microbacterium_sp005774735 | 0.18772986 | 0.42802252 | positive |
| 6.95_167.0342m/z | s_Micrococcus_endophyticus | 0.18764955 | 0.42822374 | positive |
| 6.95_123.0441m/z | s_Rhodococcus_qingshengii | 0.18764955 | 0.42822374 | positive |
| 6.72_464.1910m/z | s_Sphingobium_yanoikuyae | 0.18724987 | 0.42922603 | positive |
| 10.43_374.2816n | s_Microbacterium_sp000800925 | 0.18600351 | 0.43235949 | positive |
| 6.95_123.0441m/z | s_Ochrobactrum_anthropi | 0.18585235 | 0.43274034 | positive |
| 5.17_287.0237m/z | s_Tepidimonas_fonticaldi | -0.18582509 | 0.43280904 | negative |
| 0.64_1157.2640m/z | s_Erythrobacter_sp002215495 | -0.18473595 | 0.43555861 | negative |
| 10.43_374.2816n | s_Enterovirga_sp013044135 | 0.18339753 | 0.43895005 | positive |
| 8.71_455.2295m/z | s_Methylobacterium_rhodesianum | -0.18177369 | 0.44308329 | negative |
| 6.06_245.0920m/z | s_Microbacterium_sp005774735 | -0.18130153 | 0.44428889 | negative |
| 8.05_331.1768m/z | s_Methylobacterium_rhodesianum | 0.18045113 | 0.44476034 | positive |
| 8.93_500.3035m/z | s_Microbacterium_foliorum_A | -0.17999913 | 0.44762329 | negative |
| 15.09_593.4794m/z | s_Erythrobacter_sp002215495 | -0.17972825 | 0.44831842 | negative |
| 8.44_399.2216m/z | s_Micrococcus_endophyticus | -0.17777326 | 0.45335187 | negative |
| 5.17_287.0237m/z | s_Micrococcus_endophyticus | 0.17675496 | 0.45598517 | positive |
| 10.43_374.2816n | s_Erythrobacter_sp002215495 | 0.17657512 | 0.45645104 | positive |
| 15.09_593.4794m/z | s_Tepidimonas_fonticaldi | -0.17589311 | 0.45822001 | negative |
| 8.71_455.2295m/z | s_Enterovirga_sp013044135 | -0.17583307 | 0.4583759 | negative |
| 6.92_169.0496m/z | s_Agrobacterium_sp900013535 | 0.17432953 | 0.46228877 | positive |
| 6.91_314.9964m/z | s_Ochrobactrum_anthropi | 0.17312273 | 0.46544165 | positive |
| 6.02_248.1643m/z | s_Agrobacterium_pusense | 0.17293233 | 0.4642626 | positive |
| 8.71_455.2295m/z | s_Tepidimonas_fonticaldi | -0.17253703 | 0.46697578 | negative |
| 8.93_500.3035m/z | s_Cutibacterium_acnes | -0.17234216 | 0.46748676 | negative |
| 6.72_464.1910m/z | s_Meiothermus_B_silvanus | 0.17142857 | 0.46821588 | positive |
| 6.73_462.1779m/z | s_Methylobacterium_rhodesianum | 0.17142857 | 0.46821588 | positive |
| 7.02_311.2324m/z | s_Micrococcus_endophyticus | -0.17118907 | 0.47051622 | negative |
| 13.74_173.2012m/z | s_Enterovirga_sp013044135 | -0.16893939 | 0.47645503 | negative |
| 4.76_356.0497m/z | s_Micrococcus_endophyticus | -0.16878346 | 0.47686807 | negative |
| 15.25_612.5196m/z | s_Meiothermus_B_silvanus | -0.16842105 | 0.47617463 | negative |
| 10.43_374.2816n | s_Rhodococcus_qingshengii | 0.16789697 | 0.47921954 | positive |
| 14.34_577.4818m/z | s_Rhodococcus_qingshengii | 0.16789697 | 0.47921954 | positive |
| 11.08_253.0833m/z | s_Microbacterium_foliorum_A | -0.16420973 | 0.48906192 | negative |
| 14.34_577.4818m/z | s_Erythrobacter_sp002215495 | -0.16396261 | 0.48972509 | negative |
| 6.95_167.0342m/z | s_Meiothermus_B_silvanus | -0.16390977 | 0.48824191 | negative |
| 9.37_448.3053m/z | s_Meiothermus_B_silvanus | 0.16240602 | 0.49229843 | positive |
| 6.95_123.0441m/z | s_Methylobacterium_rhodesianum | -0.16240602 | 0.49229843 | negative |
| 4.61_305.0341m/z | s_Microbacterium_sp005774735 | -0.16127906 | 0.49695509 | negative |
| 8.05_331.1768m/z | s_Sphingobium_xenophagum | 0.15986901 | 0.50077473 | positive |
| 6.95_123.0441m/z | s_Meiothermus_B_silvanus | -0.1593985 | 0.50046206 | negative |
| 4.61_305.0341m/z | s_Cutibacterium_acnes | 0.1569436 | 0.5087444 | positive |
| 9.37_448.3053m/z | s_Microbacterium_foliorum_A | -0.1560798 | 0.51110921 | negative |
| 7.16_269.0596m/z | s_Tepidimonas_fonticaldi | -0.15522135 | 0.51346453 | negative |
| 9.33_495.2973m/z | s_Microbacterium_sp000800925 | 0.15472858 | 0.51481887 | positive |
| 15.25_612.5196m/z | s_Enterovirga_sp013044135 | -0.15320864 | 0.51900698 | negative |
| 10.43_374.2816n | s_Sphingobium_yanoikuyae | 0.15295163 | 0.51971675 | positive |
| 7.53_370.3060m/z | s_Methylobacterium_rhodesianum | -0.1518797 | 0.52116176 | negative |
| 12.02_573.4507m/z | s_Erythrobacter_sp002215495 | -0.15157821 | 0.52351731 | negative |
| 6.95_167.0342m/z | s_Microbacterium_foliorum_A | 0.1513501 | 0.5241498 | positive |
| 14.34_577.4818m/z | s_Enterovirga_sp013044135 | 0.15094447 | 0.52527541 | positive |
| 2.15_274.0032m/z | s_Enterovirga_sp013044135 | 0.15088115 | 0.52545123 | positive |
| 10.43_374.2816n | s_Tepidimonas_fonticaldi | -0.15020086 | 0.52734183 | negative |
| 4.61_305.0341m/z | s_Tepidimonas_fonticaldi | -0.14945457 | 0.52941948 | negative |
| 4.76_356.0497m/z | s_Tepidimonas_fonticaldi | -0.14828028 | 0.53269633 | negative |
| 4.76_356.0497m/z | s_Ochrobactrum_anthropi | 0.14814353 | 0.53307855 | positive |
| 9.37_448.3053m/z | s_Methylobacterium_rhodesianum | -0.14736842 | 0.53377687 | negative |
| 7.02_311.2324m/z | s_Enterovirga_sp013044135 | -0.14717086 | 0.53580078 | negative |
| 8.93_353.2795m/z | s_Ochrobactrum_anthropi | -0.14694401 | 0.53643659 | negative |
| 15.09_593.4794m/z | s_Ochrobactrum_anthropi | -0.14596623 | 0.53918106 | negative |
| 7.02_311.2324m/z | s_Methylobacterium_rhodesianum | -0.14586466 | 0.53801382 | negative |
| 13.74_173.2012m/z | s_Tepidimonas_fonticaldi | -0.14580923 | 0.53962232 | negative |
| 6.92_169.0496m/z | s_Sphingobium_xenophagum | 0.14380178 | 0.54527904 | positive |
| 13.57_575.4662m/z | s_Rhodococcus_qingshengii | -0.14320624 | 0.54696233 | negative |
| 15.09_593.4794m/z | s_Agrobacterium_sp900013535 | 0.14219505 | 0.54982584 | positive |
| 6.95_123.0441m/z | s_Erythrobacter_sp002215495 | -0.14189072 | 0.55068897 | negative |
| 7.02_311.2324m/z | s_Cutibacterium_acnes | -0.14135374 | 0.55221344 | negative |
| 4.61_305.0341m/z | s_Microbacterium_sp000800925 | -0.14119907 | 0.55265286 | negative |
| 8.05_331.1768m/z | s_Microbacterium_foliorum_A | 0.14031416 | 0.55517009 | positive |
| 6.02_248.1643m/z | s_Erythrobacter_sp002215495 | 0.14031416 | 0.55517009 | positive |
| 6.02_248.1643m/z | s_Microbacterium_sp005774735 | 0.13981728 | 0.55658575 | positive |
| 6.95_167.0342m/z | s_Erythrobacter_sp002215495 | -0.1387376 | 0.55966742 | negative |
| 7.53_370.3060m/z | s_Meiothermus_B_silvanus | 0.13834587 | 0.55943308 | positive |
| 8.05_331.1768m/z | s_Meiothermus_B_silvanus | 0.13834587 | 0.55943308 | positive |
| 5.17_287.0237m/z | s_Sphingobium_yanoikuyae | -0.13767269 | 0.56271428 | negative |
| 8.71_455.2295m/z | s_Microbacterium_sp005774735 | -0.13731006 | 0.56375349 | negative |
| 6.95_167.0342m/z | s_Methylobacterium_rhodesianum | -0.13684211 | 0.56376303 | negative |
| 8.93_353.2795m/z | s_Rhodococcus_qingshengii | -0.13631244 | 0.56661679 | negative |
| 5.17_287.0237m/z | s_Ochrobactrum_anthropi | -0.13555888 | 0.56878381 | negative |
| 15.09_593.4794m/z | s_Sphingobium_yanoikuyae | -0.13533901 | 0.56941677 | negative |
| 6.73_462.1779m/z | s_Meiothermus_B_silvanus | 0.13533835 | 0.56810808 | positive |
| 4.76_356.0497m/z | s_Cutibacterium_acnes | -0.13525878 | 0.56964783 | negative |
| 15.25_612.5196m/z | s_Erythrobacter_sp002215495 | -0.1340079 | 0.57325533 | negative |
| 8.44_399.2216m/z | s_Methylobacterium_rhodesianum | -0.13383459 | 0.5724681 | negative |
| 6.95_167.0342m/z | s_Agrobacterium_sp900013535 | 0.13335807 | 0.57513333 | positive |
| 7.02_311.2324m/z | s_Microbacterium_sp000800925 | -0.13003785 | 0.58476992 | negative |
| 9.33_495.2973m/z | s_Rhodococcus_qingshengii | 0.13003785 | 0.58476992 | positive |
| 13.57_575.4662m/z | s_Microbacterium_sp005774735 | 0.12906211 | 0.58761488 | positive |
| 6.92_169.0496m/z | s_Ochrobactrum_anthropi | 0.1247502 | 0.60025647 | positive |
| 7.16_269.0596m/z | s_Microbacterium_foliorum_A | -0.12462266 | 0.6006321 | negative |
| 8.44_399.2216m/z | s_Enterovirga_sp013044135 | 0.12377447 | 0.60313264 | positive |
| 6.92_169.0496m/z | s_Methylobacterium_rhodesianum | -0.12330827 | 0.6033973 | negative |
| 9.33_495.2973m/z | s_Enterovirga_sp013044135 | -0.12226502 | 0.60759312 | negative |
| 8.93_353.2795m/z | s_Micrococcus_endophyticus | -0.11978972 | 0.61493668 | negative |
| 6.72_464.1910m/z | s_Sphingobium_xenophagum | 0.11729083 | 0.62238615 | positive |
| 6.95_123.0441m/z | s_Microbacterium_foliorum_A | 0.11666571 | 0.62425531 | positive |
| 8.05_331.1768m/z | s_Enterovirga_sp013044135 | -0.11547252 | 0.62782911 | negative |
| 12.02_573.4507m/z | s_Microbacterium_sp005774735 | -0.1154077 | 0.62802349 | negative |
| 6.95_167.0342m/z | s_Microbacterium_sp000800925 | -0.11522341 | 0.62857625 | negative |
| 8.93_500.3035m/z | s_Sphingobium_yanoikuyae | -0.11511871 | 0.62889039 | negative |
| 6.72_464.1910m/z | s_Enterovirga_sp013044135 | -0.1147178 | 0.63009377 | negative |
| 15.25_612.5196m/z | s_Cutibacterium_acnes | -0.11369757 | 0.63316017 | negative |
| 15.25_612.5196m/z | s_Microbacterium_foliorum_A | -0.11351258 | 0.6337168 | negative |
| 6.73_462.1779m/z | s_Enterovirga_sp013044135 | -0.11320835 | 0.63463261 | negative |
| 7.53_370.3060m/z | s_Cutibacterium_acnes | -0.11216112 | 0.63778902 | negative |
| 13.57_575.4662m/z | s_Microbacterium_sp000800925 | -0.11193131 | 0.63848246 | negative |
| 6.02_248.1643m/z | s_Sphingobium_yanoikuyae | 0.11123755 | 0.64057768 | positive |
| 2.15_274.0032m/z | s_Meiothermus_B_silvanus | -0.11108392 | 0.64104199 | negative |
| 8.05_331.1768m/z | s_Cutibacterium_acnes | 0.10908821 | 0.6470854 | positive |
| 8.93_353.2795m/z | s_Cutibacterium_acnes | -0.10795838 | 0.65051622 | negative |
| 13.74_173.2012m/z | s_Microbacterium_sp000800925 | 0.10739768 | 0.65222136 | positive |
| 6.95_167.0342m/z | s_Enterovirga_sp013044135 | 0.10717057 | 0.65291248 | positive |
| 6.92_169.0496m/z | s_Microbacterium_sp000800925 | -0.10699317 | 0.65345255 | negative |
| 7.02_311.2324m/z | s_Agrobacterium_sp900013535 | -0.10604377 | 0.65634556 | negative |
| 6.95_167.0342m/z | s_Microbacterium_sp005774735 | 0.1060153 | 0.65643237 | positive |
| 10.43_374.2816n | s_Microbacterium_sp005774735 | -0.1060153 | 0.65643237 | negative |
| 10.58_483.2972m/z | s_Enterovirga_sp013044135 | 0.10487604 | 0.65991031 | positive |
| 14.34_577.4818m/z | s_Sphingobium_yanoikuyae | 0.10474869 | 0.66029952 | positive |
| 8.93_353.2795m/z | s_Microbacterium_foliorum_A | 0.10444659 | 0.6612231 | positive |
| 6.95_167.0342m/z | s_Sphingobium_xenophagum | 0.10363368 | 0.66371068 | positive |
| 6.73_462.1779m/z | s_Erythrobacter_sp002215495 | -0.10247663 | 0.6672572 | negative |
| 11.08_253.0833m/z | s_Methylobacterium_rhodesianum | 0.10240976 | 0.6674624 | positive |
| 7.16_269.0596m/z | s_Meiothermus_B_silvanus | 0.10134234 | 0.67074058 | positive |
| 7.02_311.2324m/z | s_Sphingobium_yanoikuyae | -0.10104077 | 0.67166778 | negative |
| 11.08_253.0833m/z | s_Meiothermus_B_silvanus | -0.0993977 | 0.67672754 | negative |
| 8.71_455.2295m/z | s_Rhodococcus_qingshengii | -0.09863646 | 0.6790763 | negative |
| 12.02_573.4507m/z | s_Methylobacterium_rhodesianum | 0.09789168 | 0.68137704 | positive |
| 13.57_575.4662m/z | s_Microbacterium_foliorum_A | -0.09774694 | 0.68182446 | negative |
| 6.92_169.0496m/z | s_Erythrobacter_sp002215495 | -0.09774694 | 0.68182446 | negative |
| 6.91_314.9964m/z | s_Rhodococcus_qingshengii | 0.09547083 | 0.68887401 | positive |
| 14.34_577.4818m/z | s_Methylobacterium_rhodesianum | -0.09473684 | 0.69066153 | negative |
| 10.43_374.2816n | s_Microbacterium_foliorum_A | 0.09459382 | 0.69159691 | positive |
| 15.25_612.5196m/z | s_Sphingobium_yanoikuyae | -0.09455191 | 0.69172709 | negative |
| 11.08_253.0833m/z | s_Ochrobactrum_anthropi | -0.09434111 | 0.69238219 | negative |
| 12.02_573.4507m/z | s_Sphingobium_yanoikuyae | -0.09376604 | 0.69417029 | negative |
| 6.92_169.0496m/z | s_Microbacterium_sp005774735 | 0.09372367 | 0.6943021 | positive |
| 9.33_495.2973m/z | s_Cutibacterium_acnes | 0.09372367 | 0.6943021 | positive |
| 8.93_353.2795m/z | s_Methylobacterium_rhodesianum | -0.09358557 | 0.69473177 | negative |
| 15.09_593.4794m/z | s_Methylobacterium_rhodesianum | -0.09323308 | 0.69537578 | negative |
| 7.16_269.0596m/z | s_Sphingobium_xenophagum | -0.09281261 | 0.69713831 | negative |
| 6.73_462.1779m/z | s_Sphingobium_xenophagum | 0.09238662 | 0.69846581 | positive |
| 9.37_448.3053m/z | s_Ochrobactrum_anthropi | -0.09165321 | 0.70075323 | negative |
| 6.95_167.0342m/z | s_Ochrobactrum_anthropi | 0.09165321 | 0.70075323 | positive |
| 13.57_575.4662m/z | s_Sphingobium_xenophagum | 0.09158325 | 0.70097155 | positive |
| 9.33_495.2973m/z | s_Tepidimonas_fonticaldi | -0.09091104 | 0.70307051 | negative |
| 8.05_331.1768m/z | s_Tepidimonas_fonticaldi | 0.09091104 | 0.70307051 | positive |
| 6.92_169.0496m/z | s_Meiothermus_B_silvanus | -0.09022556 | 0.70483739 | negative |
| 5.17_287.0237m/z | s_Rhodococcus_qingshengii | 0.08881717 | 0.70962179 | positive |
| 0.64_1157.2640m/z | s_Cutibacterium_acnes | -0.08770985 | 0.71309432 | negative |
| 6.92_169.0496m/z | s_Rhodococcus_qingshengii | 0.08724058 | 0.71456759 | positive |
| 8.93_353.2795m/z | s_Enterovirga_sp013044135 | -0.08712121 | 0.7149425 | negative |
| 7.53_370.3060m/z | s_Tepidimonas_fonticaldi | 0.08695839 | 0.715454 | positive |
| 6.91_314.9964m/z | s_Sphingobium_xenophagum | 0.08676308 | 0.71606769 | positive |
| 8.05_331.1768m/z | s_Agrobacterium_pusense | 0.08571429 | 0.71911018 | positive |
| 6.91_314.9964m/z | s_Microbacterium_sp000800925 | 0.08559453 | 0.71974301 | positive |
| 7.53_370.3060m/z | s_Micrococcus_endophyticus | -0.08559453 | 0.71974301 | negative |
| 4.76_356.0497m/z | s_Meiothermus_B_silvanus | -0.0842422 | 0.72400375 | negative |
| 6.06_245.0920m/z | s_Methylobacterium_rhodesianum | -0.08421053 | 0.72388858 | negative |
| 11.08_253.0833m/z | s_Sphingobium_yanoikuyae | 0.0835539 | 0.72617538 | positive |
| 5.17_287.0237m/z | s_Agrobacterium_pusense | 0.08354964 | 0.72618884 | positive |
| 8.93_353.2795m/z | s_Tepidimonas_fonticaldi | 0.08331956 | 0.72691519 | positive |
| 8.44_399.2216m/z | s_Microbacterium_sp000800925 | 0.08230244 | 0.73012894 | positive |
| 6.92_169.0496m/z | s_Enterovirga_sp013044135 | 0.08226474 | 0.73024814 | positive |
| 5.17_287.0237m/z | s_Methylobacterium_rhodesianum | 0.08194291 | 0.73126596 | positive |
| 4.61_305.0341m/z | s_Erythrobacter_sp002215495 | -0.08185494 | 0.73154425 | negative |
| 2.15_274.0032m/z | s_Cutibacterium_acnes | -0.08175108 | 0.73187286 | negative |
| 8.71_455.2295m/z | s_Meiothermus_B_silvanus | -0.08156512 | 0.73246133 | negative |
| 0.64_1157.2640m/z | s_Microbacterium_sp000800925 | -0.08077795 | 0.7349539 | negative |
| 6.95_123.0441m/z | s_Microbacterium_sp000800925 | -0.08065639 | 0.73533905 | negative |
| 13.57_575.4662m/z | s_Micrococcus_endophyticus | -0.08065639 | 0.73533905 | negative |
| 15.25_612.5196m/z | s_Ochrobactrum_anthropi | -0.08062088 | 0.73545156 | negative |
| 7.02_311.2324m/z | s_Microbacterium_foliorum_A | -0.08040474 | 0.73613655 | negative |
| 6.92_169.0496m/z | s_Microbacterium_foliorum_A | 0.08040474 | 0.73613655 | positive |
| 12.02_573.4507m/z | s_Meiothermus_B_silvanus | -0.07831334 | 0.74277449 | negative |
| 6.95_167.0342m/z | s_Rhodococcus_qingshengii | 0.07736429 | 0.74579252 | positive |
| 10.58_483.2972m/z | s_Cutibacterium_acnes | 0.07698888 | 0.74698732 | positive |
| 9.37_448.3053m/z | s_Cutibacterium_acnes | -0.07682268 | 0.74751645 | negative |
| 4.61_305.0341m/z | s_Rhodococcus_qingshengii | 0.07617319 | 0.74958532 | positive |
| 10.58_483.2972m/z | s_Agrobacterium_pusense | 0.07612744 | 0.74973109 | positive |
| 4.76_356.0497m/z | s_Sphingobium_yanoikuyae | 0.07557725 | 0.75148502 | positive |
| 13.74_173.2012m/z | s_Micrococcus_endophyticus | -0.07517838 | 0.75275732 | negative |
| 8.44_399.2216m/z | s_Tepidimonas_fonticaldi | -0.07510043 | 0.75300602 | negative |
| 6.06_245.0920m/z | s_Cutibacterium_acnes | -0.07374978 | 0.75731915 | negative |
| 15.09_593.4794m/z | s_Cutibacterium_acnes | -0.07374978 | 0.75731915 | negative |
| 8.71_455.2295m/z | s_Micrococcus_endophyticus | 0.07312703 | 0.75931014 | positive |
| 8.44_399.2216m/z | s_Rhodococcus_qingshengii | -0.07242614 | 0.7615527 | negative |
| 2.15_274.0032m/z | s_Sphingobium_xenophagum | -0.07179499 | 0.76357372 | negative |
| 2.15_274.0032m/z | s_Microbacterium_foliorum_A | -0.07085452 | 0.76658793 | negative |
| 9.33_495.2973m/z | s_Meiothermus_B_silvanus | -0.07067669 | 0.76733013 | negative |
| 9.37_448.3053m/z | s_Enterovirga_sp013044135 | 0.07018918 | 0.76872229 | positive |
| 10.58_483.2972m/z | s_Meiothermus_B_silvanus | -0.06991296 | 0.76960886 | negative |
| 11.08_253.0833m/z | s_Agrobacterium_pusense | 0.06777116 | 0.77649252 | positive |
| 5.17_287.0237m/z | s_Meiothermus_B_silvanus | -0.06667904 | 0.7800088 | negative |
| 8.71_455.2295m/z | s_Sphingobium_yanoikuyae | 0.06608244 | 0.78193139 | positive |
| 6.02_248.1643m/z | s_Ochrobactrum_anthropi | 0.06534535 | 0.78430841 | positive |
| 6.02_248.1643m/z | s_Meiothermus_B_silvanus | 0.06466165 | 0.78686902 | positive |
| 6.91_314.9964m/z | s_Microbacterium_foliorum_A | -0.06306254 | 0.79168172 | negative |
| 8.71_455.2295m/z | s_Agrobacterium_pusense | 0.06214485 | 0.79465066 | positive |
| 15.25_612.5196m/z | s_Methylobacterium_rhodesianum | -0.06165414 | 0.79668675 | negative |
| 9.33_495.2973m/z | s_Microbacterium_foliorum_A | -0.06148598 | 0.79678393 | negative |
| 6.06_245.0920m/z | s_Sphingobium_xenophagum | -0.06025214 | 0.80078254 | negative |
| 15.25_612.5196m/z | s_Tepidimonas_fonticaldi | -0.05928981 | 0.80390457 | negative |
| 9.33_495.2973m/z | s_Micrococcus_endophyticus | -0.05925775 | 0.80400862 | negative |
| 7.16_269.0596m/z | s_Agrobacterium_pusense | 0.05867188 | 0.80591079 | positive |
| 9.37_448.3053m/z | s_Agrobacterium_sp900013535 | 0.05784206 | 0.80860681 | positive |
| 7.02_311.2324m/z | s_Tepidimonas_fonticaldi | 0.05731348 | 0.81032517 | positive |
| 8.71_455.2295m/z | s_Ochrobactrum_anthropi | -0.05699071 | 0.81137489 | negative |
| 6.72_464.1910m/z | s_Erythrobacter_sp002215495 | -0.05517973 | 0.81727033 | negative |
| 7.53_370.3060m/z | s_Agrobacterium_sp900013535 | -0.05462861 | 0.81906632 | negative |
| 7.53_370.3060m/z | s_Microbacterium_foliorum_A | 0.05360316 | 0.82241035 | positive |
| 0.64_1157.2640m/z | s_Enterovirga_sp013044135 | 0.05291019 | 0.82467184 | positive |
| 4.76_356.0497m/z | s_Microbacterium_sp000800925 | 0.05269337 | 0.82537969 | positive |
| 10.43_374.2816n | s_Ochrobactrum_anthropi | 0.05261573 | 0.82563319 | positive |
| 6.95_123.0441m/z | s_Sphingobium_yanoikuyae | 0.05098388 | 0.83096524 | positive |
| 13.57_575.4662m/z | s_Sphingobium_yanoikuyae | -0.05098388 | 0.83096524 | negative |
| 8.05_331.1768m/z | s_Agrobacterium_sp900013535 | -0.0506118 | 0.832182 | negative |
| 2.15_274.0032m/z | s_Agrobacterium_pusense | -0.04971588 | 0.83511331 | negative |
| 13.57_575.4662m/z | s_Methylobacterium_rhodesianum | 0.04962406 | 0.83624663 | positive |
| 7.16_269.0596m/z | s_Enterovirga_sp013044135 | -0.04933304 | 0.83636655 | negative |
| 14.34_577.4818m/z | s_Microbacterium_sp005774735 | 0.04916652 | 0.83691178 | positive |
| 9.37_448.3053m/z | s_Sphingobium_yanoikuyae | -0.04912992 | 0.83703163 | negative |
| 8.71_455.2295m/z | s_Microbacterium_foliorum_A | -0.04886519 | 0.83789859 | negative |
| 6.02_248.1643m/z | s_Sphingobium_xenophagum | 0.04659499 | 0.84534052 | positive |
| 6.92_169.0496m/z | s_Sphingobium_yanoikuyae | -0.04634898 | 0.84614774 | negative |
| 6.91_314.9964m/z | s_Sphingobium_yanoikuyae | -0.04634898 | 0.84614774 | negative |
| 6.91_314.9964m/z | s_Microbacterium_sp005774735 | 0.04609361 | 0.84698581 | positive |
| 7.53_370.3060m/z | s_Enterovirga_sp013044135 | -0.04603806 | 0.84716813 | negative |
| 13.74_173.2012m/z | s_Erythrobacter_sp002215495 | -0.04510194 | 0.85024181 | negative |
| 10.43_374.2816n | s_Sphingobium_xenophagum | 0.0441849 | 0.85325483 | positive |
| 8.05_331.1768m/z | s_Sphingobium_yanoikuyae | -0.04356804 | 0.8552827 | negative |
| 6.91_314.9964m/z | s_Enterovirga_sp013044135 | 0.04301917 | 0.85708778 | positive |
| 15.09_593.4794m/z | s_Sphingobium_xenophagum | 0.04257818 | 0.85853859 | positive |
| 5.17_287.0237m/z | s_Sphingobium_xenophagum | 0.0416309 | 0.86165647 | positive |
| 14.34_577.4818m/z | s_Ochrobactrum_anthropi | -0.0415834 | 0.86181287 | negative |
| 6.73_462.1779m/z | s_Microbacterium_sp005774735 | -0.04148425 | 0.86213935 | negative |
| 6.02_248.1643m/z | s_Rhodococcus_qingshengii | 0.04115122 | 0.86323606 | positive |
| 6.95_123.0441m/z | s_Microbacterium_sp005774735 | 0.03841134 | 0.87226785 | positive |
| 9.33_495.2973m/z | s_Agrobacterium_sp900013535 | -0.03775801 | 0.8744238 | negative |
| 4.61_305.0341m/z | s_Sphingobium_xenophagum | 0.03717668 | 0.87634285 | positive |
| 8.93_500.3035m/z | s_Enterovirga_sp013044135 | -0.03703713 | 0.87680363 | negative |
| 6.91_314.9964m/z | s_Methylobacterium_rhodesianum | -0.03609023 | 0.88121669 | negative |
| 4.61_305.0341m/z | s_Sphingobium_yanoikuyae | -0.03452707 | 0.88509793 | negative |
| 4.61_305.0341m/z | s_Ochrobactrum_anthropi | 0.03400383 | 0.88682839 | positive |
| 11.08_253.0833m/z | s_Cutibacterium_acnes | -0.03385293 | 0.88732757 | negative |
| 13.57_575.4662m/z | s_Agrobacterium_sp900013535 | 0.03293784 | 0.89035541 | positive |
| 15.25_612.5196m/z | s_Microbacterium_sp005774735 | 0.03226553 | 0.89258088 | positive |
| 6.72_464.1910m/z | s_Microbacterium_sp005774735 | -0.03226553 | 0.89258088 | negative |
| 8.44_399.2216m/z | s_Meiothermus_B_silvanus | 0.03007519 | 0.90132798 | positive |
| 11.08_253.0833m/z | s_Micrococcus_endophyticus | -0.02967353 | 0.90116797 | negative |
| 6.95_167.0342m/z | s_Sphingobium_yanoikuyae | -0.02966335 | 0.90120174 | negative |
| 15.09_593.4794m/z | s_Microbacterium_sp005774735 | -0.02919262 | 0.90276239 | negative |
| 6.06_245.0920m/z | s_Microbacterium_foliorum_A | -0.02837814 | 0.90546351 | negative |
| 11.08_253.0833m/z | s_Rhodococcus_qingshengii | -0.028025 | 0.90663499 | negative |
| 5.17_287.0237m/z | s_Cutibacterium_acnes | -0.02790816 | 0.90702262 | negative |
| 12.02_573.4507m/z | s_Agrobacterium_pusense | 0.02710846 | 0.90967627 | positive |
| 12.02_573.4507m/z | s_Agrobacterium_sp900013535 | -0.0265509 | 0.911527 | negative |
| 6.95_123.0441m/z | s_Sphingobium_xenophagum | 0.02490422 | 0.91699534 | positive |
| 6.91_314.9964m/z | s_Meiothermus_B_silvanus | -0.02406015 | 0.92149896 | negative |
| 13.57_575.4662m/z | s_Erythrobacter_sp002215495 | -0.02364845 | 0.92116794 | negative |
| 8.71_455.2295m/z | s_Erythrobacter_sp002215495 | -0.02198933 | 0.92668382 | negative |
| 7.53_370.3060m/z | s_Sphingobium_yanoikuyae | -0.02132053 | 0.92890824 | negative |
| 0.64_1157.2640m/z | s_Methylobacterium_rhodesianum | 0.02108436 | 0.92969385 | positive |
| 8.93_353.2795m/z | s_Agrobacterium_sp900013535 | 0.01935358 | 0.93545313 | positive |
| 8.93_500.3035m/z | s_Agrobacterium_sp900013535 | -0.01930974 | 0.93559904 | negative |
| 8.44_399.2216m/z | s_Microbacterium_foliorum_A | -0.01891876 | 0.93690051 | negative |
| 6.91_314.9964m/z | s_Erythrobacter_sp002215495 | -0.01891876 | 0.93690051 | negative |
| 10.58_483.2972m/z | s_Microbacterium_sp000800925 | 0.01870692 | 0.93760576 | positive |
| 8.05_331.1768m/z | s_Rhodococcus_qingshengii | -0.01810654 | 0.93960468 | negative |
| 9.33_495.2973m/z | s_Methylobacterium_rhodesianum | -0.01804511 | 0.9417175 | negative |
| 14.34_577.4818m/z | s_Tepidimonas_fonticaldi | -0.01778694 | 0.94066888 | negative |
| 8.93_353.2795m/z | s_Sphingobium_yanoikuyae | 0.0176792 | 0.94102767 | positive |
| 13.57_575.4662m/z | s_Meiothermus_B_silvanus | 0.01654135 | 0.94677813 | positive |
| 14.34_577.4818m/z | s_Microbacterium_foliorum_A | 0.01576564 | 0.94740176 | positive |
| 12.02_573.4507m/z | s_Cutibacterium_acnes | 0.01538769 | 0.94866106 | positive |
| 8.44_399.2216m/z | s_Agrobacterium_sp900013535 | -0.01526388 | 0.94907365 | negative |
| 9.33_495.2973m/z | s_Sphingobium_xenophagum | 0.01446051 | 0.95175091 | positive |
| 6.95_123.0441m/z | s_Enterovirga_sp013044135 | -0.01433973 | 0.95215349 | negative |
| 4.76_356.0497m/z | s_Microbacterium_sp005774735 | 0.01383328 | 0.95384155 | positive |
| 4.76_356.0497m/z | s_Erythrobacter_sp002215495 | -0.01182868 | 0.96052505 | negative |
| 8.71_455.2295m/z | s_Sphingobium_xenophagum | -0.0083 | 0.97229579 | negative |
| 11.08_253.0833m/z | s_Sphingobium_xenophagum | 0.00804573 | 0.97314422 | positive |
| 6.06_245.0920m/z | s_Tepidimonas_fonticaldi | 0.00790531 | 0.97361276 | positive |
| 13.57_575.4662m/z | s_Tepidimonas_fonticaldi | 0.00790531 | 0.97361276 | positive |
| 8.93_500.3035m/z | s_Methylobacterium_rhodesianum | -0.00753013 | 0.97486469 | negative |
| 8.05_331.1768m/z | s_Ochrobactrum_anthropi | -0.00339456 | 0.9886677 | negative |
| 8.44_399.2216m/z | s_Sphingobium_xenophagum | 0.00160672 | 0.99463604 | positive |
| 13.57_575.4662m/z | s_Agrobacterium_pusense | -0.00150376 | 0.99746386 | negative |
| 14.34_577.4818m/z | s_Sphingobium_xenophagum | 0 | 1 |  |

**Table S5 Spearman’s correlation between gallstone microbiota and metabolites**

| **Data1** | **Data2** | ***rho*** | ***P* value** | **Relation** |
| --- | --- | --- | --- | --- |
| 8.44_399.2216m/z | s_Erythrobacter_sp002215495 | 0.69961977 | 0.00059589 | positive |
| 9.33_495.2973m/z | s_Erythrobacter_sp002215495 | 0.68380916 | 0.00088588 | positive |
| 11.08_253.0833m/z | s_Agrobacterium_pusense | -0.66717456 | 0.00131156 | negative |
| 6.06_245.0920m/z | s_Erythrobacter_sp002215495 | -0.63242465 | 0.0027705 | negative |
| 15.09_593.4794m/z | s_Micrococcus_endophyticus | 0.61661404 | 0.00378427 | positive |
| 8.05_331.1768m/z | s_Micrococcus_endophyticus | 0.61463771 | 0.00393021 | positive |
| 15.25_612.5196m/z | s_Sphingobium_yanoikuyae | 0.61440725 | 0.00394753 | positive |
| 8.05_331.1768m/z | s_Microbacterium_foliorum_A | 0.61273348 | 0.00407521 | positive |
| 15.09_593.4794m/z | s_Sphingobium_yanoikuyae | 0.58779906 | 0.00641935 | positive |
| 5.17_287.0237m/z | s_Methylobacterium_rhodesianum | 0.56218341 | 0.00987983 | positive |
| 4.61_305.0341m/z | s_Methylobacterium_rhodesianum | 0.55332423 | 0.01138097 | positive |
| 4.61_305.0341m/z | s_Agrobacterium_pusense | 0.54484213 | 0.01298632 | positive |
| 8.93_353.2795m/z | s_Microbacterium_foliorum_A | -0.53146246 | 0.01588581 | negative |
| 14.34_577.4818m/z | s_Micrococcus_endophyticus | 0.52767932 | 0.0167935 | positive |
| 5.17_287.0237m/z | s_Agrobacterium_pusense | 0.52341911 | 0.01786489 | positive |
| 7.16_269.0596m/z | s_Sphingobium_yanoikuyae | -0.51479325 | 0.02020142 | negative |
| 7.16_269.0596m/z | s_Micrococcus_endophyticus | -0.51373259 | 0.02050477 | negative |
| 0.64_1157.2640m/z | s_Cutibacterium_acnes | -0.50696831 | 0.02252567 | negative |
| 0.64_1157.2640m/z | s_Micrococcus_endophyticus | -0.50472295 | 0.02323034 | negative |
| 6.06_245.0920m/z | s_Micrococcus_endophyticus | -0.49605809 | 0.02611501 | negative |
| 6.06_245.0920m/z | s_Sphingobium_yanoikuyae | -0.46685275 | 0.03796838 | negative |
| 15.09_593.4794m/z | s_Microbacterium_foliorum_A | 0.46627071 | 0.03824101 | positive |
| 8.93_500.3035m/z | s_Micrococcus_endophyticus | -0.46420897 | 0.03921899 | negative |
| 8.93_353.2795m/z | s_Micrococcus_endophyticus | -0.46000332 | 0.04127395 | negative |
| 14.34_577.4818m/z | s_Enterovirga_sp013044135 | 0.45878 | 0.04188704 | positive |
| 6.73_462.1779m/z | s_Agrobacterium_sp900013535 | -0.45817048 | 0.04219513 | negative |
| 6.91_314.9964m/z | s_Erythrobacter_sp002215495 | 0.45653155 | 0.04303223 | positive |
| 2.15_274.0032m/z | s_Sphingobium_yanoikuyae | -0.44984399 | 0.04658149 | negative |
| 9.37_448.3053m/z | s_Agrobacterium_pusense | 0.44962406 | 0.04818892 | positive |
| 13.57_575.4662m/z | s_Microbacterium_foliorum_A | 0.44031529 | 0.05202202 | positive |
| 6.72_464.1910m/z | s_Agrobacterium_sp900013535 | -0.43988668 | 0.05227766 | negative |
| 15.25_612.5196m/z | s_Micrococcus_endophyticus | 0.4387446 | 0.05296352 | positive |
| 10.43_374.2816n | s_Methylobacterium_rhodesianum | 0.43635283 | 0.05442211 | positive |
| 2.15_274.0032m/z | s_Methylobacterium_rhodesianum | -0.43574118 | 0.05479998 | negative |
| 14.34_577.4818m/z | s_Sphingobium_yanoikuyae | 0.42573101 | 0.06127219 | positive |
| 10.58_483.2972m/z | s_Sphingobium_yanoikuyae | -0.41844626 | 0.06633397 | negative |
| 4.61_305.0341m/z | s_Meiothermus_B_silvanus | 0.41809638 | 0.06658476 | positive |
| 8.93_353.2795m/z | s_Cutibacterium_acnes | -0.41531959 | 0.06860046 | negative |
| 5.17_287.0237m/z | s_Meiothermus_B_silvanus | 0.41463622 | 0.06910347 | positive |
| 7.53_370.3060m/z | s_Microbacterium_foliorum_A | -0.40045517 | 0.08017786 | negative |
| 6.91_314.9964m/z | s_Agrobacterium_sp900013535 | -0.40009253 | 0.08047733 | negative |
| 2.15_274.0032m/z | s_Micrococcus_endophyticus | -0.39918331 | 0.08123181 | negative |
| 6.95_123.0441m/z | s_Erythrobacter_sp002215495 | 0.39328908 | 0.08625063 | positive |
| 7.02_311.2324m/z | s_Cutibacterium_acnes | -0.38224229 | 0.09626712 | negative |
| 9.37_448.3053m/z | s_Micrococcus_endophyticus | -0.38143112 | 0.09703473 | negative |
| 13.57_575.4662m/z | s_Enterovirga_sp013044135 | 0.37995182 | 0.09844607 | positive |
| 10.43_374.2816n | s_Sphingobium_yanoikuyae | 0.37977141 | 0.09861922 | positive |
| 8.93_500.3035m/z | s_Sphingobium_xenophagum | 0.37939346 | 0.09898266 | positive |
| 12.02_573.4507m/z | s_Sphingobium_xenophagum | 0.37939346 | 0.09898266 | positive |
| 6.95_123.0441m/z | s_Agrobacterium_sp900013535 | -0.37858218 | 0.0997661 | negative |
| 5.17_287.0237m/z | s_Microbacterium_sp005774735 | 0.37837526 | 0.09996663 | positive |
| 6.73_462.1779m/z | s_Microbacterium_sp000800925 | -0.37796447 | 0.10036562 | negative |
| 6.72_464.1910m/z | s_Microbacterium_sp000800925 | -0.37796447 | 0.10036562 | negative |
| 6.73_462.1779m/z | s_Rhodococcus_qingshengii | -0.37796447 | 0.10036562 | negative |
| 6.72_464.1910m/z | s_Rhodococcus_qingshengii | -0.37796447 | 0.10036562 | negative |
| 7.53_370.3060m/z | s_Ochrobactrum_anthropi | -0.37796447 | 0.10036562 | negative |
| 9.33_495.2973m/z | s_Sphingobium_xenophagum | -0.37796447 | 0.10036562 | negative |
| 8.05_331.1768m/z | s_Tepidimonas_fonticaldi | -0.37796447 | 0.10036562 | negative |
| 15.25_612.5196m/z | s_Ochrobactrum_anthropi | 0.37796447 | 0.10036562 | positive |
| 15.09_593.4794m/z | s_Ochrobactrum_anthropi | 0.37796447 | 0.10036562 | positive |
| 9.37_448.3053m/z | s_Sphingobium_xenophagum | 0.37796447 | 0.10036562 | positive |
| 13.57_575.4662m/z | s_Sphingobium_xenophagum | 0.37796447 | 0.10036562 | positive |
| 10.43_374.2816n | s_Tepidimonas_fonticaldi | 0.37796447 | 0.10036562 | positive |
| 8.93_500.3035m/z | s_Microbacterium_foliorum_A | -0.37777659 | 0.1005485 | negative |
| 8.93_500.3035m/z | s_Agrobacterium_pusense | 0.37585173 | 0.10243601 | positive |
| 0.64_1157.2640m/z | s_Meiothermus_B_silvanus | 0.37048235 | 0.10783716 | positive |
| 4.61_305.0341m/z | s_Sphingobium_xenophagum | 0.37017802 | 0.10814933 | positive |
| 6.92_169.0496m/z | s_Erythrobacter_sp002215495 | 0.36957316 | 0.10877172 | positive |
| 13.57_575.4662m/z | s_Agrobacterium_sp900013535 | 0.36890252 | 0.10946482 | positive |
| 14.34_577.4818m/z | s_Microbacterium_foliorum_A | 0.36615692 | 0.11233561 | positive |
| 11.08_253.0833m/z | s_Agrobacterium_sp900013535 | 0.36597891 | 0.1125236 | positive |
| 6.02_248.1643m/z | s_Methylobacterium_rhodesianum | 0.36413951 | 0.11447929 | positive |
| 7.02_311.2324m/z | s_Microbacterium_foliorum_A | -0.363049 | 0.11565023 | negative |
| 8.05_331.1768m/z | s_Microbacterium_sp005774735 | 0.35488722 | 0.12513194 | positive |
| 5.17_287.0237m/z | s_Sphingobium_xenophagum | 0.35455159 | 0.12507024 | positive |
| 8.71_455.2295m/z | s_Tepidimonas_fonticaldi | 0.35455159 | 0.12507024 | positive |
| 4.61_305.0341m/z | s_Microbacterium_sp005774735 | 0.35060838 | 0.12962217 | positive |
| 13.74_173.2012m/z | s_Cutibacterium_acnes | -0.34805788 | 0.13262823 | negative |
| 7.16_269.0596m/z | s_Methylobacterium_rhodesianum | -0.34800696 | 0.13268874 | negative |
| 6.95_167.0342m/z | s_Erythrobacter_sp002215495 | 0.34585723 | 0.13526129 | positive |
| 6.06_245.0920m/z | s_Methylobacterium_rhodesianum | -0.34416562 | 0.13731023 | negative |
| 7.16_269.0596m/z | s_Sphingobium_xenophagum | 0.34271825 | 0.13908059 | positive |
| 11.08_253.0833m/z | s_Meiothermus_B_silvanus | -0.34188923 | 0.14010182 | negative |
| 10.58_483.2972m/z | s_Micrococcus_endophyticus | -0.34188173 | 0.14011108 | negative |
| 10.58_483.2972m/z | s_Methylobacterium_rhodesianum | -0.34149823 | 0.1405853 | negative |
| 14.34_577.4818m/z | s_Erythrobacter_sp002215495 | 0.33992825 | 0.14253839 | positive |
| 8.71_455.2295m/z | s_Agrobacterium_pusense | -0.33896117 | 0.1437509 | negative |
| 11.08_253.0833m/z | s_Microbacterium_sp005774735 | -0.33887034 | 0.14386515 | negative |
| 0.64_1157.2640m/z | s_Ochrobactrum_anthropi | -0.33868843 | 0.14409415 | negative |
| 0.64_1157.2640m/z | s_Sphingobium_xenophagum | 0.33868843 | 0.14409415 | positive |
| 6.06_245.0920m/z | s_Microbacterium_sp000800925 | -0.33817874 | 0.14473716 | negative |
| 6.02_248.1643m/z | s_Microbacterium_sp000800925 | -0.33817874 | 0.14473716 | negative |
| 6.06_245.0920m/z | s_Rhodococcus_qingshengii | -0.33817874 | 0.14473716 | negative |
| 6.02_248.1643m/z | s_Rhodococcus_qingshengii | -0.33817874 | 0.14473716 | negative |
| 9.37_448.3053m/z | s_Ochrobactrum_anthropi | -0.33817874 | 0.14473716 | negative |
| 13.57_575.4662m/z | s_Ochrobactrum_anthropi | 0.33817874 | 0.14473716 | positive |
| 14.34_577.4818m/z | s_Ochrobactrum_anthropi | 0.33817874 | 0.14473716 | positive |
| 6.06_245.0920m/z | s_Sphingobium_xenophagum | 0.33817874 | 0.14473716 | positive |
| 6.02_248.1643m/z | s_Sphingobium_xenophagum | 0.33817874 | 0.14473716 | positive |
| 13.74_173.2012m/z | s_Erythrobacter_sp002215495 | -0.33228633 | 0.15231704 | negative |
| 15.09_593.4794m/z | s_Enterovirga_sp013044135 | 0.32950179 | 0.15599334 | positive |
| 7.53_370.3060m/z | s_Cutibacterium_acnes | -0.32944718 | 0.15606604 | negative |
| 6.95_167.0342m/z | s_Micrococcus_endophyticus | 0.32609396 | 0.16057562 | positive |
| 13.74_173.2012m/z | s_Enterovirga_sp013044135 | -0.32599997 | 0.16070331 | negative |
| 11.08_253.0833m/z | s_Methylobacterium_rhodesianum | -0.32464627 | 0.16255007 | negative |
| 7.02_311.2324m/z | s_Micrococcus_endophyticus | -0.32226248 | 0.16583757 | negative |
| 6.02_248.1643m/z | s_Micrococcus_endophyticus | 0.32214131 | 0.16600589 | positive |
| 15.09_593.4794m/z | s_Erythrobacter_sp002215495 | 0.32214131 | 0.16600589 | positive |
| 8.93_500.3035m/z | s_Ochrobactrum_anthropi | -0.31948923 | 0.16971932 | negative |
| 13.74_173.2012m/z | s_Ochrobactrum_anthropi | -0.31948923 | 0.16971932 | negative |
| 12.02_573.4507m/z | s_Ochrobactrum_anthropi | -0.31948923 | 0.16971932 | negative |
| 11.08_253.0833m/z | s_Sphingobium_xenophagum | -0.31948923 | 0.16971932 | negative |
| 8.93_500.3035m/z | s_Tepidimonas_fonticaldi | -0.31948923 | 0.16971932 | negative |
| 8.05_331.1768m/z | s_Methylobacterium_rhodesianum | 0.314973 | 0.17617308 | positive |
| 6.73_462.1779m/z | s_Erythrobacter_sp002215495 | 0.314236 | 0.1772419 | positive |
| 6.72_464.1910m/z | s_Erythrobacter_sp002215495 | 0.314236 | 0.1772419 | positive |
| 9.37_448.3053m/z | s_Microbacterium_foliorum_A | -0.31239211 | 0.17993528 | negative |
| 6.73_462.1779m/z | s_Micrococcus_endophyticus | 0.30633069 | 0.18898449 | positive |
| 6.72_464.1910m/z | s_Micrococcus_endophyticus | 0.30435436 | 0.19200005 | positive |
| 6.02_248.1643m/z | s_Enterovirga_sp013044135 | 0.30427677 | 0.1921191 | positive |
| 6.92_169.0496m/z | s_Meiothermus_B_silvanus | 0.3037594 | 0.19245995 | positive |
| 6.95_123.0441m/z | s_Micrococcus_endophyticus | 0.30237804 | 0.19504777 | positive |
| 9.33_495.2973m/z | s_Meiothermus_B_silvanus | -0.30225564 | 0.19475668 | negative |
| 8.93_353.2795m/z | s_Ochrobactrum_anthropi | -0.30066219 | 0.19771995 | negative |
| 11.08_253.0833m/z | s_Ochrobactrum_anthropi | 0.29952115 | 0.19951043 | positive |
| 15.25_612.5196m/z | s_Erythrobacter_sp002215495 | 0.29842538 | 0.20124002 | positive |
| 6.95_167.0342m/z | s_Microbacterium_sp000800925 | -0.29839301 | 0.20129128 | negative |
| 6.92_169.0496m/z | s_Microbacterium_sp000800925 | -0.29839301 | 0.20129128 | negative |
| 6.95_123.0441m/z | s_Microbacterium_sp000800925 | -0.29839301 | 0.20129128 | negative |
| 6.95_167.0342m/z | s_Rhodococcus_qingshengii | -0.29839301 | 0.20129128 | negative |
| 6.92_169.0496m/z | s_Rhodococcus_qingshengii | -0.29839301 | 0.20129128 | negative |
| 6.95_123.0441m/z | s_Rhodococcus_qingshengii | -0.29839301 | 0.20129128 | negative |
| 10.43_374.2816n | s_Ochrobactrum_anthropi | 0.29839301 | 0.20129128 | positive |
| 8.05_331.1768m/z | s_Ochrobactrum_anthropi | 0.29839301 | 0.20129128 | positive |
| 6.73_462.1779m/z | s_Methylobacterium_rhodesianum | 0.2949991 | 0.20671252 | positive |
| 5.17_287.0237m/z | s_Micrococcus_endophyticus | 0.29422549 | 0.20796166 | positive |
| 10.43_374.2816n | s_Meiothermus_B_silvanus | 0.29323308 | 0.20893228 | positive |
| 10.43_374.2816n | s_Micrococcus_endophyticus | 0.2924964 | 0.21077164 | positive |
| 4.76_356.0497m/z | s_Meiothermus_B_silvanus | -0.29216901 | 0.21130651 | negative |
| 6.72_464.1910m/z | s_Methylobacterium_rhodesianum | 0.29038974 | 0.21422899 | positive |
| 8.05_331.1768m/z | s_Meiothermus_B_silvanus | 0.29022556 | 0.21380904 | positive |
| 7.02_311.2324m/z | s_Enterovirga_sp013044135 | 0.29019682 | 0.21454746 | positive |
| 6.91_314.9964m/z | s_Cutibacterium_acnes | -0.28958257 | 0.21556351 | negative |
| 13.57_575.4662m/z | s_Micrococcus_endophyticus | 0.28854375 | 0.21728907 | positive |
| 15.25_612.5196m/z | s_Microbacterium_foliorum_A | 0.2855097 | 0.22238071 | positive |
| 13.74_173.2012m/z | s_Agrobacterium_sp900013535 | -0.28393054 | 0.22506143 | negative |
| 6.91_314.9964m/z | s_Meiothermus_B_silvanus | 0.28270677 | 0.22633536 | positive |
| 7.16_269.0596m/z | s_Meiothermus_B_silvanus | 0.2826918 | 0.22717895 | positive |
| 7.16_269.0596m/z | s_Microbacterium_sp000800925 | -0.28223856 | 0.22795695 | negative |
| 7.16_269.0596m/z | s_Rhodococcus_qingshengii | -0.28223856 | 0.22795695 | negative |
| 7.16_269.0596m/z | s_Ochrobactrum_anthropi | -0.28223856 | 0.22795695 | negative |
| 7.16_269.0596m/z | s_Tepidimonas_fonticaldi | -0.28223856 | 0.22795695 | negative |
| 10.43_374.2816n | s_Microbacterium_foliorum_A | 0.28180178 | 0.22870834 | positive |
| 13.74_173.2012m/z | s_Micrococcus_endophyticus | -0.28169946 | 0.22888461 | negative |
| 7.16_269.0596m/z | s_Microbacterium_foliorum_A | -0.28088741 | 0.23028655 | negative |
| 8.71_455.2295m/z | s_Sphingobium_yanoikuyae | -0.27896418 | 0.23362909 | negative |
| 7.02_311.2324m/z | s_Erythrobacter_sp002215495 | -0.27678986 | 0.23744565 | negative |
| 15.25_612.5196m/z | s_Enterovirga_sp013044135 | 0.27116894 | 0.24749753 | positive |
| 10.58_483.2972m/z | s_Tepidimonas_fonticaldi | 0.27112769 | 0.24757229 | positive |
| 13.57_575.4662m/z | s_Meiothermus_B_silvanus | 0.27067669 | 0.24737928 | positive |
| 8.44_399.2216m/z | s_Enterovirga_sp013044135 | 0.26643925 | 0.25616331 | positive |
| 6.92_169.0496m/z | s_Micrococcus_endophyticus | 0.26087517 | 0.26660127 | positive |
| 4.76_356.0497m/z | s_Microbacterium_foliorum_A | -0.26087384 | 0.26660379 | negative |
| 8.93_353.2795m/z | s_Sphingobium_xenophagum | 0.2605739 | 0.26717396 | positive |
| 7.02_311.2324m/z | s_Sphingobium_xenophagum | 0.25870455 | 0.27074467 | positive |
| 6.91_314.9964m/z | s_Microbacterium_sp000800925 | -0.25860727 | 0.27093129 | negative |
| 6.91_314.9964m/z | s_Rhodococcus_qingshengii | -0.25860727 | 0.27093129 | negative |
| 7.53_370.3060m/z | s_Sphingobium_xenophagum | 0.25860727 | 0.27093129 | positive |
| 15.09_593.4794m/z | s_Tepidimonas_fonticaldi | -0.25860727 | 0.27093129 | negative |
| 6.95_123.0441m/z | s_Tepidimonas_fonticaldi | 0.25860727 | 0.27093129 | positive |
| 8.05_331.1768m/z | s_Enterovirga_sp013044135 | 0.2554033 | 0.27712312 | positive |
| 6.72_464.1910m/z | s_Sphingobium_yanoikuyae | 0.25398725 | 0.27988753 | positive |
| 10.43_374.2816n | s_Microbacterium_sp005774735 | 0.25263158 | 0.28127942 | positive |
| 10.58_483.2972m/z | s_Microbacterium_sp005774735 | -0.25225017 | 0.28330195 | negative |
| 7.53_370.3060m/z | s_Micrococcus_endophyticus | -0.25099353 | 0.28578801 | negative |
| 6.95_167.0342m/z | s_Agrobacterium_sp900013535 | -0.25059559 | 0.28657808 | negative |
| 11.08_253.0833m/z | s_Erythrobacter_sp002215495 | -0.24896678 | 0.28982592 | negative |
| 2.15_274.0032m/z | s_Microbacterium_sp000800925 | -0.24662973 | 0.29452535 | negative |
| 2.15_274.0032m/z | s_Rhodococcus_qingshengii | -0.24662973 | 0.29452535 | negative |
| 2.15_274.0032m/z | s_Ochrobactrum_anthropi | -0.24662973 | 0.29452535 | negative |
| 6.92_169.0496m/z | s_Agrobacterium_sp900013535 | -0.24629352 | 0.29520523 | negative |
| 6.95_167.0342m/z | s_Microbacterium_foliorum_A | 0.24564958 | 0.29651007 | positive |
| 9.33_495.2973m/z | s_Micrococcus_endophyticus | 0.24506455 | 0.29769859 | positive |
| 0.64_1157.2640m/z | s_Sphingobium_yanoikuyae | -0.24467976 | 0.2984819 | negative |
| 4.61_305.0341m/z | s_Agrobacterium_sp900013535 | -0.24369812 | 0.3004859 | negative |
| 0.64_1157.2640m/z | s_Microbacterium_foliorum_A | -0.24230631 | 0.30334126 | negative |
| 14.34_577.4818m/z | s_Methylobacterium_rhodesianum | 0.24122322 | 0.30557461 | positive |
| 12.02_573.4507m/z | s_Enterovirga_sp013044135 | 0.24054367 | 0.30698093 | positive |
| 7.53_370.3060m/z | s_Enterovirga_sp013044135 | 0.23963766 | 0.30886198 | positive |
| 9.33_495.2973m/z | s_Sphingobium_yanoikuyae | 0.23947369 | 0.30920316 | positive |
| 6.92_169.0496m/z | s_Microbacterium_foliorum_A | 0.23359885 | 0.321577 | positive |
| 10.43_374.2816n | s_Erythrobacter_sp002215495 | 0.23320659 | 0.32241355 | positive |
| 10.58_483.2972m/z | s_Enterovirga_sp013044135 | -0.23305785 | 0.3227311 | negative |
| 9.33_495.2973m/z | s_Microbacterium_foliorum_A | 0.22989093 | 0.32953651 | positive |
| 6.73_462.1779m/z | s_Sphingobium_yanoikuyae | 0.22979799 | 0.32973751 | positive |
| 5.17_287.0237m/z | s_Enterovirga_sp013044135 | 0.22975207 | 0.32983684 | positive |
| 10.58_483.2972m/z | s_Microbacterium_sp000800925 | -0.22941573 | 0.33056493 | negative |
| 10.58_483.2972m/z | s_Rhodococcus_qingshengii | -0.22941573 | 0.33056493 | negative |
| 8.71_455.2295m/z | s_Ochrobactrum_anthropi | -0.22941573 | 0.33056493 | negative |
| 10.58_483.2972m/z | s_Ochrobactrum_anthropi | -0.22941573 | 0.33056493 | negative |
| 8.71_455.2295m/z | s_Sphingobium_xenophagum | -0.22941573 | 0.33056493 | negative |
| 5.17_287.0237m/z | s_Tepidimonas_fonticaldi | -0.22941573 | 0.33056493 | negative |
| 8.71_455.2295m/z | s_Microbacterium_sp000800925 | 0.22941573 | 0.33056493 | positive |
| 8.71_455.2295m/z | s_Rhodococcus_qingshengii | 0.22941573 | 0.33056493 | positive |
| 5.17_287.0237m/z | s_Ochrobactrum_anthropi | 0.22941573 | 0.33056493 | positive |
| 7.16_269.0596m/z | s_Erythrobacter_sp002215495 | -0.22331846 | 0.34392855 | negative |
| 15.09_593.4794m/z | s_Methylobacterium_rhodesianum | 0.22278578 | 0.3451108 | positive |
| 8.71_455.2295m/z | s_Enterovirga_sp013044135 | -0.2214876 | 0.34800191 | negative |
| 8.05_331.1768m/z | s_Sphingobium_yanoikuyae | 0.22012228 | 0.35105769 | positive |
| 4.76_356.0497m/z | s_Microbacterium_sp000800925 | 0.21915134 | 0.35324022 | positive |
| 4.76_356.0497m/z | s_Rhodococcus_qingshengii | 0.21915134 | 0.35324022 | positive |
| 7.02_311.2324m/z | s_Microbacterium_sp000800925 | 0.21890385 | 0.35379779 | positive |
| 7.02_311.2324m/z | s_Rhodococcus_qingshengii | 0.21890385 | 0.35379779 | positive |
| 15.25_612.5196m/z | s_Microbacterium_sp000800925 | 0.21882154 | 0.35398334 | positive |
| 7.53_370.3060m/z | s_Microbacterium_sp000800925 | 0.21882154 | 0.35398334 | positive |
| 15.09_593.4794m/z | s_Microbacterium_sp000800925 | 0.21882154 | 0.35398334 | positive |
| 15.25_612.5196m/z | s_Rhodococcus_qingshengii | 0.21882154 | 0.35398334 | positive |
| 7.53_370.3060m/z | s_Rhodococcus_qingshengii | 0.21882154 | 0.35398334 | positive |
| 15.09_593.4794m/z | s_Rhodococcus_qingshengii | 0.21882154 | 0.35398334 | positive |
| 6.02_248.1643m/z | s_Ochrobactrum_anthropi | 0.21882154 | 0.35398334 | positive |
| 6.95_167.0342m/z | s_Tepidimonas_fonticaldi | 0.21882154 | 0.35398334 | positive |
| 9.33_495.2973m/z | s_Tepidimonas_fonticaldi | 0.21882154 | 0.35398334 | positive |
| 6.92_169.0496m/z | s_Tepidimonas_fonticaldi | 0.21882154 | 0.35398334 | positive |
| 6.91_314.9964m/z | s_Tepidimonas_fonticaldi | 0.21882154 | 0.35398334 | positive |
| 6.06_245.0920m/z | s_Tepidimonas_fonticaldi | -0.21882154 | 0.35398334 | negative |
| 6.06_245.0920m/z | s_Agrobacterium_pusense | 0.21804511 | 0.3541157 | positive |
| 8.93_353.2795m/z | s_Enterovirga_sp013044135 | 0.21604319 | 0.36027938 | positive |
| 4.61_305.0341m/z | s_Sphingobium_yanoikuyae | 0.21447267 | 0.36386655 | positive |
| 2.15_274.0032m/z | s_Agrobacterium_sp900013535 | -0.21390209 | 0.36517483 | negative |
| 7.53_370.3060m/z | s_Methylobacterium_rhodesianum | 0.2120306 | 0.36948478 | positive |
| 6.95_167.0342m/z | s_Meiothermus_B_silvanus | 0.21203008 | 0.36782906 | positive |
| 10.58_483.2972m/z | s_Agrobacterium_sp900013535 | -0.21085905 | 0.37219744 | negative |
| 13.57_575.4662m/z | s_Microbacterium_sp005774735 | 0.21052632 | 0.37130519 | positive |
| 6.95_123.0441m/z | s_Methylobacterium_rhodesianum | 0.21049415 | 0.37304464 | positive |
| 6.02_248.1643m/z | s_Microbacterium_sp005774735 | 0.20902256 | 0.37480036 | positive |
| 10.43_374.2816n | s_Cutibacterium_acnes | -0.20834902 | 0.37804708 | negative |
| 6.95_123.0441m/z | s_Sphingobium_yanoikuyae | 0.20802765 | 0.37879974 | positive |
| 5.17_287.0237m/z | s_Sphingobium_yanoikuyae | 0.20288304 | 0.39096308 | positive |
| 15.25_612.5196m/z | s_Methylobacterium_rhodesianum | 0.20281188 | 0.39113282 | positive |
| 9.37_448.3053m/z | s_Methylobacterium_rhodesianum | 0.20127543 | 0.39480786 | positive |
| 6.06_245.0920m/z | s_Microbacterium_foliorum_A | -0.20115456 | 0.39509776 | negative |
| 4.76_356.0497m/z | s_Sphingobium_yanoikuyae | -0.20107347 | 0.39529234 | negative |
| 2.15_274.0032m/z | s_Enterovirga_sp013044135 | -0.19953284 | 0.39899894 | negative |
| 2.15_274.0032m/z | s_Microbacterium_foliorum_A | -0.19920502 | 0.39979009 | negative |
| 14.34_577.4818m/z | s_Agrobacterium_sp900013535 | 0.19897075 | 0.40035602 | positive |
| 6.06_245.0920m/z | s_Meiothermus_B_silvanus | 0.19849624 | 0.39979587 | positive |
| 13.74_173.2012m/z | s_Methylobacterium_rhodesianum | -0.19818074 | 0.40226764 | negative |
| 6.95_167.0342m/z | s_Enterovirga_sp013044135 | 0.19707045 | 0.40496271 | positive |
| 9.37_448.3053m/z | s_Cutibacterium_acnes | 0.19706658 | 0.40497211 | positive |
| 9.33_495.2973m/z | s_Methylobacterium_rhodesianum | -0.19666607 | 0.40594673 | negative |
| 4.61_305.0341m/z | s_Microbacterium_sp000800925 | -0.1959766 | 0.40762748 | negative |
| 4.61_305.0341m/z | s_Rhodococcus_qingshengii | -0.1959766 | 0.40762748 | negative |
| 4.61_305.0341m/z | s_Tepidimonas_fonticaldi | -0.1959766 | 0.40762748 | negative |
| 4.61_305.0341m/z | s_Ochrobactrum_anthropi | 0.1959766 | 0.40762748 | positive |
| 6.91_314.9964m/z | s_Micrococcus_endophyticus | 0.19565638 | 0.40840939 | positive |
| 6.92_169.0496m/z | s_Microbacterium_sp005774735 | 0.19548872 | 0.40710613 | positive |
| 8.44_399.2216m/z | s_Microbacterium_sp005774735 | 0.19548872 | 0.40710613 | positive |
| 6.02_248.1643m/z | s_Meiothermus_B_silvanus | 0.19548872 | 0.40710613 | positive |
| 7.53_370.3060m/z | s_Erythrobacter_sp002215495 | -0.19368005 | 0.41325315 | negative |
| 2.15_274.0032m/z | s_Erythrobacter_sp002215495 | -0.19295562 | 0.41503642 | negative |
| 6.92_169.0496m/z | s_Cutibacterium_acnes | -0.19104928 | 0.41974883 | negative |
| 6.95_123.0441m/z | s_Cutibacterium_acnes | -0.19029712 | 0.42161604 | negative |
| 10.43_374.2816n | s_Agrobacterium_sp900013535 | 0.18929109 | 0.42412039 | positive |
| 4.76_356.0497m/z | s_Microbacterium_sp005774735 | -0.18825323 | 0.42671229 | negative |
| 6.73_462.1779m/z | s_Microbacterium_foliorum_A | 0.18724987 | 0.42922602 | positive |
| 8.71_455.2295m/z | s_Methylobacterium_rhodesianum | -0.18685752 | 0.43021111 | negative |
| 0.64_1157.2640m/z | s_Erythrobacter_sp002215495 | -0.18605473 | 0.43223046 | negative |
| 6.72_464.1910m/z | s_Microbacterium_foliorum_A | 0.18539591 | 0.43389141 | positive |
| 9.33_495.2973m/z | s_Agrobacterium_pusense | -0.18045113 | 0.44476034 | negative |
| 8.93_353.2795m/z | s_Microbacterium_sp000800925 | 0.18039731 | 0.44660248 | positive |
| 8.93_353.2795m/z | s_Rhodococcus_qingshengii | 0.18039731 | 0.44660248 | positive |
| 6.95_123.0441m/z | s_Enterovirga_sp013044135 | 0.17972825 | 0.44831842 | positive |
| 13.74_173.2012m/z | s_Microbacterium_sp000800925 | -0.17971269 | 0.44835836 | negative |
| 13.74_173.2012m/z | s_Rhodococcus_qingshengii | -0.17971269 | 0.44835836 | negative |
| 13.74_173.2012m/z | s_Agrobacterium_pusense | -0.17962392 | 0.44858629 | negative |
| 4.76_356.0497m/z | s_Ochrobactrum_anthropi | -0.17930564 | 0.44940404 | negative |
| 6.72_464.1910m/z | s_Ochrobactrum_anthropi | 0.1790358 | 0.45009791 | positive |
| 8.44_399.2216m/z | s_Ochrobactrum_anthropi | -0.1790358 | 0.45009791 | negative |
| 10.43_374.2816n | s_Sphingobium_xenophagum | 0.1790358 | 0.45009791 | positive |
| 6.92_169.0496m/z | s_Sphingobium_xenophagum | 0.1790358 | 0.45009791 | positive |
| 6.91_314.9964m/z | s_Sphingobium_xenophagum | 0.1790358 | 0.45009791 | positive |
| 15.09_593.4794m/z | s_Sphingobium_xenophagum | -0.1790358 | 0.45009791 | negative |
| 14.34_577.4818m/z | s_Sphingobium_xenophagum | 0.1790358 | 0.45009791 | positive |
| 6.95_123.0441m/z | s_Sphingobium_xenophagum | 0.1790358 | 0.45009791 | positive |
| 15.25_612.5196m/z | s_Tepidimonas_fonticaldi | -0.1790358 | 0.45009791 | negative |
| 13.57_575.4662m/z | s_Tepidimonas_fonticaldi | -0.1790358 | 0.45009791 | negative |
| 8.93_500.3035m/z | s_Methylobacterium_rhodesianum | 0.17813133 | 0.4524278 | positive |
| 4.61_305.0341m/z | s_Micrococcus_endophyticus | 0.17522988 | 0.45994362 | positive |
| 13.57_575.4662m/z | s_Sphingobium_yanoikuyae | 0.17416269 | 0.46272401 | positive |
| 8.44_399.2216m/z | s_Microbacterium_foliorum_A | 0.17334518 | 0.46485967 | positive |
| 6.02_248.1643m/z | s_Agrobacterium_sp900013535 | -0.17315833 | 0.4653485 | negative |
| 8.93_500.3035m/z | s_Meiothermus_B_silvanus | 0.17056725 | 0.47215398 | positive |
| 6.92_169.0496m/z | s_Methylobacterium_rhodesianum | 0.17054635 | 0.47220907 | positive |
| 10.58_483.2972m/z | s_Microbacterium_foliorum_A | -0.17007534 | 0.47345163 | negative |
| 6.95_167.0342m/z | s_Cutibacterium_acnes | -0.16848441 | 0.47766068 | negative |
| 7.16_269.0596m/z | s_Agrobacterium_sp900013535 | -0.16676307 | 0.48223572 | negative |
| 15.25_612.5196m/z | s_Agrobacterium_sp900013535 | 0.1656297 | 0.48525984 | positive |
| 8.93_353.2795m/z | s_Methylobacterium_rhodesianum | 0.16255448 | 0.49351241 | positive |
| 6.92_169.0496m/z | s_Enterovirga_sp013044135 | 0.16238605 | 0.49396637 | positive |
| 8.44_399.2216m/z | s_Cutibacterium_acnes | -0.16171494 | 0.49577722 | negative |
| 8.44_399.2216m/z | s_Micrococcus_endophyticus | 0.15810616 | 0.50556999 | positive |
| 13.57_575.4662m/z | s_Agrobacterium_pusense | 0.15639098 | 0.50869251 | positive |
| 8.93_353.2795m/z | s_Microbacterium_sp005774735 | 0.15606509 | 0.51114952 | positive |
| 9.37_448.3053m/z | s_Sphingobium_yanoikuyae | -0.15481128 | 0.51459147 | negative |
| 6.72_464.1910m/z | s_Enterovirga_sp013044135 | -0.15292667 | 0.5197857 | negative |
| 4.61_305.0341m/z | s_Enterovirga_sp013044135 | 0.15186528 | 0.52272185 | positive |
| 10.58_483.2972m/z | s_Cutibacterium_acnes | 0.15061846 | 0.5261809 | positive |
| 6.91_314.9964m/z | s_Microbacterium_sp005774735 | 0.15037594 | 0.52535073 | positive |
| 6.91_314.9964m/z | s_Microbacterium_foliorum_A | 0.15017069 | 0.52742574 | positive |
| 5.17_287.0237m/z | s_Erythrobacter_sp002215495 | 0.14711274 | 0.53596363 | positive |
| 10.58_483.2972m/z | s_Sphingobium_xenophagum | 0.14599183 | 0.5391091 | positive |
| 12.02_573.4507m/z | s_Agrobacterium_pusense | 0.14566141 | 0.54003792 | positive |
| 7.16_269.0596m/z | s_Agrobacterium_pusense | 0.14553675 | 0.54038855 | positive |
| 13.74_173.2012m/z | s_Microbacterium_sp005774735 | -0.14490669 | 0.54216221 | negative |
| 15.09_593.4794m/z | s_Agrobacterium_sp900013535 | 0.14304383 | 0.54742178 | positive |
| 2.15_274.0032m/z | s_Microbacterium_sp005774735 | -0.14293316 | 0.54773499 | negative |
| 11.08_253.0833m/z | s_Microbacterium_foliorum_A | 0.1414336 | 0.55198659 | positive |
| 6.95_167.0342m/z | s_Microbacterium_sp005774735 | 0.14135338 | 0.55081893 | positive |
| 6.02_248.1643m/z | s_Sphingobium_yanoikuyae | 0.14029772 | 0.5552169 | positive |
| 7.02_311.2324m/z | s_Methylobacterium_rhodesianum | 0.13986987 | 0.55643583 | positive |
| 6.91_314.9964m/z | s_Methylobacterium_rhodesianum | 0.13981728 | 0.55658575 | positive |
| 12.02_573.4507m/z | s_Tepidimonas_fonticaldi | -0.13977654 | 0.55670191 | negative |
| 0.64_1157.2640m/z | s_Tepidimonas_fonticaldi | 0.13945994 | 0.55760484 | positive |
| 7.02_311.2324m/z | s_Ochrobactrum_anthropi | -0.13930245 | 0.55805426 | negative |
| 10.43_374.2816n | s_Microbacterium_sp000800925 | 0.13925007 | 0.55820376 | positive |
| 10.43_374.2816n | s_Rhodococcus_qingshengii | 0.13925007 | 0.55820376 | positive |
| 6.73_462.1779m/z | s_Ochrobactrum_anthropi | 0.13925007 | 0.55820376 | positive |
| 6.95_123.0441m/z | s_Ochrobactrum_anthropi | 0.13925007 | 0.55820376 | positive |
| 6.95_167.0342m/z | s_Sphingobium_xenophagum | 0.13925007 | 0.55820376 | positive |
| 8.05_331.1768m/z | s_Sphingobium_xenophagum | -0.13925007 | 0.55820376 | negative |
| 8.44_399.2216m/z | s_Tepidimonas_fonticaldi | 0.13925007 | 0.55820376 | positive |
| 2.15_274.0032m/z | s_Cutibacterium_acnes | 0.1383243 | 0.56084905 | positive |
| 6.02_248.1643m/z | s_Microbacterium_foliorum_A | 0.13811995 | 0.5614337 | positive |
| 6.06_245.0920m/z | s_Cutibacterium_acnes | 0.13764574 | 0.56279149 | positive |
| 8.71_455.2295m/z | s_Micrococcus_endophyticus | -0.13675269 | 0.56535243 | negative |
| 6.73_462.1779m/z | s_Enterovirga_sp013044135 | -0.13558447 | 0.56871017 | negative |
| 6.91_314.9964m/z | s_Sphingobium_yanoikuyae | 0.13545987 | 0.56906882 | positive |
| 5.17_287.0237m/z | s_Agrobacterium_sp900013535 | -0.13531062 | 0.56949853 | negative |
| 11.08_253.0833m/z | s_Enterovirga_sp013044135 | -0.13530581 | 0.56951237 | negative |
| 8.93_353.2795m/z | s_Meiothermus_B_silvanus | 0.13485236 | 0.57081886 | positive |
| 11.08_253.0833m/z | s_Cutibacterium_acnes | 0.13476862 | 0.57106027 | positive |
| 15.09_593.4794m/z | s_Microbacterium_sp005774735 | 0.13233083 | 0.57684298 | positive |
| 6.95_167.0342m/z | s_Methylobacterium_rhodesianum | 0.13213501 | 0.57867514 | positive |
| 6.95_123.0441m/z | s_Meiothermus_B_silvanus | 0.13082707 | 0.5812326 | positive |
| 6.92_169.0496m/z | s_Sphingobium_yanoikuyae | 0.13062201 | 0.58306948 | positive |
| 6.02_248.1643m/z | s_Cutibacterium_acnes | -0.12937195 | 0.58671083 | negative |
| 12.02_573.4507m/z | s_Agrobacterium_sp900013535 | 0.12901026 | 0.5877662 | positive |
| 7.53_370.3060m/z | s_Sphingobium_yanoikuyae | -0.12820309 | 0.59012435 | negative |
| 14.34_577.4818m/z | s_Cutibacterium_acnes | -0.12786763 | 0.59110555 | negative |
| 9.33_495.2973m/z | s_Microbacterium_sp005774735 | -0.12781955 | 0.59005553 | negative |
| 14.34_577.4818m/z | s_Microbacterium_sp005774735 | 0.12781955 | 0.59005553 | positive |
| 4.76_356.0497m/z | s_Erythrobacter_sp002215495 | -0.12667556 | 0.59459783 | negative |
| 4.76_356.0497m/z | s_Micrococcus_endophyticus | -0.12073765 | 0.61212022 | negative |
| 7.53_370.3060m/z | s_Agrobacterium_sp900013535 | -0.12045797 | 0.61295066 | negative |
| 13.74_173.2012m/z | s_Sphingobium_yanoikuyae | -0.1153334 | 0.62824633 | negative |
| 6.91_314.9964m/z | s_Agrobacterium_pusense | 0.11428571 | 0.63045442 | positive |
| 2.15_274.0032m/z | s_Agrobacterium_pusense | 0.11419116 | 0.6316759 | positive |
| 7.02_311.2324m/z | s_Agrobacterium_sp900013535 | 0.11297182 | 0.635345 | positive |
| 7.16_269.0596m/z | s_Cutibacterium_acnes | -0.11281435 | 0.63581944 | negative |
| 0.64_1157.2640m/z | s_Methylobacterium_rhodesianum | -0.11233016 | 0.6372791 | negative |
| 8.71_455.2295m/z | s_Meiothermus_B_silvanus | -0.11193601 | 0.63846827 | negative |
| 4.76_356.0497m/z | s_Cutibacterium_acnes | -0.11073453 | 0.64209845 | negative |
| 10.58_483.2972m/z | s_Erythrobacter_sp002215495 | -0.10981656 | 0.64487733 | negative |
| 6.95_123.0441m/z | s_Microbacterium_foliorum_A | 0.10845661 | 0.64900247 | positive |
| 8.44_399.2216m/z | s_Agrobacterium_sp900013535 | 0.10755176 | 0.65175263 | positive |
| 0.64_1157.2640m/z | s_Enterovirga_sp013044135 | -0.1073679 | 0.65231196 | negative |
| 8.93_500.3035m/z | s_Sphingobium_yanoikuyae | -0.10683515 | 0.65393374 | negative |
| 13.57_575.4662m/z | s_Erythrobacter_sp002215495 | 0.10672166 | 0.6542794 | positive |
| 0.64_1157.2640m/z | s_Agrobacterium_sp900013535 | -0.10663671 | 0.65453818 | negative |
| 8.05_331.1768m/z | s_Erythrobacter_sp002215495 | 0.10474533 | 0.66030978 | positive |
| 8.71_455.2295m/z | s_Microbacterium_sp005774735 | -0.1040532 | 0.6624265 | negative |
| 6.95_167.0342m/z | s_Sphingobium_yanoikuyae | 0.10401383 | 0.66254698 | positive |
| 15.09_593.4794m/z | s_Cutibacterium_acnes | -0.10379843 | 0.66320627 | negative |
| 2.15_274.0032m/z | s_Sphingobium_xenophagum | 0.10276239 | 0.66638069 | positive |
| 10.43_374.2816n | s_Enterovirga_sp013044135 | 0.10247663 | 0.6672572 | positive |
| 15.25_612.5196m/z | s_Microbacterium_sp005774735 | 0.10225564 | 0.66726199 | positive |
| 8.93_353.2795m/z | s_Erythrobacter_sp002215495 | -0.10155917 | 0.67007419 | negative |
| 8.93_500.3035m/z | s_Microbacterium_sp000800925 | -0.09984038 | 0.67536301 | negative |
| 8.93_500.3035m/z | s_Rhodococcus_qingshengii | -0.09984038 | 0.67536301 | negative |
| 11.08_253.0833m/z | s_Tepidimonas_fonticaldi | 0.09984038 | 0.67536301 | positive |
| 4.76_356.0497m/z | s_Tepidimonas_fonticaldi | 0.09961424 | 0.67605995 | positive |
| 7.02_311.2324m/z | s_Tepidimonas_fonticaldi | 0.09950175 | 0.67640674 | positive |
| 9.37_448.3053m/z | s_Microbacterium_sp000800925 | 0.09946434 | 0.67652209 | positive |
| 8.44_399.2216m/z | s_Microbacterium_sp000800925 | 0.09946434 | 0.67652209 | positive |
| 8.05_331.1768m/z | s_Microbacterium_sp000800925 | -0.09946434 | 0.67652209 | negative |
| 13.57_575.4662m/z | s_Microbacterium_sp000800925 | -0.09946434 | 0.67652209 | negative |
| 9.37_448.3053m/z | s_Rhodococcus_qingshengii | 0.09946434 | 0.67652209 | positive |
| 8.44_399.2216m/z | s_Rhodococcus_qingshengii | 0.09946434 | 0.67652209 | positive |
| 8.05_331.1768m/z | s_Rhodococcus_qingshengii | -0.09946434 | 0.67652209 | negative |
| 13.57_575.4662m/z | s_Rhodococcus_qingshengii | -0.09946434 | 0.67652209 | negative |
| 6.95_167.0342m/z | s_Ochrobactrum_anthropi | 0.09946434 | 0.67652209 | positive |
| 6.06_245.0920m/z | s_Ochrobactrum_anthropi | -0.09946434 | 0.67652209 | negative |
| 9.33_495.2973m/z | s_Ochrobactrum_anthropi | -0.09946434 | 0.67652209 | negative |
| 6.92_169.0496m/z | s_Ochrobactrum_anthropi | 0.09946434 | 0.67652209 | positive |
| 15.25_612.5196m/z | s_Sphingobium_xenophagum | -0.09946434 | 0.67652209 | negative |
| 6.02_248.1643m/z | s_Tepidimonas_fonticaldi | 0.09946434 | 0.67652209 | positive |
| 6.72_464.1910m/z | s_Tepidimonas_fonticaldi | 0.09946434 | 0.67652209 | positive |
| 14.34_577.4818m/z | s_Tepidimonas_fonticaldi | -0.09946434 | 0.67652209 | negative |
| 9.33_495.2973m/z | s_Agrobacterium_sp900013535 | 0.09894762 | 0.67811591 | positive |
| 9.33_495.2973m/z | s_Cutibacterium_acnes | 0.09778113 | 0.68171877 | positive |
| 8.44_399.2216m/z | s_Agrobacterium_pusense | 0.09774436 | 0.68126691 | positive |
| 6.92_169.0496m/z | s_Agrobacterium_pusense | 0.0962406 | 0.68595852 | positive |
| 7.02_311.2324m/z | s_Microbacterium_sp005774735 | 0.09402032 | 0.69337946 | positive |
| 15.09_593.4794m/z | s_Agrobacterium_pusense | -0.09323308 | 0.69537578 | negative |
| 5.17_287.0237m/z | s_Microbacterium_foliorum_A | 0.09038289 | 0.7047211 | positive |
| 5.17_287.0237m/z | s_Cutibacterium_acnes | -0.08989793 | 0.70623784 | negative |
| 14.34_577.4818m/z | s_Meiothermus_B_silvanus | 0.08571429 | 0.71911018 | positive |
| 8.05_331.1768m/z | s_Agrobacterium_pusense | 0.08421053 | 0.72388858 | positive |
| 7.02_311.2324m/z | s_Agrobacterium_pusense | -0.0782249 | 0.74305558 | negative |
| 6.02_248.1643m/z | s_Agrobacterium_pusense | 0.07819549 | 0.74310181 | positive |
| 8.93_500.3035m/z | s_Agrobacterium_sp900013535 | -0.07719024 | 0.74634639 | negative |
| 7.02_311.2324m/z | s_Meiothermus_B_silvanus | 0.07521625 | 0.75263648 | positive |
| 13.74_173.2012m/z | s_Meiothermus_B_silvanus | 0.07471751 | 0.7542281 | positive |
| 0.64_1157.2640m/z | s_Microbacterium_sp005774735 | 0.07379526 | 0.75717377 | positive |
| 6.73_462.1779m/z | s_Meiothermus_B_silvanus | 0.06917293 | 0.77220234 | positive |
| 8.44_399.2216m/z | s_Meiothermus_B_silvanus | 0.06917293 | 0.77220234 | positive |
| 10.58_483.2972m/z | s_Meiothermus_B_silvanus | -0.06779223 | 0.77642471 | negative |
| 4.76_356.0497m/z | s_Enterovirga_sp013044135 | 0.06473653 | 0.78627315 | positive |
| 8.71_455.2295m/z | s_Erythrobacter_sp002215495 | -0.06423233 | 0.78790123 | negative |
| 5.17_287.0237m/z | s_Microbacterium_sp000800925 | 0.06256793 | 0.79328157 | positive |
| 5.17_287.0237m/z | s_Rhodococcus_qingshengii | 0.06256793 | 0.79328157 | positive |
| 8.93_353.2795m/z | s_Tepidimonas_fonticaldi | 0.06013244 | 0.80117073 | positive |
| 6.91_314.9964m/z | s_Enterovirga_sp013044135 | 0.05990942 | 0.80189409 | positive |
| 4.76_356.0497m/z | s_Sphingobium_xenophagum | -0.05976855 | 0.80235108 | negative |
| 9.33_495.2973m/z | s_Microbacterium_sp000800925 | 0.0596786 | 0.8026429 | positive |
| 14.34_577.4818m/z | s_Microbacterium_sp000800925 | 0.0596786 | 0.8026429 | positive |
| 9.33_495.2973m/z | s_Rhodococcus_qingshengii | 0.0596786 | 0.8026429 | positive |
| 14.34_577.4818m/z | s_Rhodococcus_qingshengii | 0.0596786 | 0.8026429 | positive |
| 6.73_462.1779m/z | s_Tepidimonas_fonticaldi | 0.0596786 | 0.8026429 | positive |
| 9.37_448.3053m/z | s_Tepidimonas_fonticaldi | -0.0596786 | 0.8026429 | negative |
| 12.02_573.4507m/z | s_Microbacterium_foliorum_A | 0.05955099 | 0.80305696 | positive |
| 6.72_464.1910m/z | s_Microbacterium_sp005774735 | -0.05864662 | 0.80653479 | negative |
| 8.44_399.2216m/z | s_Sphingobium_yanoikuyae | 0.05805423 | 0.80791728 | positive |
| 7.16_269.0596m/z | s_Microbacterium_sp005774735 | 0.05714794 | 0.81086352 | positive |
| 4.76_356.0497m/z | s_Agrobacterium_pusense | -0.05572296 | 0.81550091 | negative |
| 12.02_573.4507m/z | s_Methylobacterium_rhodesianum | -0.05475032 | 0.8186696 | negative |
| 8.93_353.2795m/z | s_Sphingobium_yanoikuyae | -0.05362107 | 0.82235192 | negative |
| 15.25_612.5196m/z | s_Agrobacterium_pusense | -0.05263158 | 0.82631617 | negative |
| 4.61_305.0341m/z | s_Cutibacterium_acnes | -0.05187004 | 0.82806883 | negative |
| 8.71_455.2295m/z | s_Agrobacterium_sp900013535 | 0.05074148 | 0.83175786 | positive |
| 6.06_245.0920m/z | s_Enterovirga_sp013044135 | -0.05045004 | 0.83271111 | negative |
| 6.72_464.1910m/z | s_Meiothermus_B_silvanus | 0.04962406 | 0.83624663 | positive |
| 14.34_577.4818m/z | s_Agrobacterium_pusense | -0.04962406 | 0.83624663 | negative |
| 15.25_612.5196m/z | s_Cutibacterium_acnes | -0.0481384 | 0.84027965 | negative |
| 6.95_123.0441m/z | s_Microbacterium_sp005774735 | 0.0481203 | 0.84122117 | positive |
| 11.08_253.0833m/z | s_Sphingobium_yanoikuyae | 0.04734739 | 0.84287263 | positive |
| 10.58_483.2972m/z | s_Agrobacterium_pusense | 0.04729691 | 0.84303818 | positive |
| 8.05_331.1768m/z | s_Agrobacterium_sp900013535 | 0.04624726 | 0.84648155 | positive |
| 9.37_448.3053m/z | s_Meiothermus_B_silvanus | 0.04511278 | 0.85118795 | positive |
| 15.09_593.4794m/z | s_Meiothermus_B_silvanus | 0.04511278 | 0.85118795 | positive |
| 12.02_573.4507m/z | s_Microbacterium_sp005774735 | -0.04452862 | 0.85212528 | negative |
| 9.37_448.3053m/z | s_Enterovirga_sp013044135 | -0.04256722 | 0.85857466 | negative |
| 6.73_462.1779m/z | s_Microbacterium_sp005774735 | -0.04210526 | 0.86117711 | negative |
| 8.93_500.3035m/z | s_Enterovirga_sp013044135 | -0.04114563 | 0.86325447 | negative |
| 4.76_356.0497m/z | s_Methylobacterium_rhodesianum | -0.040008 | 0.86700266 | negative |
| 13.57_575.4662m/z | s_Cutibacterium_acnes | -0.03836029 | 0.87243629 | negative |
| 13.74_173.2012m/z | s_Microbacterium_foliorum_A | -0.03814985 | 0.87313064 | negative |
| 8.05_331.1768m/z | s_Cutibacterium_acnes | -0.0361038 | 0.87988634 | negative |
| 8.93_353.2795m/z | s_Agrobacterium_pusense | 0.0318191 | 0.89405908 | positive |
| 6.02_248.1643m/z | s_Erythrobacter_sp002215495 | -0.03162123 | 0.89471434 | negative |
| 7.53_370.3060m/z | s_Microbacterium_sp005774735 | 0.03157895 | 0.89629409 | positive |
| 11.08_253.0833m/z | s_Micrococcus_endophyticus | 0.02975699 | 0.90089132 | positive |
| 8.44_399.2216m/z | s_Methylobacterium_rhodesianum | -0.02919262 | 0.90276239 | negative |
| 6.06_245.0920m/z | s_Agrobacterium_sp900013535 | 0.02688794 | 0.91040821 | positive |
| 8.71_455.2295m/z | s_Cutibacterium_acnes | 0.02681166 | 0.91066139 | positive |
| 13.57_575.4662m/z | s_Methylobacterium_rhodesianum | -0.02611971 | 0.91295853 | negative |
| 6.73_462.1779m/z | s_Cutibacterium_acnes | -0.0240692 | 0.91976967 | negative |
| 9.37_448.3053m/z | s_Microbacterium_sp005774735 | -0.02406015 | 0.92149896 | negative |
| 12.02_573.4507m/z | s_Micrococcus_endophyticus | -0.02380559 | 0.92064571 | negative |
| 4.61_305.0341m/z | s_Erythrobacter_sp002215495 | -0.02379665 | 0.92067541 | negative |
| 9.37_448.3053m/z | s_Erythrobacter_sp002215495 | -0.0217396 | 0.92751437 | negative |
| 2.15_274.0032m/z | s_Tepidimonas_fonticaldi | 0.02055248 | 0.93146338 | positive |
| 12.02_573.4507m/z | s_Microbacterium_sp000800925 | 0.01996808 | 0.93340799 | positive |
| 12.02_573.4507m/z | s_Rhodococcus_qingshengii | 0.01996808 | 0.93340799 | positive |
| 13.74_173.2012m/z | s_Tepidimonas_fonticaldi | 0.01996808 | 0.93340799 | positive |
| 11.08_253.0833m/z | s_Microbacterium_sp000800925 | -0.01996808 | 0.93340799 | negative |
| 11.08_253.0833m/z | s_Rhodococcus_qingshengii | -0.01996808 | 0.93340799 | negative |
| 13.74_173.2012m/z | s_Sphingobium_xenophagum | -0.01996808 | 0.93340799 | negative |
| 0.64_1157.2640m/z | s_Microbacterium_sp000800925 | 0.01992285 | 0.9335585 | positive |
| 0.64_1157.2640m/z | s_Rhodococcus_qingshengii | 0.01992285 | 0.9335585 | positive |
| 6.91_314.9964m/z | s_Ochrobactrum_anthropi | 0.01989287 | 0.93365828 | positive |
| 6.73_462.1779m/z | s_Sphingobium_xenophagum | 0.01989287 | 0.93365828 | positive |
| 6.72_464.1910m/z | s_Sphingobium_xenophagum | 0.01989287 | 0.93365828 | positive |
| 8.44_399.2216m/z | s_Sphingobium_xenophagum | 0.01989287 | 0.93365828 | positive |
| 7.53_370.3060m/z | s_Tepidimonas_fonticaldi | 0.01989287 | 0.93365828 | positive |
| 8.93_500.3035m/z | s_Cutibacterium_acnes | -0.01887516 | 0.93704567 | negative |
| 12.02_573.4507m/z | s_Erythrobacter_sp002215495 | -0.01785419 | 0.94044495 | negative |
| 9.33_495.2973m/z | s_Enterovirga_sp013044135 | 0.0173422 | 0.94214998 | positive |
| 7.16_269.0596m/z | s_Enterovirga_sp013044135 | -0.01677613 | 0.9440354 | negative |
| 12.02_573.4507m/z | s_Meiothermus_B_silvanus | -0.01660389 | 0.94460913 | negative |
| 7.53_370.3060m/z | s_Meiothermus_B_silvanus | -0.01654135 | 0.94677813 | negative |
| 8.93_500.3035m/z | s_Erythrobacter_sp002215495 | 0.01587039 | 0.94705273 | positive |
| 8.71_455.2295m/z | s_Microbacterium_foliorum_A | 0.01457789 | 0.95135973 | positive |
| 12.02_573.4507m/z | s_Sphingobium_yanoikuyae | 0.01456843 | 0.95139124 | positive |
| 8.93_500.3035m/z | s_Microbacterium_sp005774735 | -0.01433973 | 0.95215349 | negative |
| 6.95_167.0342m/z | s_Agrobacterium_pusense | 0.01353384 | 0.95690527 | positive |
| 6.73_462.1779m/z | s_Agrobacterium_pusense | 0.01203008 | 0.96197139 | positive |
| 6.72_464.1910m/z | s_Cutibacterium_acnes | -0.01053028 | 0.96485538 | negative |
| 2.15_274.0032m/z | s_Meiothermus_B_silvanus | -0.00854492 | 0.9714786 | negative |
| 8.93_353.2795m/z | s_Agrobacterium_sp900013535 | -0.00758588 | 0.97467866 | negative |
| 6.06_245.0920m/z | s_Microbacterium_sp005774735 | 0.0075188 | 0.97717764 | positive |
| 7.53_370.3060m/z | s_Agrobacterium_pusense | 0.0075188 | 0.97717764 | positive |
| 4.61_305.0341m/z | s_Microbacterium_foliorum_A | 0.00710285 | 0.97629054 | positive |
| 12.02_573.4507m/z | s_Cutibacterium_acnes | -0.00679506 | 0.9773177 | negative |
| 0.64_1157.2640m/z | s_Agrobacterium_pusense | 0.0060241 | 0.97989066 | positive |
| 10.43_374.2816n | s_Agrobacterium_pusense | 0.00601504 | 0.98224837 | positive |
| 6.95_123.0441m/z | s_Agrobacterium_pusense | 0.00451128 | 0.98731979 | positive |
| 4.76_356.0497m/z | s_Agrobacterium_sp900013535 | -0.00430855 | 0.98561673 | negative |
| 9.37_448.3053m/z | s_Agrobacterium_sp900013535 | -0.00430207 | 0.98563837 | negative |
| 6.72_464.1910m/z | s_Agrobacterium_pusense | -0.00150376 | 0.99746386 | negative |
| 15.25_612.5196m/z | s_Meiothermus_B_silvanus | 0 | 1 |  |
| 7.02_311.2324m/z | s_Sphingobium_yanoikuyae | 0 | 1 |  |
